# NPAHs and OPAHs in the atmosphere of two central European cities: Seasonality, urban-to-background gradients, cancer risks and gas-to-particle partitioning

**DOI:** 10.1016/j.scitotenv.2021.148528

**Published:** 2021-11-01

**Authors:** Céline Degrendele, Tjaša Kanduč, David Kocman, Gerhard Lammel, Adriana Cambelová, Saul Garcia Dos Santos, Milena Horvat, Petr Kukučka, Adéla Holubová Šmejkalová, Ondřej Mikeš, Beatriz Nuñez-Corcuera, Petra Přibylová, Roman Prokeš, Ondřej Saňka, Thomas Maggos, Denis Sarigiannis, Jana Klánová

**Affiliations:** a RECETOX Centre, Masaryk University, Czech Republic; b Department of Environmental Sciences, Jožef Stefan Institute, Slovenia; c Área de Contaminación Atmosférica, Centro Nacional de Sanidad Ambiental Instituto de Salud Carlos III, Spain; d Czech Hydrometeorological Institute, Czech Republic; e Atmospheric Chemistry & Innovative Technologies Laboratory, NCSR “Demokritos”, Greece; f Environmental Engineering Laboratory, Department of Chemical Engineering, Aristotle University of Thessaloniki, Thessaloniki, Greece; g HERACLES Research Centre on the Exposome and Health, Center for Interdisciplinary Research and Innovation, Thessaloniki, Greece; h University School of Advanced Study, Pavia, Italy

**Keywords:** Polycyclic aromatic hydrocarbons, Fine particles, Atmosphere, Spatial variations, Cancer risk

## Abstract

Derivatives of polycyclic aromatic hydrocarbons (PAHs) such as nitrated- and oxygenated-PAHs (NPAHs and OPAHs) could be even more toxic and harmful for the environment and humans than PAHs. We assessed the spatial and seasonal variations of NPAHs and OPAHs atmospheric levels, their cancer risks and their gas-to-particle partitioning. To this end, about 250 samples of fine particulate matter (PM_2.5_) and 50 gaseous samples were collected in 2017 in central Europe in the cities of Brno and Ljubljana (two traffic and two urban background sites) as well as one rural site.

The average particulate concentrations were ranging from below limit of quantification to 593 pg m^−3^ for Σ_9_NPAHs and from 1.64 to 4330 pg m^−3^ for Σ_11_OPAHs, with significantly higher concentrations in winter compared to summer.

In winter, the particulate levels of NPAHs and OPAHs were higher at the traffic site compared to the urban background site in Brno while the opposite was found in Ljubljana. NPAHs and OPAHs particulate levels were influenced by the meteorological parameters and co-varied with several air pollutants. The significance of secondary formation on the occurrence of some NPAHs and OPAHs is indicated. In winter, 27–47% of samples collected at all sites were above the acceptable lifetime carcinogenic risk. The gas-particle partitioning of NPAHs and OPAHs was influenced by their physico-chemical properties, the season and the site-specific aerosol composition. Three NPAHs and five OPAHs had higher particulate mass fractions at the traffic site, suggesting they could be primarily emitted as particles from vehicle traffic and subsequently partitioning to the gas phase along air transport. This study underlines the importance of inclusion of the gas phase in addition to the particulate phase when assessing the atmospheric fate of polycyclic aromatic compounds and also when assessing the related health risk.

## Highlights

• NOPAH particulate levels were generally correlated with PM, OC, EC, CO, Cu and Zn.
• In winter, NOPAH levels generally increased with NO_2_ but decreased with O_3_.
• Significant cancer risks from the inhalation of PAHs, NPAHs and OPAHs were found.
• Cancer risks might be significantly underestimated if the gas phase is not sampled.

## Graphical abstract

**Figure:**
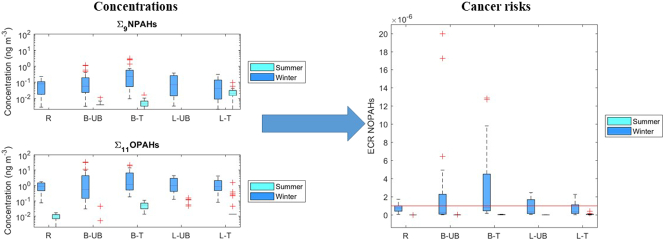

## 1 Introduction

At the global level, exposure to particulate matter (PM) in ambient air causes the premature death of 3.0–4.2 million people per year (Cohen et al., 2015; Landrigan et al., 2017; Lelieveld et al., 2015) and 0.40–0.79 million for Europe (EEA, 2020a; Lelieveld et al., 2019). The toxic properties of PM have been related to its organic constituents (Mauderly and Chow, 2008; Verma et al., 2015), particularly to the moderate polar and polar fractions (Idowu et al., 2019; Nováková et al., 2020; Valavanidis et al., 2008), such as most polycyclic aromatic hydrocarbons (PAHs) derivatives (Happo et al., 2008; Jalava et al., 2009). PAHs are a group of compounds formed via incomplete combustion from fossil fuels, coal, wood or biomass burning emitted via both natural (e.g. forest fires) and anthropogenic (e.g. traffic, domestic heating, industry) sources. In 2018, at the European level, up to 75% of PAH emissions were related to domestic heating and field burning of agricultural residues (EEA, 2020b) although underestimation of the emissions from the transport sector have been suggested (Ho et al., 2009; Kristensson et al., 2004; Tevlin et al., 2021; Whaley et al., 2020) which could lead to high uncertainties in regard to PAH emissions.

Besides the 16 PAHs which have been classified as priority compounds by the US Environmental Protection Agency several decades ago and which are regularly monitored worldwide, several hundreds of additional polycyclic aromatic compounds (PACs) exist in the environment that have been never or only poorly investigated (Andersson and Achten, 2015). Over the last decade, increasing attention has been given to PAH derivatives, i.e. the nitrated PAHs (NPAHs) and oxygenated PAHs (OPAHs), referred here as NOPAHs, which are often more toxic than their parent PAHs as they have direct toxic potency while parent PAHs require first an enzymatic activation (Abbas et al., 2018; Idowu et al., 2019; Lundstedt et al., 2014; Walgraeve et al., 2010). In particular, some NPAHs could be more mutagenic (i.e. up to 100,000 times) and carcinogenic (i.e. up to 10 times) than their parent PAHs (Arey et al., 1992; Durant et al., 1996; Yang et al., 2010). Concerning OPAHs, quinones produce reactive oxygen species, responsible of oxidative stress and can lead to allergic diseases and induce inflammatory reactions (Abbas et al., 2018; Sies et al., 2017; Sklorz et al., 2007). Moreover, OPAHs also have some endocrine-disrupting potential (Abbas et al., 2018; Idowu et al., 2019; Walgraeve et al., 2010). Unlike parent PAHs, NOPAHs are also secondarily formed in the atmosphere, by homogeneous or heterogeneous reactions of parent PAHs with atmospheric oxidants, photolysis or thermal conversion (Abbas et al., 2018; Bandowe and Meusel, 2017; Keyte et al., 2013). The importance of primary and secondary sources on the levels of individual NOPAHs is still poorly understood for most NOPAHs and is therefore limiting any successful emission regulation.

Information on the environmental fate of NOPAHs is limited by the current data available. Although the atmosphere has been the most studied environmental medium for NOPAHs, only a limited number of studies investigating NOPAH levels simultaneously at several sites impacted by different anthropogenic emissions (Albinet et al., 2007; Huang et al., 2014a; Wei et al., 2015b) were performed. However, such understanding of spatial gradients of NOPAHs is crucial to assess the impact of proximity to primary sources and the importance of secondary formation along atmospheric transport on their levels and therefore on their atmospheric fate and also to understand the spatial variability of human exposure to these toxic substances. Unlike for parent PAHs (Dachs and Eisenreich, 2000; Degrendele et al., 2020; Lohmann and Lammel, 2004; Shahpoury et al., 2016), the gas-particle partitioning of NOPAHs, which is crucial for atmospheric lifetime and, hence, long-range transport potential, has been incompletely studied (Huang et al., 2014a; Nežiková et al., 2021; Tomaz et al., 2016; Wei et al., 2015b). Although a significant fraction of NOPAHs can be present in the gaseous phase, particularly in summer (Lammel et al., 2020), and that gases penetrate more easily the blood system following uptake via inhalation (Wei et al., 2015b), this phase was not considered by most studies which evaluated the carcinogenic risk due to the inhalation of these substances (Alves et al., 2017; Bandowe et al., 2014) and therefore its contribution to the overall risk remains largely unknown.

In the framework of the ICARUS EU2020 project (‘Integrated Climate forcing and Air pollution Reduction in Urban Systems’), about 300 24-h air samples were collected in two central European cities at four sampling sites (i.e. traffic and urban background), as well as one rural site in winter and summer 2017 and were analyzed for NPAHs and OPAHs. The aim of this study is to provide novel atmospheric data on particulate NPAHs and OPAHs in Brno, Czech Republic and Ljubljana, Slovenia at each a traffic (T) and urban background (UB) sites, as well as at a rural site (in the Czech Republic). This study improves the current knowledge on the atmospheric fate of NOPAHs by assessing their seasonal and spatial variations as well as their gas-particle partitioning as a function of proximity to primary emissions. In addition, the impacts of NPAHs and OPAHs on human health via the characterization of the cancer risks due to the inhalation of these compounds were also evaluated.

## 2 Methodology

### 2.1 Air sampling

**Fig. 1:**
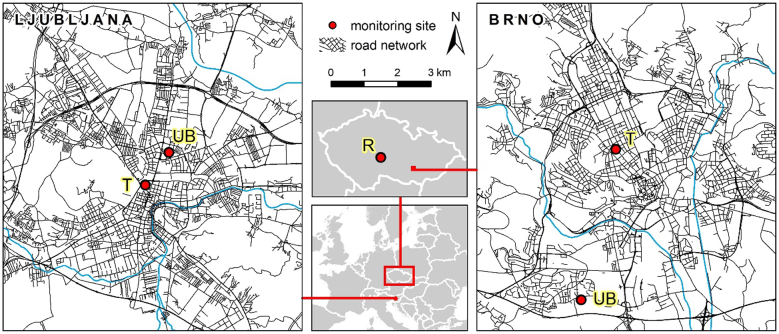
Maps of the sampling sites (T = traffic, UB = urban background and R = rural).

In this study, 24-h air samples of fine particles (i.e. <2.5 μm, PM_2.5_ sampling head) were collected within the ICARUS air sampling campaign (Saraga et al., 2021) in winter and summer 2017 at a traffic (T), and an urban background (UB) sites in Brno (N = 60 for both T and UB) and Ljubljana (N = 56 and 60 for T and UB), as well as at a rural (R) site in the Czech Republic (N = 16, Table S1 in the Supplementary information). Both traffic sites were located close to the city center, few meters away in Brno and about 100 m away in Ljubljana from a busy road with frequent congestion (Fig. 1). The UB sites were located in a residential area, <2 (Ljubljana) and <6 (Brno) km away from the traffic sites. In addition to domestic heating, these UB sites are also influenced by traffic sources, as major roads are less than 1 km away from these sampling sites (Fig. 1). The R site, i.e. the National Atmospheric Observatory Košetice (NAOK), reflects the regional background air, i.e. atmospheric pollution of central Europe; it is part of the European Monitoring Environmental Programme besides other atmospheric networks (Degrendele et al., 2018), located in the Czech Republic 130 km North-West of Brno. A major highway with a traffic volume of 40,000 cars per day is located about 7 km North-East from this R site (Holubová Šmejkalová et al., 2020). The use of a PM_2.5_ inlet is considered to be adequate to assess the levels of NOPAHs in the particulate phase as previous studies conducted at various sites (i.e. rural, marine, urban) and in different seasons have shown that most (i.e. >80%) of particulate NOPAHs present in the air were bound to fine particles (Albinet et al., 2008; Alves et al., 2016a; Besis et al., 2017; Kitanovski et al., 2020; Lammel et al., 2020, Lammel et al., 2017; Ringuet et al., 2012b). Depending on availabilities, high volume air samplers (HVAS, Digitel DH77, flow rate of 30 m^3^ h^−1^ at the Brno T site and the R site) or low volume air samplers (LVAS, Leckel MVS6 at Brno UB, LVS 3.1 Comde Derenda LVS/MVS at Ljubljana T and ECHO PM, Tecora at Ljubljana UB, all with a flow rate of 2.3 m^3^ h^−1^) were used. Particles were collected on Quartz Fiber Filters (QFFs) from Whatman (QM-A, 150 mm, Whatman, UK) for the Brno T and R sites, and from Pall (Non-Heat-treated Quartz, 2500 QAO-UP, 47 mm, Pall, USA) for all remaining sites. The sampled volumes were 752–829 m^3^ for those sites equipped with a HVAS and 38–55 m^3^ for those with a LVAS (Table S1). Only eight of the samples collected at the R site for each season underwent chemical analysis. Along particles, gas-phase was also collected on polyurethane foam (PUF) plugs (T2536, 110 × 50 mm for HVAS and 50 × 50 mm for LVAS, 0.030 g cm^−3^, Molitan a.s., Czech Republic) for seven or eight days simultaneously at each of the Brno sites and at the R site in both seasons (Table S1). The limited number of gaseous samples collected at each of these sites could limit their seasonal representability due to the high variability of meteorological conditions. Prior sampling, PUF plugs were pre-cleaned for 8 h by extraction with acetone and dichloromethane (DCM) each.

### 2.2 Sample preparation and analysis

After sampling and gravimetric measurements, the filters were cut using a pre-cleaned scalpel. Half of the filters underwent for the analysis of toxic metals using a non-destructive method (XRF X-Ray Fluorescence), prior to be used for the analysis of PAHs and their derivatives at the RECETOX Centre. All samples (i.e. half of QFFs and entire PUF plugs) were extracted with dichloromethane (DCM) using an automatic warm Soxhlet extractor (Büchi B-811, Switzerland). Surrogate recovery standards (1-nitronaphthalene-D7, 2-nitrofluorene-D9, 9-nitroanthracene-D9, 3-nitrofluoranthene-D9, 1-nitropyrene-D9 and 6-nitrochrysene-D11, Chiron, Trondheim, Norway) were spiked on each sample prior to extraction. The extracts were cleaned using a silica column (5 g of silica, 0.063–0.200 mm, activated at 150 °C for 12 h, 10% deactivated with water and 1 g of Na_2_SO_4_). The samples were loaded and eluted with 5 mL of *n*-hexane followed by 50 mL of DCM. The eluate volume was then reduced by a gentle stream of nitrogen in a TurboVap II (Caliper LifeSciences, USA) concentrator unit and transferred into a vial. PCB 121 (Absolute Standards Inc., Hamden, USA) was added as syringe standard prior analysis. The final volume was 200 μL. All the samples were analyzed by means of gas-chromatography (7890A GC, Agilent, USA) using a 30 m × 0.25 mm × 0.25 μm Rxi-5Sil MS column (Restek, USA) coupled to an atmospheric pressure chemical ionization tandem mass spectrometer (Xevo TQ-S MS, Waters, UK), GC-APCI-MS/MS. Additional details on the analytical methods used are available elsewhere (Lammel et al., 2020; Nežiková et al., 2021).

In total, 17 NPAHs (i.e. 1-Nitronaphthalene (1-NNAP), 2-nitronaphthalene (2-NNAP), 3-nitroacenaphthene (3-NACE), 5-nitroacenaphthene (5-NACE), 2-nitrofluorene (2-NFLN), 9-nitroanthracene (9-NANT), 9-nitrophenanthrene (9-NPHE), 3-nitrophenanthrene (3-NPHE), 2-nitrofluoranthene (2-NFLT), 3-nitrofluoranthene (3-NFLT), 1-nitropyrene (1-NPYR), 2-nitropyrene (2-NPYR), 7-nitrobenzoaanthracene (7-NBAA), 6-nitrochrysene (6-NCHR), 1,3-dinitropyrene (1,3-N_2_PYR), 1,6-dinitropyrene (1,6-N_2_PYR) and 1,8-dinitropyrene (1,8-N_2_PYR)) as well as 11 OPAHs (1,4-naphthoquinone (1,4-O_2_NAP), naphthalene-1-aldehyde (1(CHO)NAP), 9H-fluoren-9-one (9-OFLN), 9,10-anthraquinone (9,10-O_2_ANT), 1,4-anthraquinone (1,4-O_2_ANT), 9,10-phenanthroquinone (9,10-O_2_PHE), 11H-benzo-a-fluoren-11-one (BaOFLN), 11H-benzo-b-fluoren-11-one (BbOFLN), benzanthrone (BAN), benz(*a*)anthracene-7,12-dione (7,12-O_2_BAA) and 5,12-naphthacenequinone (5,12-O_2_NAC)) were quantified in the 298 samples collected (252 samples in the particulate phase and 46 in the gaseous phase).These compounds were selected to cover the most abundant species, possible derivatives of abundant parent PAHs, and based on available analytical standards.

### 2.3 QA-QC

In this study, 28 field blanks (21 QFFs and 7 PUFs) and 24 solvent blanks were analyzed as per samples. The levels of individual analytes in these blanks were generally below detection limit or low otherwise (<10% of average sample mass for detected compounds), suggesting minor contamination during sampling, transport and analysis. However, in some cases, such as for the most volatile NPAHs and OPAHs in summer or winter on QFFs or for the less volatile compounds on PUFs, the blank levels were close to those observed in the samples, reflecting their gas-particle partitioning in ambient air rather than contamination during sample preparation and analysis. The average recoveries (in QFFs and PUFs) were ranging from 83 ± 26% (1-NNAP) to 111 ± 43% (2-NFLT) (Table S2). The concentrations reported in this study were blank corrected by subtracting the average of the field blanks for each season and city, separately for QFFs and PUFs, but were not corrected for recoveries. The instrumental limits of quantifications (iLOQs) were determined from individual sample chromatograms as a signal-to-noise ratio of ten. In addition to iLOQs, the reported levels were also verified against the LOQs determined from the blanks (LOQb, defined as the average concentration in the field blanks plus three times their standard deviations), which were generally higher than the iLOQs. The accuracy of the analytical method has been assessed by two independent manners. Firstly, one extra filter was collected in winter 2016 on the rooftop of the Brno University Campus (very close to the Brno UB site) using the HVAS and was cut onto four pieces analyzed separately. The reported levels of those individual NPAHs and OPAHs which were higher than the iLOQs were consistent among the four samples (i.e. within 10%, Table S3). Secondly, three pre-clean PUFs spiked with standards of all targeted analytes were analyzed and the average recoveries were ranging from 53 ± 5% (1-NNAP) to 133 ± 21% (1,4-O_2_NAP) (Table S4).

### 2.4 Additional data

For each of the sampling sites, meteorological data (i.e. temperature, wind speed, wind direction and relative humidity) were obtained from the Czech Hydrometeorological Institute (https://www.chmi.cz/), the Slovenian Environment Agency (http://meteo.arso.gov.si) and the Municipality of Ljubljana, when available (Table S5). In addition to the targeted analytes, PAHs, PM_2.5_, toxic metals (e.g. Cu, Zn), elemental (black) and organic carbon, anions and cations were also determined in these samples (see Saraga et al., 2021, for more details). Moreover, the concentrations of additional air pollutants (e.g. NO, NO_2_, CO, PM_1_) were obtained from air monitoring networks (https://www.ljubljana.si/sl/moja-ljubljana/varstvo-okolja/stanje-okolja/kakovost-zraka/), when available (Table S5). The Pearson correlations between the NOPAHs levels and both the meteorological parameters and the concentrations of air pollutants were assessed in order to better understand the inter-sample variability for each site and each season.

### 2.5 Cancer risk assessment

In this study, carcinogenic risk assessment from exposure to PAH mixtures was done using the potency equivalent factor (PEF) approach (USEPA, 2010), which takes into account the relative potencies of individual compounds (Hayakawa, 2016). For each sample, the concentrations of benzo(*a*)pyrene equivalents (BaP_eq_) were calculated by multiplying the concentration of individual compounds (C_i_) with their potency equivalent factors (PEF_i_) as:

$${\mathrm{BaP}}_{\mathrm{eq}}=\Sigma \;C_{i}\;{\mathrm{PEF}}_{i}$$

PEF values were available for 16 PAHs, 11 NPAHs and 2 OPAHs (Table S6). The cancer risks from inhalation of the targeted substances were estimated as:

$$\mathrm{ECR}={\mathrm{BaP}}_{\mathrm{eq}}\times {\mathrm{UR}}_{\mathrm{BaP}}$$

where ECR is the lifetime excess cancer risk, UR_BaP_ is the unit risk of BaP, which represents the number of people at risk of contracting cancer from inhalation of BaP_eq_ concentration of 1 ng m^−3^ over a life time of 70 years and equals to 8.7 × 10^−5^ (WHO, 2000).

## 3 Results and discussion

### 3.1 Levels of particulate NOPAHs

Unlike 11 OPAHs which were all frequently detected on the filters, only 9 out of the 18 targeted NPAHs were consistently found in the particulate phase (Figs. S1–S2). Indeed, five targeted NPAHs (i.e. 9-NPHE, 1,8-N_2_PYR, 6-NBAP, 3-NACE and 5-NACE) were never detected, while four NPAHs (i.e. 1,3-N_2_PYR, 1,6-N_2_PYR, 2-NFLN and 6-NCHR) were rarely found (i.e. in 7, 1, 2 and 2 samples, respectively), mainly at the Brno traffic site. The lack of detection of 5-NACE, which is emitted from biomass and coal combustion as well as traffic (Alves et al., 2016b; Huang et al., 2014b; Vicente et al., 2017) and is also an oxidation product of acenaphthene with OH and NO_x_ (Sauret-Szczepanski and Lane, 2004) is surprising as it was the compound most found in particulate samples from three Southern European cities contributing for more than 80% of the NPAHs concentrations (Alves et al., 2017). The low detection frequencies of the N_2_PYR isomers is consistent with previous studies done at urban (Albinet et al., 2008; Alves et al., 2017; Lammel et al., 2020) or rural (Nežiková et al., 2021) sites, but also with measurements of fresh traffic emissions (Alves et al., 2016b). The detection frequencies of individual NOPAHs were obviously influenced by the season, distance to emissions (or type of site) and the amount of volume sampled. Indeed, NOPAHs had higher detection frequencies in winter compared to summer. In summer, few NPAHs such as 2-NNAP, 3-NPHE, 2-NFLT, 3-NFLT, 1-NPYR and 2-NPYR were found almost only at the traffic sites. On the other hand, several OPAHs (e.g. 1,4-O_2_NAP, BaOFLN, BbOFLN, BAN) were usually found in summer >LOQ at R, but mostly found <LOQ at the Ljubljana sites, obviously influenced by a volume up to 20 times lower of air sampled (Figs. S1–S2, Tables S7–S10). This highlights the importance of long-range atmospheric transport of some OPAHs to the rural site. In the following, we will refer to Σ_9_NPAHs as the sum of 1-NNAP, 2-NNAP, 9-NANT, 3-NPHE, 2-NFLT, 3-NFLT, 1-NPYR, 2-NPYR and 7-NBAA.

The average particulate concentrations of Σ_9_NPAHs across all sites ranged from 79.4 to 593 pg m^−3^ in winter and from <iLOQ to 25.0 pg m^−3^ in summer (Tables S7–S8). Σ_11_OPAHs exhibited atmospheric levels about one order of magnitude higher than those of Σ_9_NPAHs, with average particulate concentrations for individual sites ranging from 915 pg m^−3^ to 4330 pg m^−3^ in winter and from 1.64 pg m^−3^ to 102 pg m^−3^ in summer (Tables S9–S10). Compared to parent PAHs (data not shown), the levels of NPAHs and OPAHs were significantly lower (i.e. about two and one orders of magnitude, respectively). In addition, in terms of mass, NOPAHs represent only a negligible fraction (i.e. 3 ppb – 1 ppm) of the ambient fine particles. Although the number of individual NOPAHs investigated by previous studies or the phase sampled is different, the levels of NOPAHs found in this study are consistent with those reported from polluted or urban air in Europe, i.e. up to a few ng m^−3^ and 20 ng m^−3^ for NPAHs and OPAHs, respectively, (Bandowe and Meusel, 2017; Besis et al., 2017; Kitanovski et al., 2020; Lammel et al., 2020; Tomaz et al., 2016), but lower than those found in Asia, i.e. up to 12 ng m^−3^ for NPAHs and up to 120 ng m^−3^ for OPAHs, respectively (Huang et al., 2014a; Wei et al., 2015a) or within a tunnel affected by fresh exhaust emissions, i.e. up to 20 ng m^−3^ and 50 ng m^−3^ for NPAHs and OPAHs, respectively (Alves et al., 2016b).

**Fig. 2:**
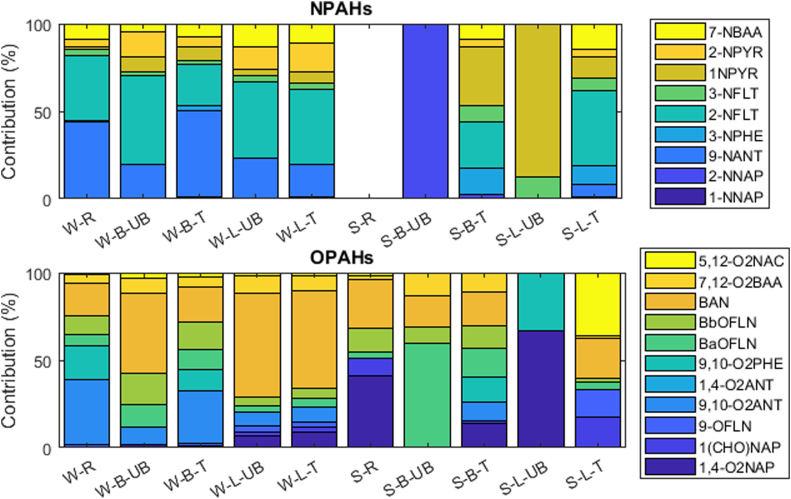
Average composition profile of particulate NPAHs and OPAHs across sites (B = Brno, L = Ljubljana, R = rural, UB = urban background, T = traffic) and seasons (S = summer, W = winter).

The composition profiles of particulate NOPAHs are presented as an average in Fig. 2 while their temporal variations are shown in Figs. S3–S4. Overall, strong seasonality in the composition profile is observed for both NPAHs and OPAHs although there are more uncertainties in summer due to the low levels, while differences between cities were observed only for OPAHs. Concerning NPAHs, in winter, similar composition profiles were observed for all sites, with a dominance of 9-NANT and 2-NFLT, contributing to Σ_9_NPAHs on average for 19–49% and 23–51%, respectively. In summer, the composition profiles could only be determined at the traffic sites due to low detections at the remaining sites. At the Brno T site, 1-NPYR, 2-NFLT, 3-NPHE dominated the particulate NPAHs levels, accounting on average for 34%, 26% and 15%, respectively. At the Ljubljana T site, 2-NFLT and 7-NBAA showed the highest concentrations accounting on average for 43% and 14%, respectively. It is interesting to note that 9-NANT, which is primarily emitted (diesel, coal, wood burning) (Bandowe and Meusel, 2017; Inomata et al., 2015) and secondarily formed only in the particulate phase (Huang et al., 2014a; Xing et al., 2020), contributed with significant mass fraction in winter at all sites but was not detected in summer in Brno and at low concentrations in Ljubljana, which may suggest that photolysis significantly limits its lifetime compared to other NPAHs (Lammel et al., 2020). Photolability is not unlikely for NPAHs with peri-H atoms, such as 9-NANT (2) and 1-NPYR (1 peri-H; Fan et al., 1995). The contribution of 1-NPYR to Σ_9_NPAHs was generally small (i.e. <12%), except in summer at the Brno traffic site where it contributed on average for 34%, while this compound was previously found to dominate NPAH levels at traffic sites (Besis et al., 2017; Ringuet et al., 2012a). Concerning the seasonal variations, the contribution of 9-NANT was decreasing from winter to summer, similar to previous studies (Nežiková et al., 2021; Tomaz et al., 2016), and is attributed to the limited secondary formation compared to other NOPAHs in summer and to phase partitioning. Regarding OPAHs, in winter, the two sites in Ljubljana showed similar composition profiles with BAN showing the highest contribution (56–59%), followed by 7,12-O_2_BAA (8–10%) and 9,10-O_2_ANT (8–9%). In Brno, the UB site was dominated by BAN (46%), BbOFLN (18%) and BaOFLN (13%) while the T and R sites, which had similar profiles, were dominated by 9,10-O_2_ANT (30–37%), BAN (19–20%) and to a lower extent also by 9,10-O_2_PHE (12–19%) which was only detected in winter at these two sites.

### 3.2 Seasonal and spatial variations of particulate NOPAHs

**Fig. 3:**
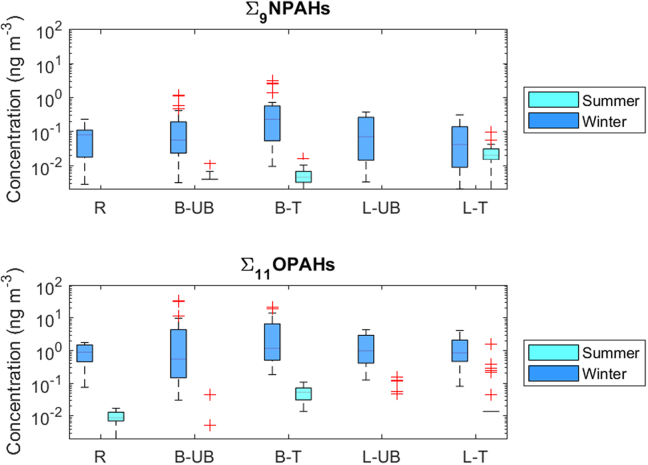
Boxplots of the seasonal variations of the particulate concentrations of Σ_9_NPAHs and Σ_11_OPAHs. B, L, R, UB and T denote Brno, Ljubljana, rural, urban background and traffic, respectively.

Significant seasonal variations, with higher particulate levels in winter compared to summer, were observed for all individual NOPAHs (except few OPAHs at the Ljubljana T site) as well as for Σ_9_NPAHs and Σ_11_OPAHs, (Fig. 3, Table S11). Different seasonal variations were rarely reported, for example for three Southern European cities which had similar levels of OPAHs in winter and summer (Alves et al., 2017), or more recently at an urban site in the Arctic where NOPAHs levels were higher in spring (Drotikova et al., 2021), which reflects specific primary emissions at these sites. The winter-to-summer (W/S) ratios of the particulate concentrations across all sites were 1.83–149 and 0.59–121 for individual NPAHs and OPAHs, respectively (Table S11). These ratios are generally higher than those reported for other parts of the world (i.e. 3–8, (Albinet et al., 2008; Bandowe et al., 2014; Tomaz et al., 2016)). Higher levels of NOPAHs in winter compared to summer are related to (Bandowe and Meusel, 2017; Tang et al., 2014; Tomaz et al., 2016; Zhang et al., 2018): (i) higher emissions in winter from domestic heating, but also possibly to the influence of ambient temperature on NOPAHs exhaust emissions as it has been found to be important for organics (Joumard et al., 2009) including PAHs (Hays et al., 2017), and even few nitro-PAHs (Zielinska et al., 2004). (ii) lower atmospheric boundary layer height in winter, (iii) increased partitioning to particulate phase at lower temperatures and (iv) enhanced photochemical degradation in summer related to higher levels of hydroxyl radicals.

The spatial gradient between the different sites were assessed in both Brno and Ljubljana (Tables S12–S14) using the samples collected simultaneously site by site. In winter, the spatial distributions of particulate NOPAHs differed between Brno and Ljubljana. In Brno, a strong traffic to rural gradient was observed, with the highest concentrations of NOPAHs at the traffic site and the lowest at the rural site (Fig. 3). Indeed, the particulate concentrations measured at the traffic site were on average 4.0 and 2.7 times higher for Σ_9_NPAHs and Σ_11_OPAHs, respectively, than at the UB site, which were in turn, 5.3 and 4.9 times higher than those at the rural site (Tables S12–S14). The highest ratios between the traffic and the urban background sites (i.e. T/UB) were found for individual compounds which are known to be primarily emitted from traffic (e.g. 1-NPYR, 9-NANT, Table S13, Bandowe and Meusel, 2017). On the other hand, at Ljubljana, the winter concentrations of NOPAHs measured at the UB site were usually higher than those at the T site (Table S12). Indeed, the average T/UB ratios were 0.55 and 0.46 for Σ_9_NPAHs and Σ_11_OPAHs, respectively, and they ranged from 0.55 to 2.07 for individual compounds although their medians were usually lower than unity (Table S12). In summer, for both cities, the concentrations of NOPAHs were the highest at the traffic sites (Tables S12–13), although the levels and the number of compounds detected at all sites were rather low, limiting therefore the assessment of the spatial gradients. The differences observed between Brno and Ljubljana in the spatial distribution of NOPAHs in winter between the traffic and the urban background sites suggest differences in the extent of primary sources. Emissions from domestic heating are unlikely to be higher in Ljubljana than in Brno due to the higher winter temperatures in Ljubljana (Table S5) but also to the higher usage of wood and coal in Brno (Neuhäuser, 2017), which are expected to be associated with higher emissions of NOPAHs (Huang et al., 2014b; Shen et al., 2013). Possibly, traffic emissions of NOPAHs might be more significant in Brno as diesel vehicles, which have higher emissions of NOPAHs than petrol vehicles (Zielinska et al., 2004), are more common in Brno than in Ljubljana (ATEM, 2016; Logar et al., 2018). Moreover, among personal cars, the vehicle fleet in Brno is significantly older than in Ljubljana (Fig. S5), which could lead to higher NOPAH emissions (Zielinska et al., 2004), particularly considering that the average temperature during the winter campaign was significantly lower in Brno compared to Ljubljana (Table S5). In addition, the proximity to traffic has a larger gradient between the traffic and UB sites in Brno (i.e. 5 m and 800 m) compared to Ljubljana (i.e. 100 m and 250 m respectively), which could explain the spatial differences between these two cities.

### 3.3 Relationships with meteorological parameters and air pollutants

The influence of meteorological parameters as well as the concentrations of particulate matter and air pollutants on the levels of particulate NOPAHs has been assessed (Tables S15–S24, Fig. S6). In general, the correlations found between NOPAHs levels and the different parameters were more significant in winter compared to summer, related to the lower detection frequencies of individual NOPAHs and the low levels in summer. Concerning the meteorological parameters, in winter, the increase in wind speed and temperature lead to significant decrease in NOPAH levels at all sites where these were measured, highlighting the influence of atmospheric dispersion and temperature-dependency of primary emissions on NOPAH levels. NOPAHs concentrations generally showed significant positive correlation with particulate mass (PM_2.5_ but also PM_10_ sometimes), particularly in winter and at the traffic sites (Tables S15–S24). At the Brno UB site, which measured both PM_1_, PM_2.5_ and PM_10_ in winter, higher correlations were observed with the finest particles, consistent with the fact that NOPAHs are usually sorbed to submicron particles (Besis et al., 2017; Kitanovski et al., 2020; Lammel et al., 2020; Ringuet et al., 2012b). Interestingly, the Pearson coefficients observed between NOPAH levels with OC and EC were significantly higher (p < 0.05) than with PMs, particularly in winter and for the traffic sites. This is expected as the mass size distributions are similar and is possibly reflecting that these materials effectively sorb polycyclic aromatic compounds (Bandowe et al., 2014). In addition, in winter, the correlations of NOPAHs were more pronounced with OC than with EC at both the Brno UB and T sites (Fig. S6, Tables S16–S17) while in Ljubljana, the Pearson correlation coefficients did not differ much between OC and EC (Tables S18–S19). On the other hand, in summer, the relationship was usually clearer with EC compared to OC at both traffic sites for NPAHs but only at Brno traffic site for OPAHs (Tables S22 and S24). Given that EC only originates from incomplete combustion of biomass and fossil fuels which characterize primary emission while OC is also from transformation of organic constituents by secondary aerosol formation (Fuzzi et al., 2006; Pöschl, 2005), the results found here support the perception of the primary origin of NOPAHs at the traffic sites in summer or at the Ljubljana sites in winter, while in the case of Brno, non-traffic related sources had also an influence on the intra-sample variability of NOPAH levels in winter, including those of 1-NPYR. Significant correlations between some NOPAHs and oxidants (i.e. O_3_, NO_2_) were found in winter but generally not in summer (Tables S15–S24). Indeed, in winter, NOPAH levels increased with NO_2_, but decreased with O_3_, which is consistent with previous studies (Albinet et al., 2007; Huang et al., 2014a; Ringuet et al., 2012a). This suggests that the reaction with O_3_ could be an important sink regarding NOPAHs atmospheric fate (Albinet et al., 2007), while the one with NO_2_ or other oxidants that co-vary with NO_2_ suggest their formation from parent PAHs (Bamford and Baker, 2003). In addition, NOPAHs levels were significantly positively correlated with CO, a marker of vehicular emissions, but also with Cu and Zn which in polluted air are tracers of non-exhaust break wear (Adamiec et al., 2016) and tire wear (Councell et al., 2004), respectively. This suggests that traffic is an important source of NOPAHs and that post emissions, NOPAHs bound to particles are affected in the same manner as other particles by dust resuspension (Wei et al., 2015a). Finally, NOPAH levels were also correlated in winter at Brno UB and the R site with SO_2_ levels, which are dominated by regional atmospheric transport (Aas et al., 2019). This highlights that at these sites which are not or less affected by primary traffic emissions, at least regional long-range atmospheric transport has some influence on the NOPAHs levels found, in agreement with a previous study (Huang et al., 2014a).

### 3.4 Source identification

In this study, the correlations between the particulate levels of the different compounds studied and well-characterized (so-called diagnostic) ratios were investigated to distinguish between primary and secondary sources or even to distinguish between diesel and petrol exhaust emissions. Significant (p < 0.05) and strong (r = 0.64–0.98) correlations were found between Σ_16_PAHs, Σ_9_NPAHs and Σ_11_OPAHs in winter at all sites, while in summer it was at both traffic sites for Σ_9_NPAHs (i.e. r = 0.57–0.81) and at the Brno traffic site for Σ_11_OPAHs (r = 0.73) . In addition, Pearson correlations were also assessed between individual NOPAHs (Tables S25–S31). Several NOPAHs (i.e. BaOFLN, 9-NANT and 7-NBAA) were generally not or poorly correlated with 1-NPYR. 1-NPYR is a compound classified as probably carcinogenic to humans (i.e. Group 2A) and a marker of primary emissions from traffic in general (Srivastava et al., 2021), but more specifically from diesel vehicles (Bandowe and Meusel, 2017; Karavalakis et al., 2012, Karavalakis et al., 2011; Kawanaka et al., 2007) although this compound was not found in the exhaust of more recent diesel vehicles (Alves et al., 2016b). The lack of correlations found suggests that these specific NOPAHs do not come from diesel emissions but from other combustion sources. A previous study has suggested that BaOFLN was emitted from petrol exhaust (Albinet et al., 2007), which tends to support the findings found in this study. On the other hand, 7,12-O_2_BAA was always significantly (p < 0.05) correlated with 1-NPYR highlighting a possible diesel origin, as previously suggested (Albinet et al., 2007). Moreover, we can note that 9,10-O_2_ANT, BaOFLN, 9-NANT, 7-NBAA, 9-OFLN and 7,12-O_2_BAA were in several cases significantly correlated with 2-NFLT and 2-NPYR, which are known to be only secondarily formed in the atmosphere via photochemical reactions with the hydroxyl radical (day) or the nitrate radical (night) (Arey et al., 1986; Keyte et al., 2013). Given that most of these compounds were also positively correlated with O_3_ (Tables S15–S24), whose high concentrations enhance the gas-phase and heterogeneous nitration of PAHs (Feilberg et al., 2002), we suggest that they might have some secondary origin in addition to primary sources. This secondary origin was previously suggested for 9,10-O_2_ANT (Albinet et al., 2007; Perraudin et al., 2007), 9-NANT (Albinet et al., 2008; Drotikova et al., 2020; Lin et al., 2015), 7-NBAA and 7,12-O_2_BAA (Ringuet et al., 2012a).

**Fig. 4:**
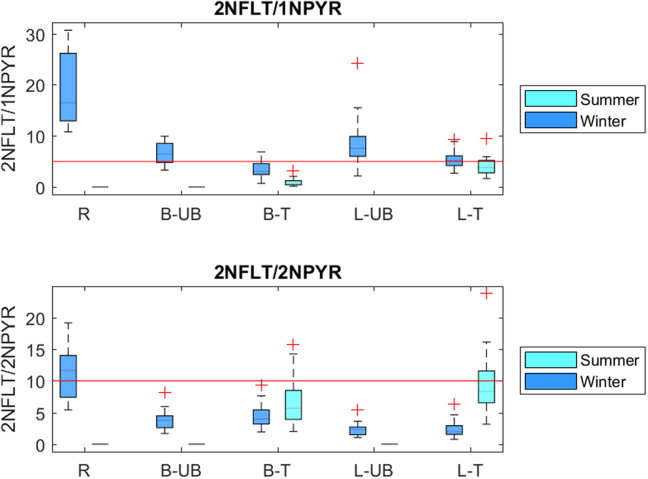
Ratios of selected individual NPAHs. B, L, R, UB and T denote Brno, Ljubljana, rural, urban background and traffic, respectively. The horizontal red lines indicate threshold values (see text). (For interpretation of the references to color in this figure legend, the reader is referred to the web version of this article.)

In winter, the traffic site in Brno was influenced by primary emissions as the ratio 2-NFLT/1-NPYR was smaller than 5 (Ciccioli et al., 1989) while the secondary emissions were dominant at all the remaining sites, although at Ljubljana, the UB site had higher ratio than the traffic site (Fig. 4). The clear spatial gradient observed in Brno, with higher ratio at the sites distanced from primary sources, indicates that NPAHs are primarily emitted at traffic sites but also secondarily formed during atmospheric transport to distanced sites (Ringuet et al., 2012a). The average values of the 2-NFLT/2-NPYR concentration ratio were ranging between 2 and 13 for all sites and both seasons (Fig. 4), pointing out day time formation (Arey et al., 1986; Keyte et al., 2013), Although this ratio was highest at the rural site, no clear spatial variations were observed while higher values were generally found in summer at both traffic sites.

For 1,4-O_2_NAP/NAP, 1-NNAP/NAP, 1-NPYR/PYR, 7-NBAA/BAA and to a lesser extent, also 2-NFLT/FLT, 2-NPYR/PYR and 9-NANT/ANT, a spatial gradient was generally observed with higher ratios at the traffic sites and the lowest at the rural site (Fig. S7 and Tables S32–S33). Considering the secondary formation along atmospheric transport of NOPAHs, one may expect higher ratios in summer (enhanced photochemistry) and in aged air masses, as the PAH derivatives are expected to degrade slower than their parent compounds (Alam et al., 2015; U.S. EPA, 2012). Note that primary sources contributed to the derivatives abundance, too (except for 2-NFLT and 2-NPYR). Indeed, higher ratios of 9,10-O_2_PHE/PHE, 1-NPYR/PYR, 3-NPHE/PHE and 7-NBAA/BAA were observed in summer compared to winter, similar to other studies (Alves et al., 2017; Bandowe et al., 2014; Harrison et al., 2016).

### 3.5 Cancer risks

The average cancer risks due to particulate NOPAHs were ranging for all sites from 1.83 × 10^−10^ to 2.94 × 10^−6^ (Table S34, Fig. S8), and were slightly lower or above the acceptable lifetime carcinogenic risk (i.e. 1 × 10^−6^) in winter while in summer the risks were negligible (i.e. <4 × 10^−8^). In winter, 27–47% of samples collected at all sites were above the acceptable lifetime carcinogenic risk. The main contributors to ECR were BAN, 9,10-O_2_ANT, 2-NFLT and 1-NPYR contributing on average for 27%, 26%, 25% and 20% although large variations were observed according to the season or site considered. 6-NCHR and 1,6-N_2_PYR, which have the highest PEFs among all targeted compounds (i.e. 10, Table S6), contributed to 30–33% of the ECR in the few samples in which they were detected. When considering both NOPAHs and PAHs, 77–100% of all samples collected exceeded the acceptable lifetime carcinogenic risk (Table S35). This suggests significant health risk associated with the inhalation of polycyclic aromatic compounds in these two Central European cities. This is consistent with a recent study showing that 70% of the global population breathes air which exceeds the safe threshold level, with higher risks in China, India, Central and Eastern Europe (Kelly et al., 2021). The risks estimated in this study for both PAHs and NOPAHs are somewhat similar to those previously reported in several locations in China (Bandowe et al., 2014; Wei et al., 2015a), Porto, Florence or Athens (Alves et al., 2017) but higher than those found at Langfang, China (Zhao et al., 2018) or Grenoble, France (Tomaz et al., 2016). In this study, we find that the contribution of NOPAHs to the PAC mass (up to 30%) was significantly higher than the contribution of NOPAHs to the PAC carcinogenicity (up to 1%, Fig. S10). This is different than what has been found elsewhere (e.g. a higher contribution of NPAHs to the PAC carcinogenicity (3.5%) than to PAC mass (0.7%), Huang et al., 2014a), and particularly in a recent study at the global level estimating that NPAHs alone contributed to 15–20% of the carcinogenic potential of PAH mixtures (Kelly et al., 2021).

Estimates of cancer risks should be taken with caution as they are associated with several uncertainties. Firstly, it was assumed that all NOPAHs present in the air are bioaccessible. However, a recent study simulating uptake of particulate NOPAHs onto lung fluids found that their bioaccessibility was generally relatively low (i.e. up to only a few %) (Lammel et al., 2020). Secondly, this approach assumed that the interaction of some of the targeted compounds is additive rather than synergistic or antagonistic (Abbas et al., 2018; Jung et al., 2010). Moreover, PEF values were available for only 13/28 NOPAHs investigated, particularly limited for OPAHs (Table S6). This ignorance may cause significant underestimation of the toxicity of PACs (Kelly et al., 2021; Samburova et al., 2017). Moreover, we used the unit risk factor set by WHO (WHO, 2000) while a different value (i.e. 1.1 E-06) (OEHHA, 1994) is also adopted, which would lead to about 80 times lower risks (Alves et al., 2017).

### 3.6 Gas-particle partitioning of NOPAHs

**Fig. 5:**
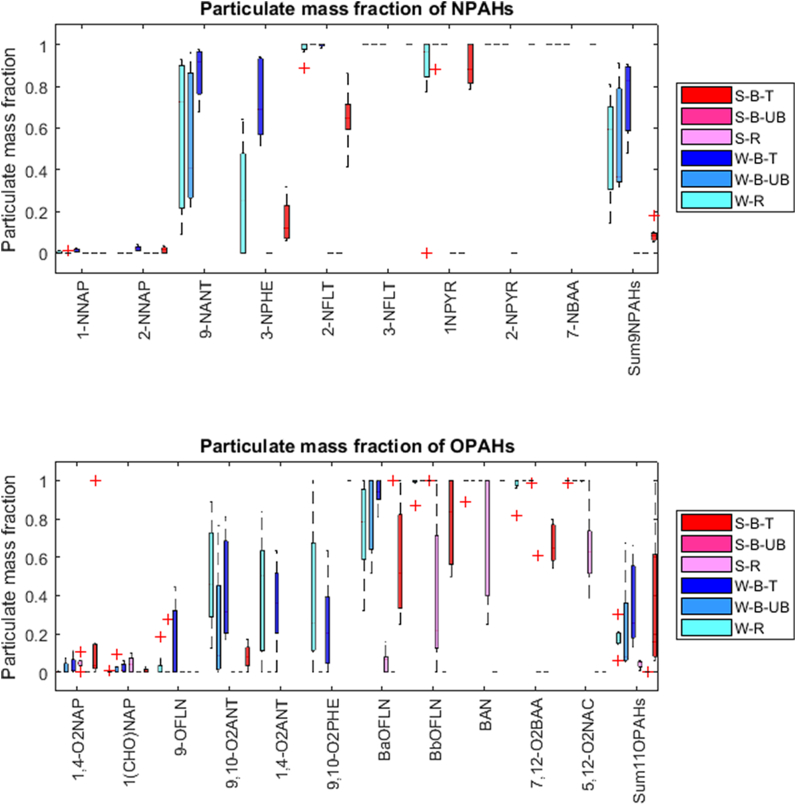
Particulate mass fractions of NOPAHs at the Czech. sites. S, W, B, R, T and UB denote summer, winter, Brno, rural, traffic and B, L, R, UB and T denote Brno, Ljubljana, rural, urban background and traffic, respectively.

The particulate mass fractions of individual NOPAHs are presented in Fig. 5 while individual data and statistics are available in Tables S36–S45. The reported particulate mass fractions correspond to the sampling design, which focused on fine particles (i.e. PM_2.5_). NOPAH mass size distributions have been found centering in the PM_2.5_ size fraction at both urban and rural sites (Albinet et al., 2008; Alves et al., 2016a; Besis et al., 2017; Kitanovski et al., 2020; Lammel et al., 2020, Lammel et al., 2017; Ringuet et al., 2012b). Therefore, particulate mass fractions might only slightly underestimate the real particulate mass fractions would particles of all sizes have been sampled. Overall, in this study, Σ_9_NPAHs were predominantly found on particles in winter (Θ = 0.52 ± 0.24, 0.55 ± 0.26 and 0.74 ± 0.17 for R, UB and T, respectively) but mainly in the gas phase in summer (Θ = 0 for R and UB and 0.09 ± 0.04 for T) while OPAHs were mainly found as gases for all sites and seasons investigated (i.e. Θ < 0.36). These values should be taken with caution as they are obviously dominated by the most abundant compounds, and do not reflect the large variations in the particulate mass fractions observed across individual NOPAHs (Fig. 5, Tables S38–S39). As 5-NACE and 7-NBAA were detected only in the gaseous and particulate phases, respectively, at all sites in both seasons, their particulate mass fractions (i.e. Θ = 0 or 1) are not further discussed. As expected, the gas-particle partitioning of individual NOPAHs was influenced by their vapor pressure, with higher Θ for compounds with lower vapor pressures (Fig. 5). For example, while the NNAPs isomers or 1(CHO)NAP were mainly found on the gas phase (i.e. Θ < 0.1), 3-NFLT and 1-NPYR were generally found on particles (Θ > 0.9), which is in agreement with previous studies (Albinet et al., 2007; Drotikova et al., 2020; Harrison et al., 2016; Huang et al., 2014a; Nežiková et al., 2021; Zhang et al., 2018). Slight differences in the particulate mass fractions of 9,10-O_2_ANT, 1,4-O_2_ANT and 9,10-O_2_PHE, which have the same molecular weight but different vapor pressures, were observed (Fig. 5, Table S39), highlighting the importance of the molecular interactions occurring with the different constituents of particulate matter (Shahpoury et al., 2016).

Although several NOPAHs could not be quantified in summer due to the low levels, significant seasonal variations in the gas-particle partitioning were found, with higher Θ in winter compared to summer for 3-NPHE, 2-NFLT, 1-NPYR, 9,10-O_2_ANT, BaOFLN, BbOFLN, BAN (only at the rural site), 7,12-O_2_BAA, and 5,12-O_2_NAC but not for the remaining compounds (Fig. 5). For example, at the traffic site, Θ of 3-NPHE and 9,10-O_2_ANT were 0.74 ± 0.19 and 0.44 ± 0.27 in winter, while they were 0.15 ± 0.01 and 0.08 ± 0.06 in summer. This seasonality in gas-particle partitioning of NOPAHs has been reported from European and Asian sites (Albinet et al., 2008; Bandowe and Meusel, 2017; Tomaz et al., 2016; Wei et al., 2015b) and is dominated by the influence of ambient temperature on vapor pressure of NOPAHs. The reported particulate mass fractions of NOPAHs from this study as well as their seasonal variations are consistent with those reported recently from our rural site or from another urban site in the Czech Republic (Lammel et al., 2020; Nežiková et al., 2021). Some unexpected seasonal variations in the particulate mass fraction were found. Indeed, the particulate mass fraction of 1,4-O_2_NAP at the traffic site in summer varied considerably (i.e. Θ ranging from 0 to 1) which resulted in a higher average particulate mass fraction in summer (Θ = 0.17 ± 0.34) compared to winter (Θ = 0.04 ± 0.04). Similarly, 9,10-O_2_PHE was only found in the particulate phase in summer at the traffic site (i.e. Θ = 1) while it had an average Θ of 0.24 ± 0.25 in winter. This behavior has been previously reported for 1,4-O_2_NAP and was attributed to slow relaxation to equilibrium in the vicinity of the source, which could be particularly important when the emission temperature is higher than the ambient temperature (Lammel et al., 2020).

Significant spatial distributions in the gas-particle partitioning of some NOPAHs were found. Indeed, higher particulate mass fractions were observed in at least one of the seasons investigated at the traffic site compared to the urban background or rural sites for three NPAHs (i.e. 9-NANT, 3-NPHE and 1-NPYR) and five OPAHs (i.e. 9,10-O_2_ANT, BaOFLN, BbOFLN, BAN and 7,12-O_2_BAA, Fig. 5, Tables S38–S39). Similar variations were reported for three and four rings NPAHs which had higher Θ in polluted air than in clean air (Lammel et al., 2017). Given the small distance between the UB and T sites, photolysis and particulate matter abundance (i.e. PM_2.5_ levels were not statistically different between the UB and T sites) were similar. Hence, the higher particulate mass fractions found for these specific NPAHs and OPAHs at the traffic site suggest that these compounds are primarily emitted from traffic as fine particles and are subsequently partitioning to the gas phase along transport. The same trend is not necessarily found when comparing urban and the rural background site: The winter particulate mass fractions of 9,10-O_2_ANT, 1,4-O_2_ANT and 9,10-O_2_PHE at the rural background site were slightly higher than those at other sites. This is not inconsistent, though, as the aforementioned parameters, photolysis, particulate matter abundance and chemical composition, can vary significantly on this spatial scale.

### 3.7 Importance of the gaseous phase

Sampling only the particulate phase, which is commonly done in air pollution monitoring, creates serious bias in characterization and, hence, of assessment of atmospheric fate of and human exposure to semivolatile organic compounds such as NOPAHs. Inclusion of the gas phase led to higher detection frequencies of the low molecular weight NOPAHs (e.g. 1-NNAP, 1,4-O_2_NAP) (Tables S46–S47). For both NPAHs and OPAHs, the composition profile differs significantly between the total (gaseous + particulate) and the particulate phase, due to the higher contribution of the most volatile NOPAHs and significant seasonal variations (Fig. S9). Moreover, the seasonal variations of the total mass concentration is found lower, except for few OPAHs (i.e. 1,4-O_2_NAP, 1(CHO)NAP and 1,12-O_2_BAA) (Table S48). 5-NACE, which was found only in the gaseous phase (Table S38), differs from the other NOPAHs as it had levels significantly higher in summer compared to winter at both the traffic and the rural site (i.e. W/S = 0.13 for both, Table S48). This must be related to the photochemical formation from the parent acenaphthylene or revolatilisation from surfaces, or both. In addition, at the rural site in summer, the gaseous concentrations of 5-NACE, 3-NPHE, 2-NFLT and BaOFLN were positively correlated (r = 0.74–0.88, p < 0.05) with the average temperature which showed an amplitude of 10 °C, which points again at secondary formation or revolatilisation from surfaces. Generally, monitoring of the particulate phase only ignores gaseous emission sources such as re-volatilisation of semivolatiles stored in surface compartments (soil, surface waters). A previous study in summer in central Europe has shown that for some light PAHs, this process was significant and controlling their ambient concentrations (Degrendele et al., 2016) but this process has never been investigated for NOPAHs so far.

Similar spatial gradients were observed in winter when the gaseous phase is also considered with higher concentrations of NOPAHs at the traffic site, followed by the UB site and the lowest at the rural site (Tables S49–S50). However, in summer, for which data in the particulate phase were limited, when considering the total concentrations, the UB site had levels higher than those at the traffic site (i.e. T/UB < 1, Table S49) for all NOPAHs, except for 1-NPYR and 5-NACE. This could suggest secondary formation along transport from the traffic site to the UB site.

Concerning assessment of exposure, neglect of the gas-phase may cause significant underestimates (Samburova et al., 2017). This is suggested by identification of significant toxicities in the gas phase (e.g., androgenicity; Novak et al., 2014; Nováková et al., 2020). The bias is expected less pronounced for carcinogenicity, as equivalency factors are generally higher for the high-molecular weight i.e., the compounds being associated mostly with the particulate phase. Here, it was found that in winter, the particulate phase contributed about 50% of the toxicity due to NOPAHs, while it was only 7% in summer. When including also the PAHs in the ECR, the particulate phase contributed to 92% in winter and 64% in summer to the toxicity of all PACs, which is somewhat similar to those found by other studies (Huang et al., 2014a; Tomaz et al., 2016). In addition, the contribution of NOPAHs to the overall toxicity of PACs, which is rather small (i.e. 0–1.7%), is slightly higher when the gas phase is included, particularly in summer (Fig. S10). Overall, this suggests that gaseous NOPAHs contribute to some extent to the cancer risks due to exposure to PACs. Therefore, the gaseous phase should be considered for future health risk assessment.

## 4 Conclusions

This study provided novel information on levels of toxic organic chemicals in the atmosphere of two cities (Ljubljana and Brno) and a rural site in Central Europe, and assessed their seasonal and spatial variations as well as their gas-particle partitioning and their impact on human health via inhalation. Such type of data, so far rather limited for NOPAHs, are crucial to improve our understanding of their environmental fate, including their potential for air-surface exchange (hence, multi-compartmental cycling) and long-range transport.

This study helped to identify the multiple parameters influencing NOPAH levels such as the proximity to primary sources, the meteorological conditions or occurrence of other atmospheric pollutants. Moreover, the significance of secondary formation of some NOPAHs, further away from primary sources has been shown. In winter, the estimated cancer risks related to the inhalation of NOPAHs were above the acceptable lifetime carcinogenic risk for 27–47% of samples collected at all sites. This study underlines the importance of inclusion of the gas phase in addition to the particulate phase when assessing the atmospheric fate of polycyclic aromatic compounds and also when assessing the related health risk. Considering that information on toxicity was available only for a limited amount of NOPAHs and that this study targeted only a limited amount of all atmospherically relevant PACs, the true cancer risks are eventually significantly higher. Efforts to characterize human health risks through inhalation of both particulate and gaseous PACs, besides other organic contaminants, need to be pursued.

## CRediT authorship contribution statement

**Céline Degrendele:** Conceptualization, Investigation, Methodology, Writing – original draft. **Tjaša Kanduč:** Investigation, Methodology, Writing – review & editing. **David Kocman:** Investigation, Methodology, Writing – review & editing. **Gerhard Lammel:** Conceptualization, Investigation, Writing – review & editing. **Adriana Cambelová:** Investigation. **Saul Garcia Dos Santos:** Methodology, Writing – review & editing. **Milena Horvat:** Resources, Writing – review & editing. **Petr Kukučka:** Methodology, Writing – review & editing. **Adéla Holubová Šmejkalová:** Methodology, Writing – review & editing. **Ondřej Mikeš:** Methodology, Writing – review & editing. **Beatriz Nuñez-Corcuera:** Methodology. **Petra Přibylová:** Methodology, Writing – review & editing. **Roman Prokeš:** Methodology. **Ondřej Saňka:** Methodology, Writing – review & editing. **Thomas Maggos:** Conceptualization, Methodology, Resources, Writing – review & editing. **Denis Sarigiannis:** Conceptualization, Methodology, Resources. **Jana Klánová:** Methodology, Resources, Writing – review & editing.

## Declaration of competing interest

The authors declare that they have no conflict of interest.

## Appendix A Supplementary data

Description of samples collected, results from quality assurance and quality control, PEFs used, statistics about NOPAHs concentrations, winter-to-summer ratios, results of the Spearman and Pearson correlation analysis, cancer risks due to NOPAHs and PACs and particulate mass fractions of NOPAHs are provided. Image 1
