# Supplementary material for: NPAHs and OPAHs in the atmosphere of two central European cities: Seasonality, urban-to-background gradients, cancer risks and gas-to-particle partitioning
Source: Sci Total Environ. 2021 Nov 1;793:148528. doi: 10.1016/j.scitotenv.2021.148528 (PMC8434474; doi:10.1016/j.scitotenv.2021.148528)
Supplement: Supplementary file 1 — Description of samples collected, results from quality assurance and quality control, PEFs used, statistics about NOPAHs concentrations, winter-to-summer ratios, results of the Spearman and Pearson correlation analysis, cancer risks due to NOPAHs and PACs and particulate mass fractions of NOPAHs are provided. [file mmc1.docx]

**NPAHs and OPAHs in the atmosphere of two central European cities: seasonality, urban-to-background gradients, cancer risks and gas-to-particle partitioning**

**Supplementary information**

**Céline Degrendele^a*^, Tjaša Kanduč^b^, David Kocman^b^, Gerhard Lammel^a^, Adriana Cambelová^a^, Saul Garcia Dos Santos^c^, Milena Horvat^b^, Petr Kukučka^a^, Adéla Holubová Šmejkalová^d^, Ondřej Mikeš^a^, Beatriz Nuñez-Corcuera^c^, Petra Přibylová^a^, Roman Prokeš^a^, Ondřej Saňka^a^, Thomas Maggos^e^, Denis Sarigiannis^f,g,h^ and Jana Klánová^a^**

^a^RECETOX Centre, Masaryk University, Czech Republic

^b^Department of Environmental Sciences, Jožef Stefan Institute, Slovenia

**^c^**Área de Contaminación Atmosférica, Centro Nacional de Sanidad Ambiental Instituto de Salud Carlos III, Spain

^d^Czech Hydrometeorological Institute, Czech Republic

^e^Atmospheric Chemistry & Innovative Technologies Laboratory, NCSR "Demokritos", Greece

^f^Environmental Engineering Laboratory, Department of Chemical Engineering, Aristotle University of Thessaloniki, Thessaloniki, Greece

^g^HERACLES Research Centre on the Exposome and Health, Center for Interdisciplinary Research and Innovation, Thessaloniki, Greece

^h^University School of Advanced Study, Pavia, Italy

Corresponding author: celine.degrendele@recetox.muni.cz , RECETOX Centre, Masaryk University, Faculty of Sciences, A29, Kamenice 5, 62500 Brno, Czech Republic

Number of pages: 65

Number of Tables: 50

Number of Figures 10

TABLE OF CONTENTS

List of Tables

Table S1: Sampling dates and sampled volumes for each of the investigated site S4

Table S2: Average recoveries found in the samples S4

Table S3: Concentrations of individual NOPAHs measured in one filter divided onto four sub-samples S5

Table S4: Recoveries found on three spiked PUFs S6

Table S5: Statistics of the meteorological parameters and air pollutants available at each site in both campaigns S7

Table S6: Potency Equivalent Factors used S9

Table S7: Statistics of NPAHs levels in winter S10

Table S8: Statistics of NPAHs levels in summer S11

Table S9: Statistics of OPAHs levels in winter S12

Table S10: Statistics of OPAHs levels in summer S13

Table S11: Winter-to-summer ratios of average concentrations of NPAHs and OPAHs S14

Table S12: Traffic-to-urban background ratios of individual NOPAHs at Ljubljana S15

Table S13: Traffic-to-urban background ratios of individual NOPAHs at Brno S16

Table S14: Brno urban background-to-rural ratios of individual NOPAHs S17

Table S15: Results of the Spearman correlation analysis at the rural site in winter between the individual concentrations of NOPAHs and different meteorological parameters S18

Table S16: Results of the Spearman correlation analysis at the Brno urban background site in winter between the individual concentrations of NOPAHs and different meteorological parameters S19

Table S17: Results of the Spearman correlation analysis at the Brno traffic site in winter between the individual concentrations of NOPAHs and different meteorological parameters S20

Table S18: Results of the Spearman correlation analysis at the Ljubljana urban background site in winter between the individual concentrations of NOPAHs and different meteorological parameters S21

Table S19: Results of the Spearman correlation analysis at the Ljubljana traffic site in winter between the individual concentrations of NOPAHs and different meteorological parameters S22

Table S20: Results of the Spearman correlation analysis at the rural site in summer between the individual concentrations of NOPAHs and different meteorological parameters S23

Table S21: Results of the Spearman correlation analysis at the Brno urban background site in summer between the individual concentrations of NOPAHs and different meteorological parameters S24

Table S22: Results of the Spearman correlation analysis at the Brno traffic site in summer between the individual concentrations of NOPAHs and different meteorological parameters S25

Table S23: Results of the Spearman correlation analysis at the Ljubljana urban background site in summer between the individual concentrations of NOPAHs and different meteorological parameters S26

Table S24: Results of the Spearman correlation analysis at the Ljubljana traffic site in summer between the individual concentrations of NOPAHs and different meteorological parameters or air pollutants S27

Table S25: Pearson correlations between individual NOPAHs at the rural site in winter S28

Table S26: Pearson correlations between individual NOPAHs at the Brno urban background site in winter S29

Table S27: Pearson correlations between individual NOPAHs at the Brno traffic site in winter S30

Table S28: Pearson correlations between individual NOPAHs at the Ljubljana urban background site in winter S31

Table S29: Pearson correlations between individual NOPAHs at the Ljubljana traffic site in winter. S32

Table S30: Pearson correlations between individual NOPAHs at the Brno traffic site in summer. S33

Table S31: Pearson correlations between individual NOPAHs at the Ljubljana traffic site in summer S34

Table S32: Statistics of the ratios of individual NOPAHs and parent PAHs in winter S35

Table S33: Statistics of the ratios of individual NOPAHs and parent PAHs in summer S36

Table S34: Statistics of the cancer risks from particulate NOPAHs S37

Table S35: Statistics of the cancer risks from particulate NOPAHs and PAHs S37

Table S36: Statistics of NPAH levels in the gaseous phase S38

Table S37: Statistics of OPAH levels in the gaseous phase S39

Table S38: Statistics of NPAHs particulate mass fractions S40

Table S39: Statistics of OPAHs particulate mass fractions S41

Table 40: Gas and particle concentrations of NPAHs measured in individual samples at the rural site S42

Table 41: Gas and particle concentrations of OPAHs measured in individual samples at the rural site S43

Table 42: Gas and particle concentrations of NPAHs measured in individual samples at the urban background site S44

Table 43: Gas and particle concentrations of OPAHs measured in individual samples at the urban background site S45

Table 44: Gas and particle concentrations of NPAHs measured in individual samples at the traffic site S46

Table 45: Gas and particle concentrations of OPAHs measured in individual samples at the traffic site S47

Table S46: Comparison of the detection frequencies of the particulate phase and of the sum of gaseous and particulate phases of NPAHs S48

Table S47: Comparison of the detection frequencies of the particulate phase and of the sum of gaseous and particulate phases of OPAHs. S49

Table S48: Comparison of the winter-to summer ratios of the average concentrations when considering only the particulate or the total concentrations. S50

Table S49: Traffic to urban background ratios of NPAHs and OPAHs based on total concentrations S51

Table S50: Urban background to rural ratios of NPAHs and OPAHs based on total concentrations S52

List of Figures

Figure S1: Detection frequencies of individual NPAHs in winter and summer S53

Figure S2: Detection frequencies of individual OPAHs in winter and summer S54

Figure S3: Temporal variations of the NPAHs composition profile. S56

Figure S4: Temporal variations of the OPAHs composition profile. S58

Figure S5: Caracterisation of the vehicle technologies among personal cars in Ljubljana and Brno S59

Figure S6: Relationships between Σ_9_NPAHs and Σ_11_OPAHs with temperature, PM_2.5_, OC, EC, O_3_ and NO_2_ at the Brno UB site in winter S60

Figure S7: Ratios between individual NOPAHs and their parent PAHs. S61

Figure S8: Boxplots of ECR related to particulate NOPAHs S62

Figure S9: Comparison of the composition profile when considering only the particulate or the total concentrations S63

Figure S10: Comparison of the cancer risks due to NOPAHs when the particulate or total concentrations are considered and contribution of NOPAHs to the cancer risks due to PACs S64

Table S1: Sampling dates and sampled volumes for each of the investigated site

| City | Site | Coordonates | Season | Sampling start | Sampling end | Sampled volume (min-max, m3) | Samples collected (particle/gas) |
| --- | --- | --- | --- | --- | --- | --- | --- |
| R (NAOK) | | 49°34´24.221"N;15° 4´ 49.002"E | Winter | 13/02/17 | 20/02/17 | 770-787 | 8/8 |
|  |  |  | Summer | 6/7/2017 | 13/07/17 | 793-829 | 8/8 |
| Brno | T (Kotlařská) | 49° 12' 19.79"N; 16° 35' 49.9"E | Winter | 3/2/2017 | 7/3/2017 | 752-783 | 30/7 |
|  |  |  | Summer | 12/6/2017 | 13/07/17 | 755-809 | 30/8 |
|  | UB (Lany) | 49° 9´54.937"N;16°34´50.925"E | Winter | 3/2/2017 | 7/3/2017 | 55 | 30/7 |
|  |  |  | Summer | 12/6/2017 | 13/07/17 | 55 | 30/8 |
| Ljubljana | T (MOL- Center) | 46°03′24.1”N | Winter | 20/02/17 | 21/03/17 | 53-55 | 30/0 |
|  |  | 14°30′10.8″E | Summer | 15/05/17 | 13/06/17 | 53-55 | 26/0 |
|  | UB (SEA- Bežigrad) | 46°03′55.6”N | Winter | 22/02/17 | 25/03/17 | 54-55 | 30/0 |
|  |  | 14°30′44.0″E | Summer | 15/05/17 | 13/06/17 | 38 | 30/0 |

Table S2: Average recoveries (in %) found in the samples

|  | Min | Max | Average | SD | Median |
| --- | --- | --- | --- | --- | --- |
| 1-nitronaphthalene-D7 | 12 | 203 | 83 | 26 | 81 |
| 2-nitrofluorene-D9 | 17 | 243 | 111 | 43 | 99 |
| 9-nitroanthracene-D9 | 19 | 209 | 98 | 34 | 94 |
| 3-nitrofluoranthene-D9 | 11 | 280 | 104 | 44 | 95 |
| 1-nitropyrene-D9 | 22 | 367 | 102 | 46 | 93 |
| 6-nitrochrysene-D11 | 7 | 329 | 102 | 51 | 95 |

Table S3: Concentrations (in pg m^-3^) of individual NOPAHs measured in one filter divided onto four sub-samples. <iLOQ indicates when the level was smaller than the instrumental limit of quantification. The remaining NOPAHs were not quantified in these samples

| **NPAHs** | Sample 1 | Sample 2 | Sample 3 | Sample 4 |
| --- | --- | --- | --- | --- |
| 1-NNAP | 1.84 | 1.68 | 1.79 | 2.43 |
| 9-NANT | 256.37 | 253.32 | 236.09 | 250.94 |
| 3-NPHE | 9.30 | 10.72 | 11.52 | 10.84 |
| 2-NFLT | 95.45 | 114.30 | 115.01 | 103.75 |
| **OPAHs** |  |  |  |  |
| 1,4-O_2_NAP | 0.13 | 0.12 | 0.13 | 0.14 |
| 1(CHO)NAP | 0.071 | 0.063 | 0.072 | 0.072 |
| 9-OFLN | 0.19 | 0.19 | 0.18 | 0.19 |
| 9,10- O_2_ANT | 2.07 | 1.90 | 1.84 | 2.00 |
| BaOFLN | 0.51 | 0.52 | 0.53 | 0.49 |
| BbOFLN | <iLOQ | 0.083 | 0.065 | 0.076 |
| BAN | 1.89 | 1.99 | 2.19 | 2.02 |
| 7,12-O_2_BAA | 0.24 | 0.29 | 0.31 | 0.28 |
| 5,12-O_2_NAC | 0.068 | 0.076 | 0.094 | 0.080 |

Table S4: Recoveries (in %) found on three spiked PUFs

| **NPAHs** | Sample 1 | Sample 2 | Sample 3 |
| --- | --- | --- | --- |
| 1-NNAP | 46 | 47 | 67 |
| 2-NNAP | 61 | 51 | 68 |
| 3-NACE | 110 | 112 | 127 |
| 5-NACE | 116 | 105 | 114 |
| 2-NFLN | 102 | 91 | 114 |
| 9-NANT | 100 | 87 | 95 |
| 9-NPHE | 106 | 94 | 109 |
| 3-NPHE | 125 | 110 | 120 |
| 2-NFLT | 83 | 72 | 90 |
| 3-NFLT | 114 | 93 | 119 |
| 1-NPYR | 82 | 71 | 95 |
| 2-NPYR | 64 | 56 | 73 |
| 7-NBAA | 85 | 77 | 88 |
| 6-NCHR | 108 | 121 | 149 |
| **OPAHs** |  |  |  |
| 1,4-O2NAP | 138 | 135 | 126 |
| 1(CHO)NAP | 126 | 109 | 111 |
| 9-OFLN | 87 | 78 | 100 |
| 9,10-O2ANT | 116 | 103 | 95 |
| 1,4-O2ANT | 114 | 96 | 90 |
| 9,10-O2PHE | 53 | 56 | 70 |
| BaOFLN | 108 | 96 | 121 |
| BbOFLN | 109 | 94 | 109 |
| BAN | 112 | 95 | 114 |
| 7,12-O2BAA | 107 | 98 | 112 |
| 5,12-O2NAC | 112 | 99 | 99 |

Table S5: Statistics of the meteorological parameters (WD = wind direction, WS = wind speed, RH = relative humidity, T = temperature) and air pollutants available at each site in both campaigns. NA indicates cases when the parameter was not available

|  | | Winter | | | | | Summer | | | | |
| --- | --- | --- | --- | --- | --- | --- | --- | --- | --- | --- | --- |
|  |  | Brno | | | Ljubljana | | Brno | | | Ljubljana | |
|  | Units | R | UB | T | UB | T | R | UB | T | UB | T |
| **WD** | ° | 210±50  (115-264) | 193±58  (37-279) | NA | NA | NA | 224±36 (151-267) | NA | NA | NA | NA |
| **WS** | m s^-1^ | 2.2±1.5  (0.8-5.4) | 2.6±1.3  (1.1-6.6) | NA | 1.9±0.8 (0.8-4) | 0.9±0.3 (0.4-1.8) | 2.4±0.8 (1.4-4) | NA | NA | 1.6±0.5 (1.1-3) | 0.7±0.2 (0.2-1.1) |
| **RH** | % | 72±13  (55-89) | 78±12  (56-98) | NA | 64±12  (36-84) | 59±11  (33-81) | 67±6  (58-78) | NA | NA | 60±9  (52-84) | 57±9  (45-79) |
| **T** | °C | 1.4±1.9  (-1.6-3.8) | 2.8±4  (-4-12.5) | NA | 9.2±2.2 (4.9-13.9) | 8.1±2.2  (3-12.1) | 17.7±3.5 (11.6-21.4) | NA | NA | 20.5±2.9 (12.6-23.9) | 19.2±2.9 (12.6-24.3) |
| **SO_2_** | µg m^-3^ | 2.2±2.2  (0.5-7.2) | 5.1±4  (1.4-19.7) | NA | 6.9±3.1  (1-13.4) | 2.3±0.6 (1.3-3.2) | 2.1±0.4 (1.7-2.6) | 3.1±1.4  (1.4-5.2) | NA | 6.7±2.8 (2.2-10.8) | 2.4±1.8  (0-7.8) |
| **PM_2.5_** | µg m^-3^ | 17.7±8.9 (8.9-33.9) | 29.7±24.1  (5.3-111.1) | 32.9±19.9 (13.5-93) | 31.4±24.1 (5.5-116.2) | 28.7±17.5 (4.7-64) | 6.8±2.9 (3.5-10.8) | 11.9±3  (5.8-18.5) | 14.9±3.8 (9.1-26.1) | 24.7±4.3 (15.4-31.7) | 25.1±4.4 (15.7-34.5) |
| **PM_10_** | µg m^-3^ | 30.7±19  (11-65.3) | 43.8±31.5  (7.8-126.5) | NA | NA | NA | 15.6±4.9 (7.3-21.2) | 13.9±6  (4.4-28.2) | NA | NA | NA |
| **O_3_** | µg m^-3^ | 68.9±14.6 (49.3-89.3) | 32.8±20.7  (5.6-91.5) | NA | 54±19.8 (28-96.9) | NA | 77.9±15.4 (54.8-102) | 77.4±19.8 (53.2-120.4) | NA | 77.2±20.2 (40.2-110.2) | NA |
| **NO_x_** | µg m^-3^ | 12.6±3.2 (9.6-17.4) | 101.3±66.8 (14.5-261.6) | NA | NA | NA | 4.6±0.8 (3.2-5.7) | 26.3±15.1 (2.6-56.9) | NA | NA | NA |
| **NO_2_** | µg m^-3^ | 11.7±2.6  (9-15.4) | 43.6±19  (11.5-90.2) | NA | 35.5±15 (14-61.7) | 54.4±13.9 (29.6-87.9) | 4±0.8  (2.6-5.2) | 18.1±7.4 (4.5-32.7) | NA | 19.9±4.2 (10.1-26.8) | 46.9±6.9 (35.8-59.7) |
| **NO** | µg m^-3^ | 0.6±0.4  (0.3-1.3) | 37.7±32.6  (2.1-114.5) | NA | NA | NA | 0.3±0.1 (0.2-0.5) | 5.7±5.1  (0.6-15.8) | NA | NA | NA |
| **CO** | µg m^-3^ | 346.9±98.8 (227.2-507.5) | 545.9±210.9 (218.8-1052.1) | NA | 0.2±0.1 (0.1-0.6) | NA | 208.6±33 (159.1-252.5) | 217.2±48.7 (119-325) | NA | 0.1±0.1 (0.1-0.2) | NA |
| **PM_1_** | µg m^-3^ | NA | 33±24.7  (5.1-103.3) | NA | NA | NA | NA | NA | NA | NA | NA |
| **Cu** | ng m^-3^ | 2.3±0.8  (1.8-2.9) | 9.8±5.7  (1.3-24.1) | 8±5.6  (1-26.1) | 41.3±60.4 (3.7-244.7) | 19.4±15.2 (4.8-62.3) | 6.5±0.1 (6.5-6.6) | 5.3±1.9  (1.8-8) | 9.5±5 (0.4-22.3) | 16.7±17.1 (3-74.4) | 12.9±11.8 (0-41.6) |
| **Zn** | ng m^-3^ | 14.5±8.6 (3.9-27.9) | 42.7±28.9  (6.3-128) | 34.2±24.3 (7.6-109.6) | 40±44.2 (3.4-190.7) | 25.7±26 (1.4-125.7) | 5±3.1  (1.1-9.5) | 8.6±4.1  (1.5-17.6) | 18.3±6.8 (2.9-35.1) | 25.7±47.5 (3.6-245) | 12.9±8.9 (0-30.2) |
| **OC** | µg m^-3^ | 4.7±2.6  (1.8-9.2) | 10.6±7.8  (2.3-36.3) | 8.5±5.6  (3-27.2) | 7.4±4.1 (1.7-17.7) | 6.3±3.4  (0-14.2) | 2.1±0.8 (0.9-3.1) | 3.4±0.8  (2-5.2) | 3.6±0.8 (2.1-5.6) | 3.8±0.9 (1.6-5) | NA |
| **EC** | µg m^-3^ | 0.6±0.3  (0.3-1.1) | 1.3±0.6  (0.4-2.8) | 2.3±0.6 (0.9-3.8) | 1.3±0.7 (0.4-3.4) | 1.5±0.8 (0.4-3.5) | 0.2±0.1 (0.1-0.3) | 0.6±0.2  (0.2-0.9) | 1.7±0.4 (0.9-2.3) | 0.7±0.2 (0.3-1.2) | 1.1±0.3 (0.6-1.8) |

Table S6: Potency Equivalent Factors (PEFs) used

|  | Compound | PEF | Reference |
| --- | --- | --- | --- |
| PAHs | Naphthalene | 0.001 | (Nisbet and LaGoy, 1992) |
|  | Acenaphthylene | 0.00056 | (Durant et al., 1996) |
|  | Acenaphthene | 0.001 | (Nisbet and LaGoy, 1992) |
|  | Fluorene | 0.001 | (Nisbet and LaGoy, 1992) |
|  | Phenanthrene | 0.0 | (USEPA, 2010) |
|  | Anthracene | 0.0 | (USEPA, 2010) |
|  | Fluoranthene | 0.08 | (USEPA, 2010) |
|  | Pyrene | 0.0 | (USEPA, 2010) |
|  | Benozo(a)anthracene | 0.2 | (USEPA, 2010) |
|  | Chrysene | 0.1 | (USEPA, 2010) |
|  | Benzo(b)fluoranthene | 0.8 | (USEPA, 2010) |
|  | Benzo(k)fluoranthene | 0.03 | (USEPA, 2010) |
|  | Benozo(a)pyrene | 1 | (OEHHA, 1994) |
|  | Benzo(g,h,i)perylene | 0.009 | (USEPA, 2010) |
|  | Indeno(1,2,3-cd)pyrene | 0.1 | (OEHHA, 1994) |
|  | Dibenz(a,h)anthracene | 10 | (USEPA, 2010) |
| NPAHs | 1-NPYR | 0.1 | (OEHHA, 1994) |
|  | 5-NACE | 0.01 | (OEHHA, 1994) |
|  | 2-NFLN | 0.01 | (OEHHA, 1994) |
|  | 9-NANT | 0.0032 | (Durant et al., 1996) |
|  | 2-NFLT | 0.05 | (Durant et al., 1996) |
|  | 3-NFLT | 0.0026 | (Durant et al., 1996) |
|  | 1-NPYR | 0.1 | (OEHHA, 1994) |
|  | 6-NCHR | 10 | (OEHHA, 1994) |
|  | 1,3-N_2_PYR | 0.031 | (Durant et al., 1996) |
|  | 1,6-N_2_PYR | 10 | (OEHHA, 1994) |
|  | 1,8-N_2_PYR | 1 | (OEHHA, 1994) |
| OPAHs | 9,10-O_2_ANT | 0.018 | (Durant et al., 1996) |
|  | BAN | 0.0039 | (Durant et al., 1996) |

Table S7: Statistics (detection frequency (DF, in %), minimum, maximum, average and standard deviation (SD)) of NPAHs levels (pg m^-3^) in winter. <LOQ means smaller than the limits of quantification

|  | | | 1-NNAP | 2-NNAP | 2-NFLN | 9-NANT | 3-NPHE | 2-NFLT | 3-NFLT | 1-NPYR | 2-NPYR | 7-NBAA | 6-NCHR | 1,3-N2PYR | 1,6-N2PYR | **Σ9NPAHs** |
| --- | --- | --- | --- | --- | --- | --- | --- | --- | --- | --- | --- | --- | --- | --- | --- | --- |
| **R** | | **DF** | 50 | 0 | 0 | 100 | 50 | 100 | 88 | 88 | 100 | 88 | 25 | 0 | 0 | 100 |
|  |  | **Min** | 0.10 |  |  | 0.94 | 0.87 | 1.54 | 0.61 | 0.31 | 0.28 | 1.71 | 0.38 |  |  | 2.77 |
|  |  | **Max** | 0.21 |  |  | 144.94 | 2.60 | 50.37 | 6.95 | 4.67 | 3.80 | 17.44 | 0.65 |  |  | 225.15 |
|  |  | **Average** | 0.15 |  |  | 41.56 | 1.58 | 24.19 | 3.11 | 1.78 | 1.89 | 7.55 | 0.52 |  |  | 79.39 |
|  |  | **SD** | 0.06 |  |  | 47.42 | 0.76 | 19.25 | 2.22 | 1.62 | 1.29 | 5.06 | 0.19 |  |  | 73.50 |
| **Brno** | **UB** | **DF** | 27 | 0 | 0 | 73 | 0 | 100 | 37 | 100 | 100 | 37 | 0 | 0 | 0 | 100 |
|  |  | **Min** | 0.33 |  |  | 5.58 | 0.00 | 2.30 | 5.54 | 0.25 | 0.54 | 5.76 |  |  |  | 3.09 |
|  |  | **Max** | 0.65 |  |  | 477.37 | 0.00 | 367.22 | 65.99 | 81.65 | 104.37 | 134.88 |  |  |  | 1113.37 |
|  |  | **Average** | 0.44 |  |  | 72.49 | 0.00 | 63.62 | 24.86 | 12.09 | 18.14 | 45.85 |  |  |  | 173.06 |
|  |  | **SD** | 0.10 |  |  | 127.06 | 0.00 | 88.07 | 18.35 | 20.98 | 26.51 | 37.21 |  |  |  | 289.18 |
|  | **T** | **DF** | 100 | 100 | 7 | 100 | 100 | 100 | 73 | 100 | 100 | 73 | 0 | 23 | 3 | 100 |
|  |  | **Min** | 0.19 | 0.04 | 0.09 | 2.46 | 0.63 | 2.38 | 0.52 | 1.01 | 0.36 | 1.67 |  | 0.02 | 0.16 | 9.30 |
|  |  | **Max** | 6.06 | 3.13 | 0.41 | 1709.66 | 46.01 | 841.18 | 131.08 | 192.40 | 187.06 | 442.58 |  | 0.09 | 0.16 | 3007.61 |
|  |  | **Average** | 1.62 | 0.66 | 0.25 | 317.63 | 10.34 | 122.25 | 26.81 | 30.83 | 32.39 | 79.26 |  | 0.06 | 0.16 | 593.49 |
|  |  | **SD** | 1.57 | 0.72 | 0.23 | 495.31 | 12.77 | 187.12 | 33.04 | 42.79 | 47.72 | 106.90 |  | 0.02 | 0.00 | 867.48 |
| **Ljubljana** | **UB** | **DF** | 27 | 7 | 0 | 67 | 17 | 100 | 67 | 73 | 80 | 57 | 0 | 0 | 0 | 100 |
|  |  | **Min** | 0.73 | 0.59 |  | 4.00 | 6.23 | 3.23 | 1.21 | 0.81 | 1.03 | 2.60 |  |  |  | 3.23 |
|  |  | **Max** | 1.52 | 0.62 |  | 197.23 | 10.77 | 94.87 | 13.61 | 9.97 | 53.59 | 87.09 |  |  |  | 365.46 |
|  |  | **Average** | 1.09 | 0.60 |  | 69.09 | 8.66 | 30.71 | 5.91 | 4.68 | 19.27 | 29.73 |  |  |  | 118.18 |
|  |  | **SD** | 0.28 | 0.03 |  | 62.10 | 2.08 | 27.05 | 4.04 | 2.81 | 16.83 | 27.18 |  |  |  | 122.42 |
|  | **T** | **DF** | 43 | 0 | 0 | 57 | 7 | 100 | 60 | 80 | 90 | 53 | 0 | 0 | 0 | 100 |
|  |  | **Min** | 0.72 |  |  | 3.98 | 7.56 | 2.03 | 1.34 | 1.12 | 0.83 | 0.76 |  |  |  | 2.03 |
|  |  | **Max** | 1.66 |  |  | 189.22 | 8.73 | 70.41 | 12.41 | 11.44 | 44.03 | 58.68 |  |  |  | 301.88 |
|  |  | **Average** | 1.08 |  |  | 54.74 | 8.15 | 22.36 | 4.85 | 4.80 | 13.30 | 19.75 |  |  |  | 83.65 |
|  |  | **SD** | 0.22 |  |  | 54.22 | 0.83 | 18.61 | 3.08 | 2.67 | 12.40 | 19.92 |  |  |  | 90.38 |

Table S8: Statistics (detection frequency (DF, in %), minimum, maximum, average and standard deviation (SD)) of NPAHs levels (pg m^-3^) in summer. <LOQ means smaller than the limits of quantification

|  | | | 1-NNAP | 2-NNAP | 2-NFLN | 9-NANT | 3-NPHE | 2-NFLT | 3-NFLT | 1-NPYR | 2-NPYR | 7-NBAA | 6-NCHR | 1,3-N2PYR | 1,6-N2PYR | **Σ9NPAHs** |
| --- | --- | --- | --- | --- | --- | --- | --- | --- | --- | --- | --- | --- | --- | --- | --- | --- |
| **R** | | **DF** | 0 | 0 | 0 | 0 | 0 | 0 | 0 | 0 | 0 | 0 | 0 | 0 | 0 | 0 |
|  |  | **Min** |  |  |  |  |  |  |  |  |  |  |  |  |  |  |
|  |  | **Max** |  |  |  |  |  |  |  |  |  |  |  |  |  |  |
|  |  | **Average** |  |  |  |  |  |  |  |  |  |  |  |  |  |  |
|  |  | **SD** |  |  |  |  |  |  |  |  |  |  |  |  |  |  |
| **Brno** | **UB** | **DF** | 0 | 27 | 0 | 0 | 0 | 0 | 0 | 0 | 0 | 0 | 0 | 0 | 0 | 27 |
|  |  | **Min** |  | 3.90 |  |  |  |  |  |  |  |  |  |  |  | <LOQ |
|  |  | **Max** |  | 11.06 |  |  |  |  |  |  |  |  |  |  |  | 11.06 |
|  |  | **Average** |  | 6.01 |  |  |  |  |  |  |  |  |  |  |  | 1.60 |
|  |  | **SD** |  | 2.28 |  |  |  |  |  |  |  |  |  |  |  | 2.93 |
|  | **T** | **DF** | 0 | 70 | 0 | 0 | 100 | 100 | 97 | 100 | 100 | 87 | 0 | 0 | 0 | 100 |
|  |  | **Min** |  | 0.12 |  |  | 0.21 | 0.29 | 0.15 | 0.43 | 0.07 | 0.07 |  |  |  | 1.70 |
|  |  | **Max** |  | 0.37 |  |  | 1.53 | 7.60 | 1.34 | 3.45 | 0.55 | 2.21 |  |  |  | 15.73 |
|  |  | **Average** |  | 0.22 |  |  | 0.78 | 1.55 | 0.53 | 1.72 | 0.22 | 0.64 |  |  |  | 5.48 |
|  |  | **SD** |  | 0.06 |  |  | 0.36 | 1.44 | 0.32 | 0.72 | 0.13 | 0.59 |  |  |  | 3.03 |
| **Ljubljana** | **UB** | **DF** | 0 | 0 | 0 | 0 | 0 | 0 | 7 | 30 | 0 | 0 | 0 | 0 | 0 | 33 |
|  |  | **Min** |  |  |  |  |  |  | 0.37 | 0.86 |  |  |  |  |  | <LOQ |
|  |  | **Max** |  |  |  |  |  |  | 0.47 | 1.64 |  |  |  |  |  | 1.64 |
|  |  | **Average** |  |  |  |  |  |  | 0.42 | 1.11 |  |  |  |  |  | 0.36 |
|  |  | **SD** |  |  |  |  |  |  | 0.07 | 0.26 |  |  |  |  |  | 0.56 |
|  | **T** | **DF** | 0 | 8 | 0 | 23 | 81 | 100 | 88 | 100 | 92 | 50 | 0 | 0 | 0 | 100 |
|  |  | **Min** |  | 2.62 |  | 4.34 | <0.01 | 0.01 | <0.01 | <0.01 | <0.01 | 1.99 |  |  |  | 0.01 |
|  |  | **Max** |  | 2.87 |  | 28.93 | 4.34 | 18.59 | 3.29 | 4.32 | 5.91 | 28.71 |  |  |  | 93.55 |
|  |  | **Average** |  | 2.75 |  | 12.22 | 2.85 | 9.02 | 1.85 | 2.40 | 1.31 | 10.82 |  |  |  | 25.00 |
|  |  | **SD** |  | 0.18 |  | 8.85 | 0.93 | 4.24 | 0.70 | 0.97 | 1.20 | 8.98 |  |  |  | 18.57 |

Table S9: Statistics (detection frequency (DF, in %), minimum, maximum, average and standard deviation (SD)) of OPAHs levels (pg m^-3^) in winter. <LOQ means smaller than the limits of quantification

|  | | | 1,4-O2NAP | 1(CHO)NAP | 9-OFLN | 9,10-O2ANT | 1,4-O2ANT | 9,10-O2PHE | BaOFLN | BbOFLN | BAN | 7,12-O2BAA | 5,12-O2NAC | **Σ11OPAHs** |
| --- | --- | --- | --- | --- | --- | --- | --- | --- | --- | --- | --- | --- | --- | --- |
| **R** | | **DF** | 50 | 38 | 25 | 100 | 75 | 88 | 100 | 100 | 100 | 100 | 100 | 100 |
|  |  | **Min** | 4.94 | 5.09 | 60.07 | 30.68 | 0.62 | 47.75 | 6.38 | 9.07 | 20.32 | 5.79 | 1.46 | 73.70 |
|  |  | **Max** | 11.41 | 5.97 | 93.24 | 514.47 | 4.77 | 575.66 | 163.50 | 192.03 | 242.00 | 75.72 | 30.46 | 1721.28 |
|  |  | **Average** | 8.32 | 5.64 | 76.66 | 302.52 | 2.98 | 245.69 | 66.89 | 97.13 | 153.27 | 39.94 | 12.71 | 915.12 |
|  |  | **SD** | 2.68 | 0.48 | 23.46 | 152.01 | 1.66 | 178.06 | 55.17 | 72.40 | 83.78 | 25.78 | 9.66 | 589.35 |
| **Brno** | **UB** | **DF** | 37 | 23 | 7 | 47 | 0 | 0 | 100 | 90 | 100 | 97 | 100 | 100 |
|  |  | **Min** | 86.13 | 47.19 | 1051.77 | 133.79 |  |  | 4.80 | 17.60 | 23.42 | 6.81 | 1.43 | 29.64 |
|  |  | **Max** | 664.77 | 175.04 | 1235.32 | 9714.07 |  |  | 4024.66 | 6852.46 | 8046.21 | 1360.32 | 1119.45 | 31912.60 |
|  |  | **Average** | 282.37 | 87.40 | 1143.54 | 2007.82 |  |  | 476.23 | 895.53 | 1212.62 | 217.08 | 134.55 | 3976.38 |
|  |  | **SD** | 176.28 | 49.88 | 129.79 | 2981.79 |  |  | 977.14 | 1715.95 | 2063.39 | 354.30 | 273.55 | 7961.60 |
|  | **T** | **DF** | 50 | 63 | 37 | 100 | 50 | 63 | 100 | 100 | 100 | 100 | 100 | 100 |
|  |  | **Min** | 6.10 | 3.18 | 75.40 | 81.50 | 0.55 | 21.19 | 17.83 | 18.61 | 26.17 | 5.78 | 1.62 | 178.35 |
|  |  | **Max** | 529.63 | 195.61 | 915.15 | 5220.60 | 4.16 | 419.25 | 3538.81 | 4239.00 | 3734.73 | 1465.62 | 1512.84 | 20858.69 |
|  |  | **Average** | 144.01 | 49.32 | 387.95 | 1105.33 | 2.07 | 155.89 | 664.39 | 779.61 | 918.19 | 305.90 | 211.49 | 4330.17 |
|  |  | **SD** | 147.40 | 56.91 | 283.45 | 1428.31 | 1.21 | 119.99 | 1002.91 | 1145.25 | 1162.46 | 438.39 | 382.08 | 5787.86 |
| **Ljubljana** | **UB** | **DF** | 67 | 70 | 63 | 43 | 0 | 0 | 50 | 27 | 100 | 87 | 90 | 100 |
|  |  | **Min** | 51.32 | 16.12 | 37.14 | 208.92 |  |  | 67.90 | 103.92 | 108.00 | 54.03 | 4.11 | 122.99 |
|  |  | **Max** | 173.84 | 46.81 | 96.98 | 775.34 |  |  | 383.70 | 606.29 | 2385.20 | 317.71 | 101.66 | 4228.33 |
|  |  | **Average** | 112.08 | 32.43 | 60.84 | 515.01 |  |  | 190.13 | 374.26 | 783.09 | 153.39 | 36.00 | 1502.41 |
|  |  | **SD** | 36.34 | 8.68 | 18.57 | 175.60 |  |  | 90.67 | 235.45 | 635.72 | 93.81 | 29.60 | 1320.83 |
|  | **T** | **DF** | 60 | 60 | 50 | 47 | 0 | 0 | 63 | 33 | 97 | 83 | 93 | 100 |
|  |  | **Min** | 55.25 | 17.98 | 37.69 | 184.53 |  |  | 60.15 | 84.14 | 120.97 | 44.03 | 3.40 | 78.91 |
|  |  | **Max** | 157.06 | 39.90 | 110.02 | 727.19 |  |  | 342.88 | 588.14 | 2023.55 | 322.23 | 89.95 | 4011.60 |
|  |  | **Average** | 106.77 | 30.43 | 60.40 | 409.40 |  |  | 146.35 | 280.93 | 685.41 | 129.91 | 28.77 | 1287.57 |
|  |  | **SD** | 28.42 | 6.21 | 20.90 | 193.80 |  |  | 82.50 | 169.54 | 560.28 | 79.97 | 25.38 | 1133.48 |

Table S10: Statistics (detection frequency (DF, in %), minimum, maximum, average and standard deviation (SD)) of OPAHs levels (pg m^-3^) in summer. <LOQ means smaller than the limits of quantification

|  | | | 1,4-O2NAP | 1(CHO)NAP | 9-OFLN | 9,10-O2ANT | 1,4-O2ANT | 9,10-O2PHE | BaOFLN | BbOFLN | BAN | 7,12-O2BAA | 5,12-O2NAC | **Σ11OPAHs** |
| --- | --- | --- | --- | --- | --- | --- | --- | --- | --- | --- | --- | --- | --- | --- |
| **R** | | **DF** | 88 | 63 | 0 | 0 | 0 | 0 | 63 | 88 | 100 | 13 | 100 | 100 |
|  |  | **Min** | 3.22 | 1.21 |  |  |  |  | 0.34 | 0.62 | 0.99 | 2.21 | 0.07 | 1.69 |
|  |  | **Max** | 9.58 | 2.62 |  |  |  |  | 1.05 | 2.43 | 7.04 | 2.21 | 0.30 | 16.74 |
|  |  | **Average** | 4.74 | 1.52 |  |  |  |  | 0.65 | 1.13 | 2.42 | 2.21 | 0.15 | 9.34 |
|  |  | **SD** | 2.20 | 0.62 |  |  |  |  | 0.27 | 0.68 | 1.96 | 0.00 | 0.07 | 4.75 |
| **Brno** | **UB** | **DF** | 0 | 0 | 0 | 0 | 0 | 0 | 7 | 3 | 3 | 3 | 0 | 7 |
|  |  | **Min** |  |  |  |  |  |  | 5.07 | 8.43 | 16.15 | 11.49 |  | <LOQ |
|  |  | **Max** |  |  |  |  |  |  | 8.29 | 8.43 | 16.15 | 11.49 |  | 44.36 |
|  |  | **Average** |  |  |  |  |  |  | 6.68 | 8.43 | 16.15 | 11.49 |  | 1.65 |
|  |  | **SD** |  |  |  |  |  |  | 2.28 |  |  |  |  | 8.12 |
|  | **T** | **DF** | 90 | 50 | 0 | 67 | 0 | 70 | 100 | 100 | 100 | 100 | 0 | 100 |
|  |  | **Min** | 3.64 | 1.25 |  | 5.51 |  | 5.79 | 3.28 | 2.42 | 4.19 | 1.85 |  | 13.44 |
|  |  | **Max** | 14.24 | 3.10 |  | 16.47 |  | 25.65 | 20.74 | 17.40 | 21.49 | 9.83 |  | 105.40 |
|  |  | **Average** | 7.40 | 2.17 |  | 9.67 |  | 12.41 | 8.44 | 6.42 | 9.47 | 5.30 |  | 52.51 |
|  |  | **SD** | 2.91 | 0.50 |  | 2.82 |  | 4.95 | 4.21 | 3.69 | 4.36 | 2.38 |  | 25.16 |
| **Ljubljana** | **UB** | **DF** | 13 | 0 | 0 | 0 | 0 | 7 | 0 | 0 | 0 | 0 | 0 | 20 |
|  |  | **Min** | 113.16 |  |  |  |  | 45.98 |  |  |  |  |  | 0.00 |
|  |  | **Max** | 150.24 |  |  |  |  | 53.81 |  |  |  |  |  | 150.24 |
|  |  | **Average** | 124.91 |  |  |  |  | 49.90 |  |  |  |  |  | 19.98 |
|  |  | **SD** | 17.43 |  |  |  |  | 5.54 |  |  |  |  |  | 44.08 |
|  | **T** | **DF** | 0 | 15 | 8 | 0 | 0 | 0 | 8 | 4 | 12 | 4 | 31 | 35 |
|  |  | **Min** |  | 42.72 | 155.58 |  |  |  | 93.13 | 278.72 | 213.29 | 221.39 | 8.79 | <LOQ |
|  |  | **Max** |  | 51.03 | 170.36 |  |  |  | 121.32 | 278.72 | 791.14 | 221.39 | 85.20 | 1497.78 |
|  |  | **Average** |  | 46.15 | 162.97 |  |  |  | 107.23 | 278.72 | 423.74 | 221.39 | 21.06 | 102.49 |
|  |  | **SD** |  | 3.64 | 10.45 |  |  |  | 19.94 |  | 319.30 |  | 26.01 | 302.18 |

Table S11: Winter-to-summer ratios of average concentrations of NPAHs (a) and OPAHs (b)

| a) | R | Brno | | Ljubljana | |
| --- | --- | --- | --- | --- | --- |
|  |  | UB | T | UB | T |
| 1-NNAP |  |  |  |  |  |
| 2-NNAP |  |  | 3.05 |  |  |
| 9-NANT |  |  |  |  | 4.48 |
| 3-NPHE |  |  | 13.18 |  | 2.86 |
| 2-NFLT |  |  | 79.09 |  | 2.48 |
| 3-NFLT |  |  | 50.57 | 14.15 | 2.63 |
| 1-NPYR |  |  | 17.95 | 4.24 | 2.00 |
| 2-NPYR |  |  | 148.96 |  | 10.17 |
| 7-NBAA |  |  | 124.16 |  | 1.83 |

| b) | R | Brno | | Ljubljana | |
| --- | --- | --- | --- | --- | --- |
|  |  | UB | T | UB | T |
| 1,4-O2NAP | 1.75 |  | 19.46 | 0.90 |  |
| 1(CHO)NAP | 3.72 |  | 22.69 |  | 0.66 |
| 9-OFLN |  |  |  |  | 0.37 |
| 9,10-O2ANT |  |  | 114.33 |  |  |
| 1,4-O2ANT |  |  |  |  |  |
| 9,10-O2PHE |  |  | 12.56 |  |  |
| BaOFLN | 103.61 | 71.30 | 78.73 |  | 1.36 |
| BbOFLN | 85.64 | 106.28 | 121.35 |  | 1.01 |
| BAN | 63.34 | 75.09 | 96.93 |  | 1.62 |
| 7,12-O2BAA | 18.10 | 18.89 | 57.76 |  | 0.59 |
| 5,12-O2NAC | 82.24 |  |  |  | 1.37 |

Table S12: Traffic-to-urban background ratios of individual NOPAHs at Ljubljana. Only the samples collected simultanouesly were considered

|  | Winter | | | | | | Summer | | | | | |
| --- | --- | --- | --- | --- | --- | --- | --- | --- | --- | --- | --- | --- |
|  | N | Min | Max | Average | Median | SD | N | Min | Max | Average | Median | SD |
| 1-NNAP | 5 | 0.76 | 2.16 | 1.15 | 0.92 | 0.58 | 0 |  |  |  |  |  |
| 2-NNAP | 0 |  |  |  |  |  | 0 |  |  |  |  |  |
| 9-NANT | 11 | 0.15 | 4.61 | 1.37 | 0.67 | 1.45 | 0 |  |  |  |  |  |
| 3-NPHE | 1 |  |  | 1.40 | 1.40 |  | 0 |  |  |  |  |  |
| 2-NFLT | 26 | 0.02 | 5.90 | 1.17 | 0.70 | 1.32 | 0 |  |  |  |  |  |
| 3-NFLT | 15 | 0.18 | 5.49 | 1.41 | 1.16 | 1.36 | 2 | 0.004 | 5.74 | 2.87 | 2.87 | 4.05 |
| 1-NPYR | 20 | 0.29 | 5.61 | 1.59 | 0.95 | 1.50 | 8 | 0.001 | 2.99 | 1.96 | 2.23 | 0.96 |
| 2-NPYR | 20 | 0.10 | 10.69 | 1.64 | 0.58 | 2.50 | 0 |  |  |  |  |  |
| 7-NBAA | 14 | 0.04 | 14.76 | 2.07 | 0.51 | 3.84 | 0 |  |  |  |  |  |
| **Σ9NPAHs** | 26 | 0.01 | 1.63 | 0.55 | 0.44 | 0.42 | 9 | 0.01 | 78.80 | 27.45 | 18.84 | 25.51 |
| 1,4-O_2_NAP | 16 | 0.57 | 1.75 | 1.08 | 1.11 | 0.39 | 0 |  |  |  |  |  |
| 1(CHO)NAP | 15 | 0.70 | 1.28 | 0.94 | 0.97 | 0.18 | 0 |  |  |  |  |  |
| 9-OFLN | 13 | 0.46 | 1.82 | 1.04 | 0.94 | 0.35 | 0 |  |  |  |  |  |
| 9,10-O_2_ANT | 8 | 0.34 | 1.52 | 0.87 | 0.78 | 0.44 | 0 |  |  |  |  |  |
| 1,4-O_2_ANT | 0 |  |  |  |  |  | 0 |  |  |  |  |  |
| 9,10-O_2_PHE | 0 |  |  |  |  |  | 0 |  |  |  |  |  |
| BaOFLN | 11 | 0.16 | 1.43 | 0.76 | 0.75 | 0.40 | 0 |  |  |  |  |  |
| BbOFLN | 5 | 0.25 | 0.97 | 0.62 | 0.57 | 0.28 | 0 |  |  |  |  |  |
| BAN | 25 | 0.13 | 8.33 | 1.34 | 0.85 | 1.69 | 0 |  |  |  |  |  |
| 7,12-O_2_BAA | 18 | 0.18 | 2.53 | 0.84 | 0.69 | 0.54 | 0 |  |  |  |  |  |
| 5,12-O_2_NAC | 23 | 0.11 | 16.04 | 1.54 | 0.56 | 3.26 | 0 |  |  |  |  |  |
| **Σ11OPAHs** | 26 | 0.06 | 0.91 | 0.46 | 0.45 | 0.22 | 2 | 0.93 | 2.45 | 1.69 | 1.69 | 1.07 |

Table S13: Traffic-to-urban background ratios of individual NOPAHs at Brno. Only the samples collected simultanouesly were considered

|  | Winter | | | | | | Summer | | | | | |
| --- | --- | --- | --- | --- | --- | --- | --- | --- | --- | --- | --- | --- |
|  | N | Min | Max | Average | Median | SD | N | Min | Max | Average | Median | SD |
| 1-NNAP | 8 | 0.90 | 2.38 | 1.46 | 1.41 | 0.42 | 0 |  |  |  |  |  |
| 2-NNAP | 0 |  |  |  |  |  | 7 | 0.02 | 0.06 | 0.04 | 0.05 | 0.02 |
| 9-NANT | 22 | 1.41 | 31.63 | 10.65 | 9.44 | 7.86 | 0 |  |  |  |  |  |
| 3-NPHE | 0 |  |  |  |  |  | 0 |  |  |  |  |  |
| 2-NFLT | 30 | 0.57 | 4.01 | 1.75 | 1.78 | 0.79 | 0 |  |  |  |  |  |
| 3-NFLT | 11 | 1.34 | 3.68 | 2.15 | 2.00 | 0.72 | 0 |  |  |  |  |  |
| 1-NPYR | 30 | 1.43 | 7.85 | 3.52 | 3.12 | 1.60 | 0 |  |  |  |  |  |
| 2-NPYR | 30 | 0.50 | 4.44 | 1.60 | 1.26 | 0.94 | 0 |  |  |  |  |  |
| 7-NBAA | 11 | 1.25 | 8.67 | 3.92 | 2.52 | 2.57 | 0 |  |  |  |  |  |
| **Σ9NPAHs** | 30 | 1.19 | 7.57 | 4.02 | 3.53 | 1.67 | 8 | 0.31 | 1.76 | 1.10 | 1.06 | 0.52 |
| 1,4-O_2_NAP | 11 | 0.24 | 1.03 | 0.69 | 0.71 | 0.23 | 0 |  |  |  |  |  |
| 1(CHO)NAP | 7 | 0.42 | 2.89 | 1.41 | 1.33 | 0.81 | 0 |  |  |  |  |  |
| 9-OFLN | 2 | 0.74 | 0.75 | 0.75 | 0.75 | 0.01 | 0 |  |  |  |  |  |
| 9,10-O_2_ANT | 14 | 0.51 | 2.86 | 2.01 | 2.08 | 0.78 | 0 |  |  |  |  |  |
| 1,4-O_2_ANT | 0 |  |  |  |  |  | 0 |  |  |  |  |  |
| 9,10-O_2_PHE | 0 |  |  |  |  |  | 0 |  |  |  |  |  |
| BaOFLN | 30 | 0.79 | 4.20 | 2.20 | 1.98 | 0.95 | 2 | 1.16 | 1.23 | 1.19 | 1.19 | 0.05 |
| BbOFLN | 27 | 0.16 | 3.94 | 1.75 | 1.70 | 0.79 | 1 |  |  | 0.68 | 0.68 |  |
| BAN | 30 | 0.42 | 2.41 | 1.13 | 1.04 | 0.48 | 1 |  |  | 0.55 | 0.55 |  |
| 7,12-O_2_BAA | 29 | 0.84 | 3.43 | 1.51 | 1.41 | 0.56 | 1 |  |  | 0.53 | 0.53 |  |
| 5,12-O_2_NAC | 30 | 0.64 | 3.70 | 1.48 | 1.24 | 0.76 | 0 |  |  |  |  |  |
| **Σ11OPAHs** | 30 | 0.60 | 6.02 | 2.66 | 2.42 | 1.42 | 2 | 1.58 | 10.25 | 5.91 | 5.91 | 6.13 |

Table S14: Brno urban background-to-rural ratios of individual NOPAHs. Only the samples collected simultanouesly were considered

|  | Winter | | | | | | Summer | | | | | |
| --- | --- | --- | --- | --- | --- | --- | --- | --- | --- | --- | --- | --- |
|  | N | Min | Max | Average | Median | SD | N | Min | Max | Average | Median | SD |
| 1-NNAP | 1 |  |  | 6.65 | 6.65 |  | 0 |  |  |  |  |  |
| 2-NNAP | 0 |  |  |  |  |  | 0 |  |  |  |  |  |
| 9-NANT | 8 | 0.13 | 11.48 | 3.55 | 1.57 | 4.01 | 0 |  |  |  |  |  |
| 3-NPHE | 0 |  |  |  |  |  | 0 |  |  |  |  |  |
| 2-NFLT | 8 | 1.47 | 13.93 | 5.62 | 5.24 | 4.05 | 0 |  |  |  |  |  |
| 3-NFLT | 3 | 4.97 | 17.18 | 12.44 | 15.15 | 6.54 | 0 |  |  |  |  |  |
| 1-NPYR | 7 | 3.95 | 17.31 | 12.47 | 15.56 | 5.21 | 0 |  |  |  |  |  |
| 2-NPYR | 8 | 6.95 | 29.45 | 18.07 | 19.03 | 8.60 | 0 |  |  |  |  |  |
| 7-NBAA | 3 | 3.51 | 13.20 | 7.67 | 6.30 | 4.99 | 0 |  |  |  |  |  |
| **Σ9NPAHs** | 8 | 0.74 | 14.51 | 5.29 | 3.51 | 4.92 | 0 |  |  |  |  |  |
| 1,4-O_2_NAP | 3 | 23.58 | 48.10 | 36.38 | 37.46 | 12.30 | 0 |  |  |  |  |  |
| 1(CHO)NAP | 3 | 7.90 | 23.59 | 13.97 | 10.42 | 8.43 | 0 |  |  |  |  |  |
| 9-OFLN | 1 |  |  | 11.28 | 11.28 |  | 0 |  |  |  |  |  |
| 9,10-O_2_ANT | 5 | 0.58 | 15.20 | 4.75 | 2.94 | 6.04 | 0 |  |  |  |  |  |
| 1,4-O_2_ANT | 0 |  |  |  |  |  | 0 |  |  |  |  |  |
| 9,10-O_2_PHE | 0 |  |  |  |  |  | 0 |  |  |  |  |  |
| BaOFLN | 8 | 1.48 | 24.62 | 7.57 | 5.13 | 7.57 | 1 |  |  | 14.91 | 14.91 |  |
| BbOFLN | 8 | 1.38 | 35.68 | 9.30 | 5.18 | 11.42 | 0 |  |  |  |  |  |
| BAN | 8 | 1.59 | 31.91 | 9.90 | 5.94 | 10.28 | 0 |  |  |  |  |  |
| 7,12-O_2_BAA | 8 | 1.34 | 17.96 | 5.98 | 4.36 | 5.63 | 0 |  |  |  |  |  |
| 5,12-O_2_NAC | 8 | 1.68 | 32.46 | 9.50 | 6.20 | 10.32 | 0 |  |  |  |  |  |
| **Σ11OPAHs** | 8 | 0.58 | 18.87 | 4.88 | 2.95 | 6.08 | 1 |  |  | 0.52 | 0.52 |  |

Table S15: Results of the Spearman correlation analysis (r) at the rural site in winter between the individual concentrations of NOPAHs and different meteorological parameters (WD = wind direction, WS = wind speed, RH = relative humidity, T = temperature) or air pollutants. Numbers in bold indicate cases significant at the 95% confidence level, while those underlined are significant at the 99% confidence level

|  | WD | WS | RH | T | SO_2_ | PM_2.5_ | PM_10_ | O_3_ | NO_x_ | NO_2_ | NO | CO | Zn | OC | EC |
| --- | --- | --- | --- | --- | --- | --- | --- | --- | --- | --- | --- | --- | --- | --- | --- |
| 1-NNAP | -0.20 | -0.80 | -0.40 | -0.40 | 0.80 | 0.20 | 0.20 | 0.20 | 0.40 | 0.40 | 0.80 | 1.00 |  | 0.80 | 0.80 |
| 2-NNAP |  |  |  |  |  |  |  |  |  |  |  |  |  |  |  |
| 9-NANT | -0.52 | **-0.95** | **-0.79** | -0.43 | **0.74** | 0.67 | 0.67 | 0.57 | 0.29 | 0.26 | 0.49 | **0.79** | 0.30 | **0.83** | **0.83** |
| 3-NPHE | -0.80 | -1.00 | -0.80 | -0.60 | 0.20 | 0.20 | 0.20 | 0.40 | 0.40 | 0.21 | 0.40 | 0.40 | 0.50 | 1.00 | 1.00 |
| 2-NFLT | -0.62 | -0.71 | -0.67 | -0.52 | **0.90** | **0.83** | **0.83** | 0.69 | 0.40 | 0.40 | 0.56 | **0.98** | 0.70 | **0.95** | **0.95** |
| 3-NFLT | -0.21 | -0.71 | -0.75 | -0.07 | 0.64 | 0.46 | 0.46 | 0.36 | 0.61 | 0.59 | **0.79** | **0.82** | -0.60 | 0.71 | 0.71 |
| 1-NPYR | -0.71 | -0.61 | -0.64 | -0.64 | **0.82** | 0.71 | 0.71 | 0.75 | 0.21 | 0.18 | 0.43 | **0.89** | 0.80 | **0.96** | **0.96** |
| 2-NPYR | -0.64 | **-0.83** | -0.71 | -0.62 | **0.81** | **0.74** | **0.74** | 0.64 | 0.33 | 0.30 | 0.51 | **0.88** | 0.70 | **0.95** | **0.95** |
| 7-NBAA | -0.25 | **-0.96** | **-0.79** | -0.18 | 0.46 | 0.29 | 0.29 | 0.32 | 0.21 | 0.18 | 0.50 | 0.54 | -0.40 | 0.61 | 0.61 |
| **Σ9NPAHs** | -0.45 | **-0.93** | **-0.81** | -0.29 | **0.76** | 0.69 | 0.69 | 0.55 | 0.43 | 0.42 | 0.61 | **0.83** | 0.10 | **0.81** | **0.81** |
| 1,4-O_2_NAP | -0.80 | 0.20 | 0.40 | -0.80 | 0.20 | 0.20 | 0.20 | 0.40 | 0.00 | -0.21 | 0.00 | 0.40 | 0.80 | 1.00 | 1.00 |
| 1(CHO)NAP | 0.50 | -0.50 | -0.50 | 0.50 | -0.50 | -0.50 | -0.50 | -0.50 | 1.00 | 1.00 | 1.00 | 0.50 | -0.50 | 0.50 | 0.50 |
| 9-OFLN |  |  |  |  |  |  |  |  |  |  |  |  |  |  |  |
| 9,10-O_2_ANT | -0.62 | -0.71 | -0.52 | **-0.74** | **0.81** | 0.69 | 0.69 | 0.60 | 0.14 | 0.11 | 0.36 | **0.86** | 0.90 | **0.95** | **0.95** |
| 1,4-O_2_ANT | **-0.89** | -0.43 | -0.60 | -0.77 | 0.77 | 0.71 | 0.71 | 0.77 | 0.09 | 0.03 | 0.09 | 0.83 | 0.80 | **1.00** | **1.00** |
| 9,10-O_2_PHE | -0.61 | **-0.86** | **-0.79** | -0.54 | 0.64 | 0.54 | 0.54 | 0.61 | 0.21 | 0.16 | 0.46 | 0.71 | 0.20 | **0.86** | **0.86** |
| BaOFLN | **-0.76** | **-0.76** | -0.71 | -0.71 | **0.90** | **0.83** | **0.83** | **0.76** | 0.19 | 0.17 | 0.32 | **0.90** | 0.90 | **1.00** | **1.00** |
| BbOFLN | -0.69 | **-0.74** | -0.64 | -0.67 | **0.88** | **0.81** | **0.81** | 0.71 | 0.26 | 0.24 | 0.44 | **0.93** | 0.90 | **0.98** | **0.98** |
| BAN | -0.64 | **-0.83** | -0.71 | -0.62 | **0.81** | **0.74** | **0.74** | 0.64 | 0.33 | 0.30 | 0.51 | **0.88** | 0.70 | **0.95** | **0.95** |
| 7,12-O_2_BAA | -0.69 | **-0.74** | -0.64 | -0.67 | **0.88** | **0.81** | **0.81** | 0.71 | 0.26 | 0.24 | 0.44 | **0.93** | 0.90 | **0.98** | **0.98** |
| 5,12-O_2_NAC | -0.69 | **-0.74** | -0.64 | -0.67 | **0.88** | **0.81** | **0.81** | 0.71 | 0.26 | 0.24 | 0.44 | **0.93** | 0.90 | **0.98** | **0.98** |
| **Σ11OPAHs** | -0.64 | **-0.83** | -0.71 | -0.62 | **0.81** | **0.74** | **0.74** | 0.64 | 0.33 | 0.30 | 0.51 | **0.88** | 0.70 | **0.95** | **0.95** |

Table S16: Results of the Spearman correlation analysis (r) at the Brno urban background site in winter between the individual concentrations of NOPAHs and different meteorological parameters (WD = wind direction, WS = wind speed, RH = relative humidity, T = temperature) or air pollutants. Numbers in bold indicate cases significant at the 95% confidence level, while those underlined are significant at the 99% confidence level

|  | WD | WS | RH | T | SO_2_ | PM_2.5_ | PM_10_ | O_3_ | NO_x_ | NO_2_ | NO | CO | PM_1_ | Cu | Zn | OC | EC |
| --- | --- | --- | --- | --- | --- | --- | --- | --- | --- | --- | --- | --- | --- | --- | --- | --- | --- |
| 1-NNAP | 0.22 | **-0.82** | 0.20 | **-0.77** | -0.12 | 0.54 | **0.72** | -0.49 | **0.76** | 0.52 | **0.76** | **0.72** | **0.72** | 0.25 | **0.83** | **0.95** | **0.96** |
| 2-NNAP |  |  |  |  |  |  |  |  |  |  |  |  |  |  |  |  |  |
| 9-NANT | -0.08 | 0.09 | -0.39 | **-0.63** | **0.43** | **0.61** | **0.48** | 0.23 | 0.11 | 0.17 | 0.13 | 0.18 | **0.56** | **0.61** | **0.82** | **0.73** | 0.33 |
| 3-NPHE |  |  |  |  |  |  |  |  |  |  |  |  |  |  |  |  |  |
| 2-NFLT | -0.19 | **-0.59** | **0.47** | **-0.89** | **0.60** | **0.87** | **0.85** | **-0.59** | **0.47** | **0.63** | **0.41** | **0.77** | **0.90** | **0.40** | **0.86** | **0.90** | **0.48** |
| 3-NFLT | -0.25 | 0.28 | **-0.78** | **-0.75** | 0.56 | **0.88** | **0.72** | 0.27 | 0.27 | 0.39 | 0.27 | 0.04 | **0.81** | **0.66** | **0.89** | **0.92** | **0.67** |
| 1-NPYR | -0.16 | **-0.59** | **0.49** | **-0.89** | **0.62** | **0.87** | **0.83** | **-0.60** | **0.48** | **0.62** | **0.43** | **0.78** | **0.90** | **0.42** | **0.86** | **0.91** | **0.48** |
| 2-NPYR | -0.11 | **-0.60** | **0.45** | **-0.91** | **0.52** | **0.85** | **0.82** | **-0.58** | **0.52** | **0.61** | **0.48** | **0.77** | **0.90** | **0.44** | **0.92** | **0.94** | **0.58** |
| 7-NBAA | -0.30 | 0.32 | **-0.81** | **-0.72** | 0.58 | **0.84** | **0.71** | 0.24 | 0.22 | 0.37 | 0.22 | -0.03 | **0.79** | **0.62** | **0.88** | **0.92** | **0.64** |
| **Σ9NPAHs** | -0.13 | **-0.58** | **0.41** | **-0.87** | **0.57** | **0.86** | **0.82** | **-0.55** | **0.45** | **0.60** | **0.39** | **0.75** | **0.89** | **0.46** | **0.89** | **0.91** | **0.49** |
| 1,4-O_2_NAP | -0.07 | 0.41 | -0.56 | **-0.76** | 0.64 | **0.80** | 0.55 | 0.24 | 0.21 | 0.27 | 0.21 | 0.01 | **0.67** | 0.27 | **0.79** | **0.83** | **0.67** |
| 1(CHO)NAP | -0.09 | 0.60 | 0.37 | -0.43 | 0.83 | 0.54 | 0.43 | 0.14 | -0.60 | -0.43 | -0.60 | -0.14 | 0.54 | -0.29 | 0.39 | 0.43 | -0.04 |
| 9-OFLN |  |  |  |  |  |  |  |  |  |  |  |  |  |  |  |  |  |
| 9,10-O_2_ANT | -0.46 | 0.42 | -0.43 | **-0.63** | **0.79** | **0.88** | 0.45 | 0.30 | -0.11 | 0.20 | -0.11 | -0.10 | 0.55 | **0.57** | **0.67** | **0.62** | 0.07 |
| 1,4-O_2_ANT |  |  |  |  |  |  |  |  |  |  |  |  |  |  |  |  |  |
| 9,10-O_2_PHE |  |  |  |  |  |  |  |  |  |  |  |  |  |  |  |  |  |
| BaOFLN | -0.14 | **-0.59** | **0.48** | **-0.89** | **0.59** | **0.86** | **0.82** | **-0.59** | **0.42** | **0.60** | 0.36 | **0.77** | **0.90** | **0.44** | **0.85** | **0.89** | **0.43** |
| BbOFLN | -0.10 | **-0.46** | 0.34 | **-0.89** | **0.59** | **0.83** | **0.81** | **-0.43** | 0.25 | **0.49** | 0.20 | **0.70** | **0.90** | **0.40** | **0.82** | **0.87** | 0.25 |
| BAN | -0.11 | **-0.61** | **0.48** | **-0.89** | **0.57** | **0.85** | **0.82** | **-0.60** | **0.44** | **0.61** | **0.37** | **0.77** | **0.89** | **0.41** | **0.84** | **0.89** | **0.44** |
| 7,12-O_2_BAA | -0.21 | **-0.55** | **0.44** | **-0.87** | **0.58** | **0.86** | **0.84** | **-0.55** | 0.36 | **0.55** | 0.29 | **0.75** | **0.90** | **0.43** | **0.85** | **0.89** | **0.37** |
| 5,12-O_2_NAC | -0.13 | **-0.56** | **0.49** | **-0.87** | **0.61** | **0.84** | **0.80** | **-0.57** | **0.38** | **0.56** | 0.32 | **0.75** | **0.88** | **0.41** | **0.84** | **0.87** | **0.39** |
| **Σ11OPAHs** | -0.12 | **-0.58** | **0.47** | **-0.89** | **0.58** | **0.84** | **0.81** | **-0.58** | **0.41** | **0.59** | 0.35 | **0.75** | **0.89** | **0.43** | **0.85** | **0.88** | **0.43** |

Table S17: Results of the Spearman correlation analysis (r) at the Brno traffic site in winter between the individual concentrations of NOPAHs and different meteorological parameters (WD = wind direction, WS = wind speed, RH = relative humidity, T = temperature) or air pollutants. Numbers in bold indicate cases significant at the 95% confidence level, while those underlined are significant at the 99% confidence level

|  | PM_2.5_ | Cu | Zn | OC | EC |
| --- | --- | --- | --- | --- | --- |
| 1-NNAP | **0.73** | **0.57** | **0.73** | **0.84** | **0.60** |
| 2-NNAP | **0.76** | **0.61** | **0.76** | **0.85** | **0.60** |
| 9-NANT | **0.79** | **0.54** | **0.76** | **0.89** | **0.58** |
| 3-NPHE | **0.75** | **0.60** | **0.72** | **0.86** | **0.63** |
| 2-NFLT | **0.83** | **0.60** | **0.80** | **0.90** | **0.50** |
| 3-NFLT | **0.81** | **0.67** | **0.73** | **0.85** | 0.34 |
| 1-NPYR | **0.83** | **0.60** | **0.82** | **0.90** | **0.58** |
| 2-NPYR | **0.84** | **0.61** | **0.82** | **0.92** | **0.57** |
| 7-NBAA | **0.63** | **0.56** | **0.57** | **0.70** | 0.37 |
| **Σ9NPAHs** | **0.80** | **0.58** | **0.78** | **0.89** | **0.55** |
| 1,4-O_2_NAP | 0.49 | 0.45 | **0.66** | **0.53** | -0.09 |
| 1(CHO)NAP | **0.58** | **0.52** | **0.62** | **0.61** | 0.09 |
| 9-OFLN | **0.78** | 0.02 | **0.86** | **0.79** | 0.00 |
| 9,10-O_2_ANT | **0.81** | **0.52** | **0.74** | **0.89** | **0.50** |
| 1,4-O_2_ANT | **0.61** | 0.29 | **0.63** | **0.80** | **0.81** |
| 9,10-O_2_PHE | **0.57** | 0.23 | **0.68** | **0.86** | **0.84** |
| BaOFLN | **0.84** | **0.56** | **0.76** | **0.90** | **0.48** |
| BbOFLN | **0.82** | **0.58** | **0.77** | **0.90** | **0.48** |
| BAN | **0.84** | **0.58** | **0.80** | **0.91** | **0.57** |
| 7,12-O_2_BAA | **0.85** | **0.60** | **0.80** | **0.93** | **0.53** |
| 5,12-O_2_NAC | **0.84** | **0.59** | **0.82** | **0.93** | **0.52** |
| **Σ11OPAHs** | **0.84** | **0.56** | **0.80** | **0.92** | **0.51** |

Table S18: Results of the Spearman correlation analysis (r) at the Ljubljana urban background site in winter between the individual concentrations of NOPAHs and different meteorological parameters (WD = wind direction, WS = wind speed, RH = relative humidity, T = temperature) or air pollutants. Numbers in bold indicate cases significant at the 95% confidence level, while those underlined are significant at the 99% confidence level

|  | WS | RH | T | SO_2_ | PM_2.5_ | O_3_ | NO_2_ | CO | Cu | Zn | OC | EC |
| --- | --- | --- | --- | --- | --- | --- | --- | --- | --- | --- | --- | --- |
| 1-NNAP | -0.60 | -0.40 | 0.60 | -0.10 | 0.10 | -0.30 | 0.60 | 0.50 | -0.31 | 0.17 | 0.19 | 0.57 |
| 2-NNAP |  |  |  |  |  |  |  |  |  |  |  |  |
| 9-NANT | **-0.75** | -0.45 | 0.02 | **-0.64** | **0.47** | **-0.70** | **0.81** | **0.88** | **0.55** | **0.74** | **0.92** | **0.92** |
| 3-NPHE | -0.50 | -1.00 | 0.50 | -1.00 | 0.50 | 0.50 | -0.50 | 0.50 | 0.20 | 0.70 | 0.00 | 0.70 |
| 2-NFLT | **-0.79** | -0.19 | -0.35 | 0.02 | 0.02 | **-0.81** | **0.81** | **0.69** | **0.53** | **0.57** | **0.69** | **0.80** |
| 3-NFLT | **-0.75** | -0.33 | -0.25 | **-0.60** | **0.56** | **-0.84** | **0.80** | **0.84** | 0.24 | 0.42 | **0.81** | **0.81** |
| 1-NPYR | **-0.70** | -0.36 | -0.13 | **-0.67** | **0.46** | **-0.79** | **0.79** | **0.85** | 0.42 | **0.72** | **0.82** | **0.89** |
| 2-NPYR | **-0.81** | -0.45 | -0.11 | **-0.72** | **0.48** | **-0.76** | **0.86** | **0.96** | 0.24 | **0.55** | **0.92** | **0.89** |
| 7-NBAA | **-0.82** | 0.10 | -0.41 | -0.26 | 0.43 | **-0.83** | **0.85** | **0.72** | 0.32 | 0.52 | **0.81** | **0.85** |
| **Σ9NPAHs** | **-0.81** | -0.15 | **-0.43** | 0.05 | -0.04 | **-0.82** | **0.79** | **0.62** | **0.48** | **0.55** | **0.66** | **0.77** |
| 1,4-O_2_NAP | **-0.60** | -0.14 | -0.37 | -0.39 | 0.41 | **-0.74** | **0.66** | **0.62** | -0.14 | 0.17 | **0.56** | **0.59** |
| 1(CHO)NAP | -0.17 | 0.37 | **-0.54** | 0.14 | 0.01 | -0.39 | 0.15 | -0.12 | -0.34 | -0.30 | -0.01 | 0.18 |
| 9-OFLN | -0.36 | -0.07 | -0.40 | -0.34 | 0.23 | **-0.55** | 0.47 | 0.43 | 0.15 | 0.22 | **0.51** | **0.56** |
| 9,10-O_2_ANT | -0.16 | -0.41 | -0.02 | -0.10 | 0.33 | -0.04 | 0.26 | 0.53 | 0.06 | 0.37 | **0.60** | **0.67** |
| 1,4-O_2_ANT |  |  |  |  |  |  |  |  |  |  |  |  |
| 9,10-O_2_PHE |  |  |  |  |  |  |  |  |  |  |  |  |
| BaOFLN | -0.10 | -0.40 | -0.29 | -0.06 | 0.16 | -0.22 | 0.42 | **0.59** | 0.33 | 0.49 | **0.62** | **0.71** |
| BbOFLN | -0.29 | -0.07 | -0.71 | 0.32 | 0.12 | -0.25 | 0.43 | 0.57 | 0.55 | **0.76** | 0.69 | **0.76** |
| BAN | **-0.80** | -0.18 | **-0.50** | 0.03 | -0.05 | **-0.80** | **0.78** | **0.61** | **0.56** | **0.54** | **0.64** | **0.79** |
| 7,12-O_2_BAA | **-0.79** | **-0.44** | -0.17 | -0.31 | 0.14 | **-0.79** | **0.86** | **0.76** | **0.52** | **0.60** | **0.80** | **0.91** |
| 5,12-O_2_NAC | **-0.80** | -0.32 | -0.35 | -0.31 | 0.07 | **-0.79** | **0.82** | **0.70** | 0.40 | **0.51** | **0.70** | **0.83** |
| **Σ11OPAHs** | **-0.83** | -0.20 | **-0.48** | 0.02 | 0.00 | **-0.79** | **0.79** | **0.66** | **0.56** | **0.59** | **0.69** | **0.82** |

Table S19: Results of the Spearman correlation analysis (r) at the Ljubljana traffic site in winter between the individual concentrations of NOPAHs and different meteorological parameters (WD = wind direction, WS = wind speed, RH = relative humidity, T = temperature) or air pollutants. Numbers in bold indicate cases significant at the 95% confidence level, while those underlined are significant at the 99% confidence level

|  | WS | RH | T | SO_2_ | PM_2.5_ | NO_2_ | Cu | Zn | OC | EC |
| --- | --- | --- | --- | --- | --- | --- | --- | --- | --- | --- |
| 1-NNAP | -0.52 | -0.03 | **-0.69** | -0.16 | 0.07 | 0.35 | -0.54 | 0.00 | 0.48 | 0.52 |
| 2-NNAP |  |  |  |  |  |  |  |  |  |  |
| 9-NANT | -0.41 | -0.50 | -0.34 | -0.09 | **0.86** | 0.50 | 0.29 | **0.75** | **0.91** | **0.88** |
| 3-NPHE |  |  |  |  |  |  |  |  |  |  |
| 2-NFLT | -0.36 | 0.18 | **-0.51** | -0.01 | 0.09 | **0.40** | 0.38 | **0.47** | **0.75** | **0.69** |
| 3-NFLT | -0.43 | -0.01 | -0.49 | 0.07 | **0.48** | **0.53** | 0.06 | **0.68** | **0.87** | **0.71** |
| 1-NPYR | -0.39 | -0.09 | -0.35 | 0.12 | 0.39 | 0.41 | 0.36 | **0.44** | **0.86** | **0.82** |
| 2-NPYR | **-0.44** | -0.14 | **-0.50** | 0.00 | 0.38 | **0.53** | 0.42 | **0.53** | **0.84** | **0.86** |
| 7-NBAA | **-0.57** | 0.38 | -0.38 | 0.34 | 0.36 | 0.43 | 0.24 | 0.54 | **0.83** | **0.84** |
| **Σ9NPAHs** | -0.37 | 0.11 | **-0.44** | 0.02 | 0.12 | **0.39** | **0.49** | **0.51** | **0.75** | **0.73** |
| 1,4-O_2_NAP | -0.35 | 0.02 | **-0.62** | -0.10 | **0.48** | **0.58** | -0.44 | 0.44 | **0.75** | **0.51** |
| 1(CHO)NAP | -0.21 | 0.47 | **-0.48** | -0.02 | -0.06 | 0.10 | -0.41 | 0.17 | 0.24 | 0.23 |
| 9-OFLN | -0.33 | 0.42 | **-0.63** | -0.11 | 0.17 | 0.25 | -0.32 | 0.28 | 0.38 | 0.33 |
| 9,10-O_2_ANT | **-0.64** | 0.30 | -0.27 | 0.02 | 0.13 | 0.21 | 0.20 | 0.12 | **0.79** | **0.71** |
| 1,4-O_2_ANT |  |  |  |  |  |  |  |  |  |  |
| 9,10-O_2_PHE |  |  |  |  |  |  |  |  |  |  |
| BaOFLN | -0.29 | -0.02 | 0.01 | 0.03 | 0.39 | 0.25 | 0.26 | **0.48** | **0.80** | **0.80** |
| BbOFLN | -0.53 | 0.33 | 0.07 | 0.40 | **0.70** | 0.62 | **0.70** | 0.55 | **0.88** | **0.90** |
| BAN | -0.32 | 0.01 | **-0.48** | -0.06 | 0.19 | **0.41** | **0.48** | **0.61** | **0.73** | **0.79** |
| 7,12-O_2_BAA | **-0.43** | -0.11 | -0.29 | 0.08 | 0.37 | 0.34 | **0.45** | **0.67** | **0.78** | **0.89** |
| 5,12-O_2_NAC | **-0.58** | -0.04 | -0.36 | 0.19 | 0.32 | **0.51** | 0.36 | **0.56** | **0.76** | **0.83** |
| **Σ11OPAHs** | **-0.37** | -0.10 | **-0.45** | -0.03 | 0.26 | **0.48** | **0.50** | **0.65** | **0.76** | **0.80** |

Table S20: Results of the Spearman correlation analysis (r) at the rural site in summer between the individual concentrations of NOPAHs and different meteorological parameters (WD = wind direction, WS = wind speed, RH = relative humidity, T = temperature) or air pollutants. Numbers in bold indicate cases significant at the 95% confidence level, while those underlined are significant at the 99% confidence level

|  | WD | WS | RH | T | SO_2_ | PM_2.5_ | PM_10_ | O_3_ | NO_x_ | NO_2_ | NO | CO | Cu | Zn | OC | EC |
| --- | --- | --- | --- | --- | --- | --- | --- | --- | --- | --- | --- | --- | --- | --- | --- | --- |
| 1-NNAP |  |  |  |  |  |  |  |  |  |  |  |  |  |  |  |  |
| 2-NNAP |  |  |  |  |  |  |  |  |  |  |  |  |  |  |  |  |
| 9-NANT |  |  |  |  |  |  |  |  |  |  |  |  |  |  |  |  |
| 3-NPHE |  |  |  |  |  |  |  |  |  |  |  |  |  |  |  |  |
| 2-NFLT |  |  |  |  |  |  |  |  |  |  |  |  |  |  |  |  |
| 3-NFLT |  |  |  |  |  |  |  |  |  |  |  |  |  |  |  |  |
| 1-NPYR |  |  |  |  |  |  |  |  |  |  |  |  |  |  |  |  |
| 2-NPYR |  |  |  |  |  |  |  |  |  |  |  |  |  |  |  |  |
| 7-NBAA |  |  |  |  |  |  |  |  |  |  |  |  |  |  |  |  |
| **Σ9NPAHs** |  |  |  |  |  |  |  |  |  |  |  |  |  |  |  |  |
| 1,4-O_2_NAP | 0.14 | 0.39 | 0.18 | -0.04 | 0.64 | 0.11 | -0.14 | 0.36 | 0.43 | 0.43 | -0.40 | 0.43 | -0.50 | 0.32 | -0.25 | -0.32 |
| 1(CHO)NAP | 0.10 | -0.30 | -0.80 | 0.60 | -0.10 | 0.60 | 0.60 | **1.00** | **1.00** | **1.00** | 0.30 | **1.00** | -0.50 | -0.20 | 0.60 | 0.70 |
| 9-OFLN |  |  |  |  |  |  |  |  |  |  |  |  |  |  |  |  |
| 9,10-O_2_ANT |  |  |  |  |  |  |  |  |  |  |  |  |  |  |  |  |
| 1,4-O_2_ANT |  |  |  |  |  |  |  |  |  |  |  |  |  |  |  |  |
| 9,10-O_2_PHE |  |  |  |  |  |  |  |  |  |  |  |  |  |  |  |  |
| BaOFLN | -0.80 | -0.90 | -0.80 | -0.20 | -0.70 | 0.60 | 0.50 | 0.70 | 0.50 | 0.50 | 0.21 | 0.00 |  | -0.30 | -0.10 | 0.82 |
| BbOFLN | **-0.79** | -0.43 | -0.21 | 0.07 | -0.57 | 0.18 | -0.21 | 0.14 | -0.50 | -0.32 | -0.25 | -0.04 |  | 0.04 | 0.04 | 0.58 |
| BAN | **-0.93** | -0.21 | -0.50 | 0.26 | -0.45 | 0.13 | -0.07 | 0.12 | -0.55 | -0.40 | -0.66 | -0.05 | 1.00 | 0.14 | 0.14 | 0.68 |
| 7,12-O_2_BAA |  |  |  |  |  |  |  |  |  |  |  |  |  |  |  |  |
| 5,12-O_2_NAC | -0.71 | -0.50 | -0.26 | 0.12 | **-0.76** | 0.04 | -0.17 | 0.02 | -0.52 | -0.38 | -0.20 | -0.12 | 1.00 | -0.10 | 0.19 | 0.66 |
| **Σ11OPAHs** | -0.60 | -0.19 | -0.36 | 0.05 | -0.43 | 0.05 | -0.29 | 0.38 | 0.00 | 0.12 | -0.60 | 0.21 | 1.00 | -0.10 | -0.07 | 0.50 |

Table S21: Results of the Spearman correlation analysis (r) at the Brno urban background site in summer between the individual concentrations of NOPAHs and different meteorological parameters (WD = wind direction, WS = wind speed, RH = relative humidity, T = temperature) or air pollutants. indicate cases significant at the 95% confidence level, while those underlined are significant at the 99% confidence level

|  | SO_2_ | PM_2.5_ | PM_10_ | O_3_ | NO_x_ | NO_2_ | NO | CO | Cu | Zn | OC | EC |
| --- | --- | --- | --- | --- | --- | --- | --- | --- | --- | --- | --- | --- |
| 1-NNAP |  |  |  |  |  |  |  |  |  |  |  |  |
| 2-NNAP | -0.71 | -0.10 | 0.07 | 0.02 | -0.17 | -0.36 | 0.05 | 0.05 | 0.40 | 0.17 | -0.26 | 0.38 |
| 9-NANT |  |  |  |  |  |  |  |  |  |  |  |  |
| 3-NPHE |  |  |  |  |  |  |  |  |  |  |  |  |
| 2-NFLT |  |  |  |  |  |  |  |  |  |  |  |  |
| 3-NFLT |  |  |  |  |  |  |  |  |  |  |  |  |
| 1-NPYR |  |  |  |  |  |  |  |  |  |  |  |  |
| 2-NPYR |  |  |  |  |  |  |  |  |  |  |  |  |
| 7-NBAA |  |  |  |  |  |  |  |  |  |  |  |  |
| **Σ9NPAHs** | 0.36 | 0.26 | 0.45 | 0.34 | -0.10 | -0.10 | -0.18 | 0.12 | -0.36 | -0.04 | **0.50** | 0.12 |
| 1,4-O_2_NAP |  |  |  |  |  |  |  |  |  |  |  |  |
| 1(CHO)NAP |  |  |  |  |  |  |  |  |  |  |  |  |
| 9-OFLN |  |  |  |  |  |  |  |  |  |  |  |  |
| 9,10-O_2_ANT |  |  |  |  |  |  |  |  |  |  |  |  |
| 1,4-O_2_ANT |  |  |  |  |  |  |  |  |  |  |  |  |
| 9,10-O_2_PHE |  |  |  |  |  |  |  |  |  |  |  |  |
| BaOFLN |  |  |  |  |  |  |  |  |  |  |  |  |
| BbOFLN |  |  |  |  |  |  |  |  |  |  |  |  |
| BAN |  |  |  |  |  |  |  |  |  |  |  |  |
| 7,12-O_2_BAA |  |  |  |  |  |  |  |  |  |  |  |  |
| 5,12-O_2_NAC |  |  |  |  |  |  |  |  |  |  |  |  |
| **Σ11OPAHs** | 0.09 | 0.30 | 0.30 | 0.06 | 0.25 | 0.23 | 0.26 | 0.32 | -0.08 | -0.06 | -0.24 | 0.12 |

Table S22: Results of the Spearman correlation analysis (r) at the Brno traffic site in summer between the individual concentrations of NOPAHs and different meteorological parameters (WD = wind direction, WS = wind speed, RH = relative humidity, T = temperature) or air pollutants. Numbers in bold indicate cases significant at the 95% confidence level, while those underlined are significant at the 99% confidence level

|  | PM_2.5_ | Cu | Zn | OC | EC |
| --- | --- | --- | --- | --- | --- |
| 1-NNAP |  |  |  |  |  |
| 2-NNAP | 0.28 | 0.31 | 0.44 | -0.04 | **0.69** |
| 9-NANT |  |  |  |  |  |
| 3-NPHE | **0.56** | 0.22 | **0.50** | **0.37** | **0.83** |
| 2-NFLT | **0.48** | -0.01 | 0.27 | **0.52** | **0.43** |
| 3-NFLT | **0.48** | 0.25 | **0.46** | **0.55** | **0.45** |
| 1-NPYR | **0.41** | 0.15 | **0.48** | 0.16 | **0.76** |
| 2-NPYR | **0.57** | 0.37 | **0.49** | **0.53** | **0.59** |
| 7-NBAA | **0.56** | 0.37 | 0.39 | **0.43** | 0.33 |
| **Σ9NPAHs** | **0.55** | 0.21 | **0.44** | **0.45** | **0.69** |
| 1,4-O_2_NAP | **0.56** | **0.54** | **0.45** | 0.33 | 0.29 |
| 1(CHO)NAP | 0.51 | 0.35 | 0.48 | 0.31 | 0.41 |
| 9-OFLN |  |  |  |  |  |
| 9,10-O_2_ANT | 0.32 | 0.22 | **0.51** | -0.03 | **0.64** |
| 1,4-O_2_ANT |  |  |  |  |  |
| 9,10-O_2_PHE | 0.03 | 0.36 | 0.28 | 0.04 | 0.28 |
| BaOFLN | **0.53** | 0.30 | **0.68** | 0.28 | **0.78** |
| BbOFLN | **0.57** | 0.35 | **0.67** | **0.36** | **0.72** |
| BAN | **0.48** | 0.35 | **0.61** | 0.24 | **0.68** |
| 7,12-O_2_BAA | **0.52** | 0.27 | **0.60** | **0.46** | **0.49** |
| 5,12-O_2_NAC |  |  |  |  |  |
| **Σ11OPAHs** | **0.44** | 0.32 | **0.58** | 0.23 | **0.77** |

Table S23: Results of the Spearman correlation analysis (r) at the Ljubljana urban background site in summer between the individual concentrations of NOPAHs and different meteorological parameters (WD = wind direction, WS = wind speed, RH = relative humidity, T = temperature) or air pollutants. Numbers in bold indicate cases significant at the 95% confidence level, while those underlined are significant at the 99% confidence level

|  | WS | RH | T | SO_2_ | PM_2.5_ | O_3_ | NO_2_ | CO | Cu | Zn | OC | EC |
| --- | --- | --- | --- | --- | --- | --- | --- | --- | --- | --- | --- | --- |
| 1-NNAP |  |  |  |  |  |  |  |  |  |  |  |  |
| 2-NNAP |  |  |  |  |  |  |  |  |  |  |  |  |
| 9-NANT |  |  |  |  |  |  |  |  |  |  |  |  |
| 3-NPHE |  |  |  |  |  |  |  |  |  |  |  |  |
| 2-NFLT |  |  |  |  |  |  |  |  |  |  |  |  |
| 3-NFLT |  |  |  |  |  |  |  |  |  |  |  |  |
| 1-NPYR | 0.21 | 0.67 | 0.05 | 0.74 | 0.61 | 0.15 | 0.56 | 0.46 | 0.03 | -0.31 | -0.13 | **0.80** |
| 2-NPYR |  |  |  |  |  |  |  |  |  |  |  |  |
| 7-NBAA |  |  |  |  |  |  |  |  |  |  |  |  |
| **Σ9NPAHs** | 0.16 | -0.21 | 0.01 | 0.15 | -0.09 | 0.02 | -0.05 | 0.11 | 0.08 | 0.02 | -0.07 | -0.04 |
| 1,4-O_2_NAP | 0.20 | 0.40 | -0.80 | -0.20 | -0.40 | -0.80 | 0.40 | 0.00 |  | -1.00 | -0.80 | -0.40 |
| 1(CHO)NAP |  |  |  |  |  |  |  |  |  |  |  |  |
| 9-OFLN |  |  |  |  |  |  |  |  |  |  |  |  |
| 9,10-O_2_ANT |  |  |  |  |  |  |  |  |  |  |  |  |
| 1,4-O_2_ANT |  |  |  |  |  |  |  |  |  |  |  |  |
| 9,10-O_2_PHE |  |  |  |  |  |  |  |  |  |  |  |  |
| BaOFLN |  |  |  |  |  |  |  |  |  |  |  |  |
| BbOFLN |  |  |  |  |  |  |  |  |  |  |  |  |
| BAN |  |  |  |  |  |  |  |  |  |  |  |  |
| 7,12-O_2_BAA |  |  |  |  |  |  |  |  |  |  |  |  |
| 5,12-O_2_NAC |  |  |  |  |  |  |  |  |  |  |  |  |
| **Σ11OPAHs** | -0.09 | 0.23 | -0.35 | 0.17 | -0.30 | **-0.47** | 0.15 | -0.33 | 0.13 | **-0.53** | -0.22 | -0.16 |

Table S24: Results of the Spearman correlation analysis (r) at the Ljubljana traffic site in summer between the individual concentrations of NOPAHs and different meteorological parameters (WD = wind direction, WS = wind speed, RH = relative humidity, T = temperature) or air pollutants. Numbers in bold indicate cases significant at the 95% confidence level, while those underlined are significant at the 99% confidence level

|  | WS | RH | T | SO_2_ | PM_2.5_ | NO_2_ | Cu | Zn | OC | EC |
| --- | --- | --- | --- | --- | --- | --- | --- | --- | --- | --- |
| 1-NNAP |  |  |  |  |  |  |  |  |  |  |
| 2-NNAP |  |  |  |  |  |  |  |  |  |  |
| 9-NANT | -0.29 | 0.31 | 0.09 | 0.65 | 0.66 | 0.77 | 0.60 | 0.60 | 0.54 | **0.90** |
| 3-NPHE | -0.10 | 0.23 | -0.27 | 0.14 | 0.15 | 0.16 | 0.10 | 0.26 | 0.25 | **0.74** |
| 2-NFLT | -0.31 | -0.03 | -0.03 | 0.27 | 0.19 | **0.49** | 0.22 | 0.21 | 0.36 | **0.76** |
| 3-NFLT | -0.02 | 0.06 | -0.15 | 0.08 | -0.03 | **0.44** | 0.12 | 0.01 | 0.27 | **0.82** |
| 1-NPYR | **-0.43** | 0.17 | **-0.48** | 0.25 | 0.08 | 0.27 | 0.05 | 0.19 | -0.13 | **0.58** |
| 2-NPYR | -0.23 | 0.13 | -0.33 | -0.04 | -0.09 | 0.33 | 0.41 | 0.34 | 0.15 | **0.90** |
| 7-NBAA | 0.05 | 0.01 | -0.09 | -0.19 | 0.18 | 0.55 | **0.73** | 0.57 | -0.02 | 0.52 |
| **Σ9NPAHs** | -0.16 | 0.03 | -0.24 | -0.06 | -0.04 | 0.38 | **0.48** | **0.48** | 0.17 | **0.80** |
| 1,4-O_2_NAP |  |  |  |  |  |  |  |  |  |  |
| 1(CHO)NAP | -0.80 | 0.40 | -0.80 | -0.63 | -0.80 | 0.20 | 0.80 | 0.80 | 0.40 | 0.80 |
| 9-OFLN |  |  |  |  |  |  |  |  |  |  |
| 9,10-O_2_ANT |  |  |  |  |  |  |  |  |  |  |
| 1,4-O_2_ANT |  |  |  |  |  |  |  |  |  |  |
| 9,10-O_2_PHE |  |  |  |  |  |  |  |  |  |  |
| BaOFLN |  |  |  |  |  |  |  |  |  |  |
| BbOFLN |  |  |  |  |  |  |  |  |  |  |
| BAN | -0.50 | -1.00 | 1.00 | -0.50 | 0.50 | 1.00 | 0.50 | -0.50 | 1.00 | 0.50 |
| 7,12-O_2_BAA |  |  |  |  |  |  |  |  |  |  |
| 5,12-O_2_NAC | -0.24 | 0.45 | 0.26 | -0.02 | 0.33 | -0.17 | -0.05 | -0.26 | 0.05 | 0.24 |
| **Σ11OPAHs** | -0.04 | -0.02 | -0.22 | 0.09 | 0.18 | **0.48** | **0.50** | **0.65** | 0.16 | **0.67** |

Table S25: Pearson correlations between individual NOPAHs at the rural site in winter. Numbers in bold indicate cases significant at the 95% confidence level, while those underlined are significant at the 99% confidence level

|  | 1-NNAP | 2-NNAP | 9-NANT | 3-NPHE | 2-NFLT | 3-NFLT | 1-NPYR | 2-NPYR | 7-NBAA | **Σ9NPAHs** | 1,4-O_2_NAP | 1(CHO)NAP | 9-OFLN | 9,10-O_2_ANT | 1,4-O_2_ANT | 9,10-O_2_PHE | BaOFLN | BbOFLN | BAN | 7,12-O_2_BAA | 5,12-O_2_NAC | **Σ11OPAHs** |
| --- | --- | --- | --- | --- | --- | --- | --- | --- | --- | --- | --- | --- | --- | --- | --- | --- | --- | --- | --- | --- | --- | --- |
| 1-NNAP | 1.00 |  | 1.00 |  | 1.00 | 1.00 | 1.00 | 1.00 | 1.00 | 1.00 |  |  |  | 1.00 | 1.00 | 1.00 | 0.80 | 1.00 | 1.00 | 1.00 | 1.00 | 1.00 |
| 2-NNAP |  |  |  |  |  |  |  |  |  |  |  |  |  |  |  |  |  |  |  |  |  |  |
| 9-NANT | 1.00 |  | **1.00** | 1.00 | **0.83** | **0.79** | **0.79** | **0.93** | **0.96** | **0.98** | -0.20 | 0.50 |  | **0.81** | 0.66 | **0.96** | **0.83** | **0.86** | **0.93** | **0.86** | **0.86** | **0.93** |
| 3-NPHE |  |  | 1.00 | 1.00 | 0.80 | 0.40 | 1.00 | 1.00 | 1.00 | 0.80 | 1.00 |  |  | 0.80 | 1.00 | 1.00 | 1.00 | 1.00 | 1.00 | 1.00 | 1.00 | 1.00 |
| 2-NFLT | 1.00 |  | **0.83** | 0.80 | **1.00** | **0.86** | **0.96** | **0.95** | 0.61 | **0.86** | 0.80 | 0.50 |  | **0.93** | **0.94** | **0.82** | **0.95** | **0.98** | **0.95** | **0.98** | **0.98** | **0.95** |
| 3-NFLT | 1.00 |  | **0.79** | 0.40 | **0.86** | **1.00** | 0.75 | **0.82** | 0.75 | **0.89** | 0.00 | 1.00 |  | 0.68 | 0.60 | 0.75 | 0.71 | 0.75 | **0.82** | 0.75 | 0.75 | 0.82 |
| 1-NPYR | 1.00 |  | **0.79** | 1.00 | **0.96** | 0.75 | **1.00** | **0.96** | 0.64 | 0.75 | 1.00 | 0.50 |  | **0.96** | **1.00** | **0.89** | **0.96** | **1.00** | **0.96** | **1.00** | **1.00** | **0.96** |
| 2-NPYR | 1.00 |  | **0.93** | 1.00 | **0.95** | **0.82** | **0.96** | **1.00** | **0.79** | **0.90** | 0.80 | 1.00 |  | **0.95** | **0.94** | **0.96** | **0.95** | **0.98** | **1.00** | **0.98** | **0.98** | **1.00** |
| 7-NBAA | 1.00 |  | **0.96** | 1.00 | 0.61 | 0.75 | 0.64 | **0.79** | **1.00** | **0.93** | -0.20 | 0.50 |  | 0.61 | 0.43 | **0.89** | 0.61 | 0.64 | **0.79** | 0.64 | 0.64 | 0.79 |
| **Σ9NPAHs** | 1.00 |  | **0.98** | 0.80 | **0.86** | **0.89** | 0.75 | **0.90** | **0.93** | **1.00** | -0.40 | 0.50 |  | **0.76** | 0.60 | **0.89** | **0.81** | **0.83** | **0.90** | **0.83** | **0.83** | **0.90** |
| 1,4-O2NAP |  |  | -0.20 | 1.00 | 0.80 | 0.00 | 1.00 | 0.80 | -0.20 | -0.40 | 1.00 | 0.50 |  | 1.00 | 1.00 | 0.40 | 1.00 | 1.00 | 0.80 | 1.00 | 1.00 | 0.80 |
| 1(CHO)NAP |  |  | 0.50 |  | 0.50 | 1.00 | 0.50 | 1.00 | 0.50 | 0.50 | 0.50 | 1.00 |  | 0.50 | 0.50 | 0.50 | 0.50 | 0.50 | 1.00 | 0.50 | 0.50 | 1.00 |
| 9-OFLN |  |  |  |  |  |  |  |  |  |  |  |  |  |  |  |  |  |  |  |  |  |  |
| 9,10-O2ANT | 1.00 |  | **0.81** | 0.80 | **0.93** | 0.68 | **0.96** | **0.95** | 0.61 | **0.76** | 1.00 | 0.50 |  | **1.00** | **0.94** | **0.86** | **0.95** | **0.98** | **0.95** | **0.98** | **0.98** | **0.95** |
| 1,4-O2ANT | 1.00 |  | 0.66 | 1.00 | **0.94** | 0.60 | **1.00** | **0.94** | 0.43 | 0.60 | 1.00 | 0.50 |  | **0.94** | **1.00** | 0.83 | **1.00** | **1.00** | **0.94** | **1.00** | **1.00** | **0.94** |
| 9,10-O2PHE | 1.00 |  | **0.96** | 1.00 | **0.82** | 0.75 | **0.89** | **0.96** | **0.89** | **0.89** | 0.40 | 0.50 |  | **0.86** | 0.83 | **1.00** | **0.86** | **0.89** | **0.96** | **0.89** | **0.89** | **0.96** |
| BaOFLN | 0.80 |  | **0.83** | 1.00 | **0.95** | 0.71 | **0.96** | **0.95** | 0.61 | **0.81** | 1.00 | 0.50 |  | **0.95** | **1.00** | **0.86** | **1.00** | **0.98** | **0.95** | **0.98** | **0.98** | **0.95** |
| BbOFLN | 1.00 |  | **0.86** | 1.00 | **0.98** | 0.75 | **1.00** | **0.98** | 0.64 | **0.83** | 1.00 | 0.50 |  | **0.98** | **1.00** | **0.89** | **0.98** | **1.00** | **0.98** | **1.00** | **1.00** | **0.98** |
| BAN | 1.00 |  | **0.93** | 1.00 | **0.95** | **0.82** | **0.96** | **1.00** | **0.79** | **0.90** | 0.80 | 1.00 |  | **0.95** | **0.94** | **0.96** | **0.95** | **0.98** | **1.00** | **0.98** | **0.98** | **1.00** |
| 7,12-O2BAA | 1.00 |  | **0.86** | 1.00 | **0.98** | 0.75 | **1.00** | **0.98** | 0.64 | **0.83** | 1.00 | 0.50 |  | **0.98** | **1.00** | **0.89** | **0.98** | **1.00** | **0.98** | **1.00** | **1.00** | **0.98** |
| 5,12-O2NAC | 1.00 |  | **0.86** | 1.00 | **0.98** | 0.75 | **1.00** | **0.98** | 0.64 | **0.83** | 1.00 | 0.50 |  | **0.98** | **1.00** | **0.89** | **0.98** | **1.00** | **0.98** | **1.00** | **1.00** | **0.98** |
| **Σ11OPAHs** | 1.00 |  | **0.93** | 1.00 | **0.95** | **0.82** | **0.96** | **1.00** | **0.79** | **0.90** | 0.80 | 1.00 |  | **0.95** | **0.94** | **0.96** | **0.95** | **0.98** | **1.00** | **0.98** | **0.98** | **1.00** |

Table S26: Pearson correlations between individual NOPAHs at the Brno urban background site in winter. Numbers in bold indicate cases significant at the 95% confidence level, while those underlined are significant at the 99% confidence level

|  | 1-NNAP | 2-NNAP | 9-NANT | 3-NPHE | 2-NFLT | 3-NFLT | 1-NPYR | 2-NPYR | 7-NBAA | **Σ9NPAHs** | 1,4-O_2_NAP | 1(CHO)NAP | 9-OFLN | 9,10-O_2_ANT | 1,4-O_2_ANT | 9,10-O_2_PHE | BaOFLN | BbOFLN | BAN | 7,12-O_2_BAA | 5,12-O_2_NAC | **Σ11OPAHs** |
| --- | --- | --- | --- | --- | --- | --- | --- | --- | --- | --- | --- | --- | --- | --- | --- | --- | --- | --- | --- | --- | --- | --- |
| 1-NNAP | **1.00** |  | 0.68 |  | **0.87** |  | **0.80** | **0.99** |  | **0.96** |  |  |  |  |  |  | **0.89** | **0.78** | **0.94** | **0.89** | **0.89** | **0.94** |
| 2-NNAP |  |  |  |  |  |  |  |  |  |  |  |  |  |  |  |  |  |  |  |  |  |  |
| 9-NANT | 0.68 |  | **1.00** |  | **0.78** | **0.96** | **0.79** | **0.79** | **0.95** | **0.88** | **0.76** | 0.43 |  | **0.77** |  |  | **0.80** | **0.80** | **0.80** | **0.79** | **0.80** | **0.82** |
| 3-NPHE |  |  |  |  |  |  |  |  |  |  |  |  |  |  |  |  |  |  |  |  |  |  |
| 2-NFLT | **0.87** |  | **0.78** |  | **1.00** | **0.98** | **0.98** | **0.95** | **0.95** | **0.97** | **0.81** | 0.46 |  | **0.92** |  |  | **0.98** | **0.96** | **0.98** | **0.98** | **0.97** | **0.97** |
| 3-NFLT |  |  | **0.96** |  | **0.98** | **1.00** | **0.95** | **0.90** | **0.98** | **0.98** | **0.76** | 0.36 |  | **0.93** |  |  | **0.95** | **0.94** | **0.93** | **0.93** | **0.81** | **0.92** |
| 1-NPYR | **0.80** |  | **0.79** |  | **0.98** | **0.95** | **1.00** | **0.96** | **0.97** | **0.97** | **0.75** | 0.46 |  | **0.96** |  |  | **0.97** | **0.96** | **0.97** | **0.97** | **0.97** | **0.97** |
| 2-NPYR | **0.99** |  | **0.79** |  | **0.95** | **0.90** | **0.96** | **1.00** | **0.90** | **0.96** | **0.93** | 0.57 |  | **0.83** |  |  | **0.95** | **0.93** | **0.95** | **0.95** | **0.94** | **0.95** |
| 7-NBAA |  |  | **0.95** |  | **0.95** | **0.98** | **0.97** | **0.90** | **1.00** | **0.98** | **0.75** | 0.39 |  | **0.95** |  |  | **0.95** | **0.95** | **0.95** | **0.94** | **0.84** | **0.95** |
| **Σ9NPAHs** | **0.96** |  | **0.88** |  | **0.97** | **0.98** | **0.97** | **0.96** | **0.98** | **1.00** | **0.76** | 0.39 |  | **0.89** |  |  | **0.98** | **0.97** | **0.98** | **0.98** | **0.97** | **0.98** |
| 1,4-O2NAP |  |  | **0.76** |  | **0.81** | **0.76** | **0.75** | **0.93** | **0.75** | **0.76** | **1.00** | **0.93** |  | **0.76** |  |  | **0.75** | **0.75** | **0.81** | **0.75** | **0.70** | **0.75** |
| 1(CHO)NAP |  |  | 0.43 |  | 0.46 | 0.36 | 0.46 | 0.57 | 0.39 | 0.39 | **0.93** | **1.00** |  | 0.61 |  |  | 0.57 | 0.54 | 0.57 | 0.57 | 0.64 | 0.61 |
| 9-OFLN |  |  |  |  |  |  |  |  |  |  |  |  |  |  |  |  |  |  |  |  |  |  |
| 9,10-O2ANT |  |  | **0.77** |  | **0.92** | **0.93** | **0.96** | **0.83** | **0.95** | **0.89** | **0.76** | 0.61 |  | **1.00** |  |  | **0.99** | **0.98** | **0.98** | **0.99** | **0.96** | **0.99** |
| 1,4-O2ANT |  |  |  |  |  |  |  |  |  |  |  |  |  |  |  |  |  |  |  |  |  |  |
| 9,10-O2PHE |  |  |  |  |  |  |  |  |  |  |  |  |  |  |  |  |  |  |  |  |  |  |
| BaOFLN | **0.89** |  | **0.80** |  | **0.98** | **0.95** | **0.97** | **0.95** | **0.95** | **0.98** | **0.75** | 0.57 |  | **0.99** |  |  | **1.00** | **1.00** | **1.00** | **1.00** | **0.99** | **1.00** |
| BbOFLN | **0.78** |  | **0.80** |  | **0.96** | **0.94** | **0.96** | **0.93** | **0.95** | **0.97** | **0.75** | 0.54 |  | **0.98** |  |  | **1.00** | **1.00** | **1.00** | **1.00** | **0.99** | **1.00** |
| BAN | **0.94** |  | **0.80** |  | **0.98** | **0.93** | **0.97** | **0.95** | **0.95** | **0.98** | **0.81** | 0.57 |  | **0.98** |  |  | **1.00** | **1.00** | **1.00** | **1.00** | **0.99** | **1.00** |
| 7,12-O2BAA | **0.89** |  | **0.79** |  | **0.98** | **0.93** | **0.97** | **0.95** | **0.94** | **0.98** | **0.75** | 0.57 |  | **0.99** |  |  | **1.00** | **1.00** | **1.00** | **1.00** | **0.99** | **1.00** |
| 5,12-O2NAC | **0.89** |  | **0.80** |  | **0.97** | **0.81** | **0.97** | **0.94** | **0.84** | **0.97** | **0.70** | 0.64 |  | **0.96** |  |  | **0.99** | **0.99** | **0.99** | **0.99** | **1.00** | **0.99** |
| **Σ11OPAHs** | **0.94** |  | **0.82** |  | **0.97** | **0.92** | **0.97** | **0.95** | **0.95** | **0.98** | **0.75** | 0.61 |  | **0.99** |  |  | **1.00** | **1.00** | **1.00** | **1.00** | **0.99** | **1.00** |

Table S27: Pearson correlations between individual NOPAHs at the Brno traffic site in winter. Numbers in bold indicate cases significant at the 95% confidence level, while those underlined are significant at the 99% confidence level

|  | 1-NNAP | 2-NNAP | 9-NANT | 3-NPHE | 2-NFLT | 3-NFLT | 1-NPYR | 2-NPYR | 7-NBAA | **Σ9NPAHs** | 1,4-O_2_NAP | 1(CHO)NAP | 9-OFLN | 9,10-O_2_ANT | 1,4-O_2_ANT | 9,10-O_2_PHE | BaOFLN | BbOFLN | BAN | 7,12-O_2_BAA | 5,12-O_2_NAC | **Σ11OPAHs** |
| --- | --- | --- | --- | --- | --- | --- | --- | --- | --- | --- | --- | --- | --- | --- | --- | --- | --- | --- | --- | --- | --- | --- |
| 1-NNAP | **1.00** | **0.96** | **0.88** | **0.94** | **0.90** | **0.90** | **0.93** | **0.91** | **0.87** | **0.92** | **0.81** | **0.86** | 0.48 | **0.93** | **0.78** | **0.76** | **0.92** | **0.92** | **0.94** | **0.92** | **0.93** | **0.93** |
| 2-NNAP | **0.96** | **1.00** | **0.88** | **0.94** | **0.90** | **0.91** | **0.96** | **0.93** | **0.87** | **0.93** | **0.84** | **0.87** | **0.62** | **0.92** | **0.85** | **0.81** | **0.90** | **0.94** | **0.94** | **0.93** | **0.94** | **0.93** |
| 9-NANT | **0.88** | **0.88** | **1.00** | **0.96** | **0.93** | **0.88** | **0.89** | **0.93** | **0.93** | **0.98** | **0.75** | **0.84** | **0.79** | **0.95** | **0.85** | **0.93** | **0.93** | **0.91** | **0.94** | **0.96** | **0.95** | **0.95** |
| 3-NPHE | **0.94** | **0.94** | **0.96** | **1.00** | **0.94** | **0.92** | **0.92** | **0.94** | **0.96** | **0.97** | **0.84** | **0.92** | **0.70** | **0.96** | **0.78** | **0.89** | **0.95** | **0.94** | **0.96** | **0.96** | **0.96** | **0.96** |
| 2-NFLT | **0.90** | **0.90** | **0.93** | **0.94** | **1.00** | **0.99** | **0.93** | **0.97** | **0.92** | **0.96** | **0.95** | **0.91** | **0.85** | **0.93** | **0.67** | **0.81** | **0.97** | **0.96** | **0.96** | **0.97** | **0.97** | **0.97** |
| 3-NFLT | **0.90** | **0.91** | **0.88** | **0.92** | **0.99** | **1.00** | **0.97** | **0.99** | **0.93** | **0.96** | **0.94** | **0.91** | **0.84** | **0.96** | **0.82** | **0.75** | **0.98** | **0.93** | **0.97** | **0.98** | **0.97** | **0.97** |
| 1-NPYR | **0.93** | **0.96** | **0.89** | **0.92** | **0.93** | **0.97** | **1.00** | **0.97** | **0.88** | **0.94** | **0.91** | **0.90** | **0.89** | **0.93** | **0.81** | **0.85** | **0.92** | **0.94** | **0.98** | **0.96** | **0.96** | **0.96** |
| 2-NPYR | **0.91** | **0.93** | **0.93** | **0.94** | **0.97** | **0.99** | **0.97** | **1.00** | **0.89** | **0.97** | **0.87** | **0.86** | **0.75** | **0.93** | **0.88** | **0.91** | **0.94** | **0.95** | **0.97** | **0.98** | **0.98** | **0.97** |
| 7-NBAA | **0.87** | **0.87** | **0.93** | **0.96** | **0.92** | **0.93** | **0.88** | **0.89** | **1.00** | **0.97** | **0.90** | **0.93** | **0.73** | **0.94** | 0.32 | **0.86** | **0.92** | **0.85** | **0.91** | **0.93** | **0.92** | **0.91** |
| **Σ9NPAHs** | **0.92** | **0.93** | **0.98** | **0.97** | **0.96** | **0.96** | **0.94** | **0.97** | **0.97** | **1.00** | **0.92** | **0.93** | **0.80** | **0.97** | **0.90** | **0.96** | **0.96** | **0.95** | **0.97** | **0.99** | **0.98** | **0.98** |
| 1,4-O2NAP | **0.81** | **0.84** | **0.75** | **0.84** | **0.95** | **0.94** | **0.91** | **0.87** | **0.90** | **0.92** | **1.00** | **0.97** | **0.98** | **1.00** | 0.00 | 1.00 | **0.98** | **0.88** | **0.95** | **0.98** | **0.99** | **0.99** |
| 1(CHO)NAP | **0.86** | **0.87** | **0.84** | **0.92** | **0.91** | **0.91** | **0.90** | **0.86** | **0.93** | **0.93** | **0.97** | **1.00** | **0.92** | **0.94** | 0.12 | 0.31 | **0.92** | **0.84** | **0.93** | **0.93** | **0.92** | **0.92** |
| 9-OFLN | 0.48 | **0.62** | **0.79** | **0.70** | **0.85** | **0.84** | **0.89** | **0.75** | **0.73** | **0.80** | **0.98** | **0.92** | **1.00** | **0.99** |  |  | **0.96** | **0.80** | **0.92** | **0.95** | **0.97** | **1.00** |
| 9,10-O2ANT | **0.93** | **0.92** | **0.95** | **0.96** | **0.93** | **0.96** | **0.93** | **0.93** | **0.94** | **0.97** | **1.00** | **0.94** | **0.99** | **1.00** | **0.88** | **0.88** | **0.98** | **0.96** | **0.97** | **0.97** | **0.97** | **0.98** |
| 1,4-O2ANT | **0.78** | **0.85** | **0.85** | **0.78** | **0.67** | **0.82** | **0.81** | **0.88** | 0.32 | **0.90** | 0.00 | 0.12 |  | **0.88** | **1.00** | **0.87** | **0.77** | **0.87** | **0.89** | **0.89** | **0.95** | **0.92** |
| 9,10-O2PHE | **0.76** | **0.81** | **0.93** | **0.89** | **0.81** | **0.75** | **0.85** | **0.91** | **0.86** | **0.96** | 1.00 | 0.31 |  | **0.88** | **0.87** | **1.00** | **0.79** | **0.88** | **0.90** | **0.95** | **0.95** | **0.94** |
| BaOFLN | **0.92** | **0.90** | **0.93** | **0.95** | **0.97** | **0.98** | **0.92** | **0.94** | **0.92** | **0.96** | **0.98** | **0.92** | **0.96** | **0.98** | **0.77** | **0.79** | **1.00** | **0.97** | **0.96** | **0.97** | **0.96** | **0.98** |
| BbOFLN | **0.92** | **0.94** | **0.91** | **0.94** | **0.96** | **0.93** | **0.94** | **0.95** | **0.85** | **0.95** | **0.88** | **0.84** | **0.80** | **0.96** | **0.87** | **0.88** | **0.97** | **1.00** | **0.96** | **0.97** | **0.98** | **0.98** |
| BAN | **0.94** | **0.94** | **0.94** | **0.96** | **0.96** | **0.97** | **0.98** | **0.97** | **0.91** | **0.97** | **0.95** | **0.93** | **0.92** | **0.97** | **0.89** | **0.90** | **0.96** | **0.96** | **1.00** | **0.98** | **0.98** | **0.99** |
| 7,12-O2BAA | **0.92** | **0.93** | **0.96** | **0.96** | **0.97** | **0.98** | **0.96** | **0.98** | **0.93** | **0.99** | **0.98** | **0.93** | **0.95** | **0.97** | **0.89** | **0.95** | **0.97** | **0.97** | **0.98** | **1.00** | **0.99** | **0.99** |
| 5,12-O2NAC | **0.93** | **0.94** | **0.95** | **0.96** | **0.97** | **0.97** | **0.96** | **0.98** | **0.92** | **0.98** | **0.99** | **0.92** | **0.97** | **0.97** | **0.95** | **0.95** | **0.96** | **0.98** | **0.98** | **0.99** | **1.00** | **0.99** |
| **Σ11OPAHs** | **0.93** | **0.93** | **0.95** | **0.96** | **0.97** | **0.97** | **0.96** | **0.97** | **0.91** | **0.98** | **0.99** | **0.92** | **1.00** | **0.98** | **0.92** | **0.94** | **0.98** | **0.98** | **0.99** | **0.99** | **0.99** | **1.00** |

Table S28: Pearson correlations between individual NOPAHs at the Ljubljana urban background site in winter. Numbers in bold indicate cases significant at the 95% confidence level, while those underlined are significant at the 99% confidence level

|  | 1-NNAP | 2-NNAP | 9-NANT | 3-NPHE | 2-NFLT | 3-NFLT | 1-NPYR | 2-NPYR | 7-NBAA | **Σ9NPAHs** | 1,4-O_2_NAP | 1(CHO)NAP | 9-OFLN | 9,10-O_2_ANT | 1,4-O_2_ANT | 9,10-O_2_PHE | BaOFLN | BbOFLN | BAN | 7,12-O_2_BAA | 5,12-O_2_NAC | **Σ11OPAHs** |
| --- | --- | --- | --- | --- | --- | --- | --- | --- | --- | --- | --- | --- | --- | --- | --- | --- | --- | --- | --- | --- | --- | --- |
| 1-NNAP | **1.00** |  | 0.38 | 0.50 | 0.52 | 0.48 | 0.55 | 0.43 | 0.40 | 0.38 | 0.02 | 0.71 | 0.45 | 0.70 |  |  | 0.68 |  | 0.48 | 0.55 | 0.48 | 0.48 |
| 2-NNAP |  |  |  |  |  |  |  |  |  |  |  |  |  |  |  |  |  |  |  |  |  |  |
| 9-NANT | 0.38 |  | **1.00** | 0.90 | **0.84** | **0.77** | **0.89** | **0.96** | **0.94** | **0.92** | 0.44 | 0.08 | **0.53** | **0.81** |  |  | **0.77** | 0.49 | **0.89** | **0.98** | **0.91** | **0.94** |
| 3-NPHE | 0.50 |  | 0.90 | **1.00** | 0.70 | 0.60 | 0.10 | 0.90 | 0.70 | 0.90 | 0.30 | 0.70 | 0.50 | 0.50 |  |  | 0.50 |  | 0.60 | 0.90 | 0.30 | 0.60 |
| 2-NFLT | 0.52 |  | **0.84** | 0.70 | **1.00** | **0.91** | **0.86** | **0.92** | **0.89** | **0.97** | **0.67** | 0.40 | **0.62** | **0.64** |  |  | **0.70** | **0.81** | **0.93** | **0.89** | **0.93** | **0.94** |
| 3-NFLT | 0.48 |  | **0.77** | 0.60 | **0.91** | **1.00** | **0.78** | **0.86** | **0.90** | **0.92** | **0.82** | **0.70** | **0.78** | 0.45 |  |  | 0.47 | 0.50 | **0.88** | **0.78** | **0.84** | **0.85** |
| 1-NPYR | 0.55 |  | **0.89** | 0.10 | **0.86** | **0.78** | **1.00** | **0.88** | **0.79** | **0.87** | **0.53** | 0.19 | **0.60** | 0.52 |  |  | **0.58** | 0.00 | **0.86** | **0.88** | **0.87** | **0.90** |
| 2-NPYR | 0.43 |  | **0.96** | 0.90 | **0.92** | **0.86** | **0.88** | **1.00** | **0.89** | **0.94** | **0.62** | 0.21 | **0.59** | **0.85** |  |  | **0.78** | 0.60 | **0.93** | **0.93** | **0.93** | **0.95** |
| 7-NBAA | 0.40 |  | **0.94** | 0.70 | **0.89** | **0.90** | **0.79** | **0.89** | **1.00** | **0.96** | **0.82** | **0.89** | **0.87** | **0.89** |  |  | **0.88** |  | **0.93** | **0.89** | **0.95** | **0.93** |
| **Σ9NPAHs** | 0.38 |  | **0.92** | 0.90 | **0.97** | **0.92** | **0.87** | **0.94** | **0.96** | **1.00** | **0.73** | **0.46** | **0.70** | **0.84** |  |  | **0.78** | **0.79** | **0.96** | **0.90** | **0.95** | **0.96** |
| 1,4-O2NAP | 0.02 |  | 0.44 | 0.30 | **0.67** | **0.82** | **0.53** | **0.62** | **0.82** | **0.73** | **1.00** | **0.76** | **0.75** | 0.15 |  |  | -0.05 | 0.50 | **0.71** | **0.48** | **0.67** | **0.66** |
| 1(CHO)NAP | 0.71 |  | 0.08 | 0.70 | 0.40 | **0.70** | 0.19 | 0.21 | **0.89** | **0.46** | **0.76** | **1.00** | **0.88** | 0.14 |  |  | 0.22 | 0.20 | **0.44** | 0.19 | 0.32 | 0.33 |
| 9-OFLN | 0.45 |  | **0.53** | 0.50 | **0.62** | **0.78** | **0.60** | **0.59** | **0.87** | **0.70** | **0.75** | **0.88** | **1.00** | 0.25 |  |  | 0.34 |  | **0.72** | **0.59** | **0.62** | **0.68** |
| 9,10-O2ANT | 0.70 |  | **0.81** | 0.50 | **0.64** | 0.45 | 0.52 | **0.85** | **0.89** | **0.84** | 0.15 | 0.14 | 0.25 | **1.00** |  |  | **0.94** | 0.83 | **0.92** | **0.80** | **0.87** | **0.92** |
| 1,4-O2ANT |  |  |  |  |  |  |  |  |  |  |  |  |  |  |  |  |  |  |  |  |  |  |
| 9,10-O2PHE |  |  |  |  |  |  |  |  |  |  |  |  |  |  |  |  |  |  |  |  |  |  |
| BaOFLN | 0.68 |  | **0.77** | 0.50 | **0.70** | 0.47 | **0.58** | **0.78** | **0.88** | **0.78** | -0.05 | 0.22 | 0.34 | **0.94** |  |  | **1.00** | **1.00** | **0.98** | **0.79** | **0.91** | **0.94** |
| BbOFLN |  |  | 0.49 |  | **0.81** | 0.50 | 0.00 | 0.60 |  | **0.79** | 0.50 | 0.20 |  | 0.83 |  |  | **1.00** | **1.00** | **0.98** | **0.83** | **0.95** | **0.95** |
| BAN | 0.48 |  | **0.89** | 0.60 | **0.93** | **0.88** | **0.86** | **0.93** | **0.93** | **0.96** | **0.71** | **0.44** | **0.72** | **0.92** |  |  | **0.98** | **0.98** | **1.00** | **0.89** | **0.97** | **0.98** |
| 7,12-O2BAA | 0.55 |  | **0.98** | 0.90 | **0.89** | **0.78** | **0.88** | **0.93** | **0.89** | **0.90** | **0.48** | 0.19 | **0.59** | **0.80** |  |  | **0.79** | **0.83** | **0.89** | **1.00** | **0.90** | **0.92** |
| 5,12-O2NAC | 0.48 |  | **0.91** | 0.30 | **0.93** | **0.84** | **0.87** | **0.93** | **0.95** | **0.95** | **0.67** | 0.32 | **0.62** | **0.87** |  |  | **0.91** | **0.95** | **0.97** | **0.90** | **1.00** | **0.98** |
| **Σ11OPAHs** | 0.48 |  | **0.94** | 0.60 | **0.94** | **0.85** | **0.90** | **0.95** | **0.93** | **0.96** | **0.66** | 0.33 | **0.68** | **0.92** |  |  | **0.94** | **0.95** | **0.98** | **0.92** | **0.98** | **1.00** |

Table S29: Pearson correlations between individual NOPAHs at the Ljubljana traffic site in winter. Numbers in bold indicate cases significant at the 95% confidence level, while those underlined are significant at the 99% confidence level

|  | 1-NNAP | 2-NNAP | 9-NANT | 3-NPHE | 2-NFLT | 3-NFLT | 1-NPYR | 2-NPYR | 7-NBAA | **Σ9NPAHs** | 1,4-O_2_NAP | 1(CHO)NAP | 9-OFLN | 9,10-O_2_ANT | 1,4-O_2_ANT | 9,10-O_2_PHE | BaOFLN | BbOFLN | BAN | 7,12-O_2_BAA | 5,12-O_2_NAC | **Σ11OPAHs** |
| --- | --- | --- | --- | --- | --- | --- | --- | --- | --- | --- | --- | --- | --- | --- | --- | --- | --- | --- | --- | --- | --- | --- |
| 1-NNAP | **1.00** |  | **0.72** |  | 0.43 | 0.53 | 0.34 | **0.59** | 0.45 | 0.43 | 0.47 | **0.62** | **0.68** | **0.93** |  |  | 0.19 |  | 0.53 | 0.34 | 0.45 | 0.49 |
| 2-NNAP |  |  |  |  |  |  |  |  |  |  |  |  |  |  |  |  |  |  |  |  |  |  |
| 9-NANT | **0.72** |  | **1.00** |  | **0.71** | **0.69** | **0.81** | **0.89** | **0.97** | **0.91** | **0.63** | 0.15 | 0.37 | **0.80** |  |  | **0.76** | **1.00** | **0.79** | **0.94** | **0.83** | **0.87** |
| 3-NPHE |  |  |  |  |  |  |  |  |  |  |  |  |  |  |  |  |  |  |  |  |  |  |
| 2-NFLT | 0.43 |  | **0.71** |  | **1.00** | **0.97** | **0.92** | **0.91** | **0.87** | **0.97** | **0.77** | **0.49** | **0.54** | **0.75** |  |  | **0.86** | **0.90** | **0.91** | **0.82** | **0.87** | **0.90** |
| 3-NFLT | 0.53 |  | **0.69** |  | **0.97** | **1.00** | **0.85** | **0.90** | **0.84** | **0.92** | **0.82** | **0.58** | 0.48 | 0.38 |  |  | **0.75** | 1.00 | **0.88** | **0.83** | **0.86** | **0.89** |
| 1-NPYR | 0.34 |  | **0.81** |  | **0.92** | **0.85** | **1.00** | **0.91** | **0.88** | **0.95** | **0.69** | 0.38 | 0.40 | **0.89** |  |  | **0.91** | **1.00** | **0.93** | **0.89** | **0.94** | **0.94** |
| 2-NPYR | **0.59** |  | **0.89** |  | **0.91** | **0.90** | **0.91** | **1.00** | **0.90** | **0.93** | **0.77** | 0.40 | 0.46 | **0.92** |  |  | **0.93** | **0.95** | **0.94** | **0.95** | **0.93** | **0.94** |
| 7-NBAA | 0.45 |  | **0.97** |  | **0.87** | **0.84** | **0.88** | **0.90** | **1.00** | **0.96** | **0.59** | **0.63** | **0.57** | **0.94** |  |  | **0.82** |  | **0.92** | **0.94** | **0.96** | **0.91** |
| **Σ9NPAHs** | 0.43 |  | **0.91** |  | **0.97** | **0.92** | **0.95** | **0.93** | **0.96** | **1.00** | **0.70** | 0.42 | 0.40 | **0.94** |  |  | **0.90** | **0.93** | **0.91** | **0.86** | **0.88** | **0.91** |
| 1,4-O2NAP | 0.47 |  | **0.63** |  | **0.77** | **0.82** | **0.69** | **0.77** | **0.59** | **0.70** | **1.00** | **0.73** | **0.67** | 0.10 |  |  | **0.60** |  | **0.64** | **0.54** | **0.66** | **0.74** |
| 1(CHO)NAP | **0.62** |  | 0.15 |  | **0.49** | **0.58** | 0.38 | 0.40 | **0.63** | 0.42 | **0.73** | **1.00** | **0.78** | 0.31 |  |  | 0.21 |  | 0.40 | 0.20 | 0.41 | **0.47** |
| 9-OFLN | **0.68** |  | 0.37 |  | **0.54** | 0.48 | 0.40 | 0.46 | **0.57** | 0.40 | **0.67** | **0.78** | **1.00** | 0.11 |  |  | 0.26 |  | **0.56** | 0.28 | 0.44 | **0.53** |
| 9,10-O2ANT | **0.93** |  | **0.80** |  | **0.75** | 0.38 | **0.89** | **0.92** | **0.94** | **0.94** | 0.10 | 0.31 | 0.11 | **1.00** |  |  | **0.91** | **0.98** | **0.88** | **0.93** | **0.93** | **0.93** |
| 1,4-O2ANT |  |  |  |  |  |  |  |  |  |  |  |  |  |  |  |  |  |  |  |  |  |  |
| 9,10-O2PHE |  |  |  |  |  |  |  |  |  |  |  |  |  |  |  |  |  |  |  |  |  |  |
| BaOFLN | 0.19 |  | **0.76** |  | **0.86** | **0.75** | **0.91** | **0.93** | **0.82** | **0.90** | **0.60** | 0.21 | 0.26 | **0.91** |  |  | **1.00** | **0.98** | **0.95** | **0.96** | **0.94** | **0.96** |
| BbOFLN |  |  | **1.00** |  | **0.90** | 1.00 | **1.00** | **0.95** |  | **0.93** |  |  |  | **0.98** |  |  | **0.98** | **1.00** | **1.00** | **1.00** | **0.98** | **1.00** |
| BAN | 0.53 |  | **0.79** |  | **0.91** | **0.88** | **0.93** | **0.94** | **0.92** | **0.91** | **0.64** | 0.40 | **0.56** | **0.88** |  |  | **0.95** | **1.00** | **1.00** | **0.95** | **0.98** | **0.99** |
| 7,12-O2BAA | 0.34 |  | **0.94** |  | **0.82** | **0.83** | **0.89** | **0.95** | **0.94** | **0.86** | **0.54** | 0.20 | 0.28 | **0.93** |  |  | **0.96** | **1.00** | **0.95** | **1.00** | **0.95** | **0.95** |
| 5,12-O2NAC | 0.45 |  | **0.83** |  | **0.87** | **0.86** | **0.94** | **0.93** | **0.96** | **0.88** | **0.66** | 0.41 | 0.44 | **0.93** |  |  | **0.94** | **0.98** | **0.98** | **0.95** | **1.00** | **0.98** |
| **Σ11OPAHs** | 0.49 |  | **0.87** |  | **0.90** | **0.89** | **0.94** | **0.94** | **0.91** | **0.91** | **0.74** | **0.47** | **0.53** | **0.93** |  |  | **0.96** | **1.00** | **0.99** | **0.95** | **0.98** | **1.00** |

Table S30: Pearson correlations between individual NOPAHs at the Brno traffic site in summer. Numbers in bold indicate cases significant at the 95% confidence level, while those underlined are significant at the 99% confidence level

|  | 1-NNAP | 2-NNAP | 9-NANT | 3-NPHE | 2-NFLT | 3-NFLT | 1-NPYR | 2-NPYR | 7-NBAA | **Σ9NPAHs** | 1,4-O_2_NAP | 1(CHO)NAP | 9-OFLN | 9,10-O_2_ANT | 1,4-O_2_ANT | 9,10-O_2_PHE | BaOFLN | BbOFLN | BAN | 7,12-O_2_BAA | 5,12-O_2_NAC | **Σ11OPAHs** |
| --- | --- | --- | --- | --- | --- | --- | --- | --- | --- | --- | --- | --- | --- | --- | --- | --- | --- | --- | --- | --- | --- | --- |
| 1-NNAP |  |  |  |  |  |  |  |  |  |  |  |  |  |  |  |  |  |  |  |  |  |  |
| 2-NNAP |  | **1.00** |  | **0.56** | 0.22 | 0.16 | **0.49** | 0.39 | 0.28 | **0.53** | **0.62** | **0.59** |  | **0.68** |  | 0.28 | **0.48** | **0.46** | **0.49** | 0.33 |  | **0.58** |
| 9-NANT |  |  |  |  |  |  |  |  |  |  |  |  |  |  |  |  |  |  |  |  |  |  |
| 3-NPHE |  | **0.56** |  | **1.00** | **0.70** | **0.58** | **0.86** | **0.73** | **0.46** | **0.89** | 0.27 | -0.03 |  | **0.66** |  | 0.32 | **0.75** | **0.65** | **0.63** | **0.52** |  | **0.74** |
| 2-NFLT |  | 0.22 |  | **0.70** | **1.00** | **0.74** | **0.58** | **0.62** | 0.34 | **0.82** | 0.23 | -0.02 |  | 0.25 |  | 0.08 | **0.46** | **0.42** | **0.33** | **0.56** |  | **0.38** |
| 3-NFLT |  | 0.16 |  | **0.58** | **0.74** | **1.00** | **0.52** | **0.80** | **0.48** | **0.75** | 0.30 | 0.15 |  | **0.45** |  | 0.29 | **0.67** | **0.71** | **0.62** | **0.83** |  | **0.60** |
| 1-NPYR |  | **0.49** |  | **0.86** | **0.58** | **0.52** | **1.00** | **0.68** | **0.46** | **0.87** | 0.21 | 0.11 |  | **0.63** |  | 0.28 | **0.78** | **0.70** | **0.72** | **0.56** |  | **0.75** |
| 2-NPYR |  | 0.39 |  | **0.73** | **0.62** | **0.80** | **0.68** | **1.00** | **0.84** | **0.86** | **0.44** | 0.43 |  | **0.58** |  | 0.24 | **0.75** | **0.80** | **0.79** | **0.84** |  | **0.70** |
| 7-NBAA |  | 0.28 |  | **0.46** | 0.34 | **0.48** | **0.46** | **0.84** | **1.00** | **0.64** | **0.50** | **0.57** |  | **0.48** |  | 0.27 | **0.50** | **0.60** | **0.74** | **0.66** |  | **0.49** |
| **Σ9NPAHs** |  | **0.53** |  | **0.89** | **0.82** | **0.75** | **0.87** | **0.86** | **0.64** | **1.00** | 0.35 | 0.19 |  | **0.57** |  | 0.19 | **0.72** | **0.69** | **0.69** | **0.71** |  | **0.70** |
| 1,4-O2NAP |  | **0.62** |  | 0.27 | 0.23 | 0.30 | 0.21 | **0.44** | **0.50** | 0.35 | **1.00** | **0.72** |  | 0.34 |  | 0.04 | 0.35 | 0.38 | **0.39** | 0.36 |  | 0.24 |
| 1(CHO)NAP |  | **0.59** |  | -0.03 | -0.02 | 0.15 | 0.11 | 0.43 | **0.57** | 0.19 | **0.72** | **1.00** |  | 0.38 |  | -0.23 | 0.49 | **0.56** | **0.54** | **0.62** |  | **0.65** |
| 9-OFLN |  |  |  |  |  |  |  |  |  |  |  |  |  |  |  |  |  |  |  |  |  |  |
| 9,10-O2ANT |  | **0.68** |  | **0.66** | 0.25 | **0.45** | **0.63** | **0.58** | **0.48** | **0.57** | 0.34 | 0.38 |  | **1.00** |  | **0.67** | **0.66** | **0.59** | **0.62** | **0.53** |  | **0.77** |
| 1,4-O2ANT |  |  |  |  |  |  |  |  |  |  |  |  |  |  |  |  |  |  |  |  |  |  |
| 9,10-O2PHE |  | 0.28 |  | **0.32** | 0.08 | 0.29 | 0.28 | 0.24 | 0.27 | 0.19 | 0.04 | -0.23 |  | **0.67** |  | **1.00** | 0.22 | 0.19 | 0.23 | 0.19 |  | **0.44** |
| BaOFLN |  | **0.48** |  | **0.75** | **0.46** | **0.67** | **0.78** | **0.75** | **0.50** | **0.72** | 0.35 | 0.49 |  | **0.66** |  | 0.22 | **1.00** | **0.94** | **0.88** | **0.80** |  | **0.91** |
| BbOFLN |  | **0.46** |  | **0.65** | **0.42** | **0.71** | **0.70** | **0.80** | **0.60** | **0.69** | 0.38 | **0.56** |  | **0.59** |  | 0.19 | **0.94** | **1.00** | **0.93** | **0.88** |  | **0.86** |
| BAN |  | **0.49** |  | **0.63** | 0.33 | **0.62** | **0.72** | **0.79** | **0.74** | **0.69** | **0.39** | **0.54** |  | **0.62** |  | 0.23 | **0.88** | **0.93** | **1.00** | **0.84** |  | **0.87** |
| 7,12-O2BAA |  | 0.33 |  | **0.52** | **0.56** | **0.83** | **0.56** | **0.84** | **0.66** | **0.71** | 0.36 | **0.62** |  | **0.53** |  | 0.19 | **0.80** | **0.88** | **0.84** | **1.00** |  | **0.75** |
| 5,12-O2NAC |  |  |  |  |  |  |  |  |  |  |  |  |  |  |  |  |  |  |  |  |  |  |
| **Σ11OPAHs** |  | **0.58** |  | **0.74** | **0.38** | **0.60** | **0.75** | **0.70** | **0.49** | **0.70** | 0.24 | **0.65** |  | **0.77** |  | **0.44** | **0.91** | **0.86** | **0.87** | **0.75** |  | **1.00** |

Table S31: Pearson correlations between individual NOPAHs at the Ljubljana traffic site in summer. Numbers in bold indicate cases significant at the 95% confidence level, while those underlined are significant at the 99% confidence level

|  | 1-NNAP | 2-NNAP | 9-NANT | 3-NPHE | 2-NFLT | 3-NFLT | 1-NPYR | 2-NPYR | 7-NBAA | **Σ9NPAHs** | 1,4-O_2_NAP | 1(CHO)NAP | 9-OFLN | 9,10-O_2_ANT | 1,4-O_2_ANT | 9,10-O_2_PHE | BaOFLN | BbOFLN | BAN | 7,12-O_2_BAA | 5,12-O_2_NAC | **Σ11OPAHs** |
| --- | --- | --- | --- | --- | --- | --- | --- | --- | --- | --- | --- | --- | --- | --- | --- | --- | --- | --- | --- | --- | --- | --- |
| 1-NNAP |  |  |  |  |  |  |  |  |  |  |  |  |  |  |  |  |  |  |  |  |  |  |
| 2-NNAP |  |  |  |  |  |  |  |  |  |  |  |  |  |  |  |  |  |  |  |  |  |  |
| 9-NANT |  |  | **1.00** | 0.90 | **0.89** | 0.70 | 0.37 | **0.89** | 1.00 | **0.94** |  |  |  |  |  |  |  |  |  |  |  |  |
| 3-NPHE |  |  | 0.90 | **1.00** | **0.73** | **0.82** | **0.70** | **0.68** | 0.25 | **0.75** |  | 0.50 |  |  |  |  |  |  | -0.50 |  | 0.46 | 0.75 |
| 2-NFLT |  |  | **0.89** | **0.73** | **1.00** | **0.83** | **0.67** | **0.68** | 0.47 | **0.83** |  | 0.80 |  |  |  |  |  |  | 0.50 |  | 0.19 | 0.33 |
| 3-NFLT |  |  | 0.70 | **0.82** | **0.83** | **1.00** | **0.65** | **0.72** | 0.40 | **0.78** |  | 0.80 |  |  |  |  |  |  | 0.50 |  | 0.07 | 0.50 |
| 1-NPYR |  |  | 0.37 | **0.70** | **0.67** | **0.65** | **1.00** | **0.53** | 0.37 | **0.58** |  | 0.60 |  |  |  |  |  |  | -0.50 |  | 0.31 | 0.25 |
| 2-NPYR |  |  | **0.89** | **0.68** | **0.68** | **0.72** | **0.53** | **1.00** | **0.59** | **0.81** |  | 0.40 |  |  |  |  |  |  | 0.50 |  | 0.43 | 0.42 |
| 7-NBAA |  |  | 1.00 | 0.25 | 0.47 | 0.40 | 0.37 | 0.59 | **1.00** | **0.81** |  |  |  |  |  |  |  |  |  |  | **0.82** | 0.57 |
| **Σ9NPAHs** |  |  | **0.94** | **0.75** | **0.83** | **0.78** | **0.58** | **0.81** | **0.81** | **1.00** |  | 0.40 |  |  |  |  |  |  | 1.00 |  | 0.52 | 0.20 |
| 1,4-O2NAP |  |  |  |  |  |  |  |  |  |  |  |  |  |  |  |  |  |  |  |  |  |  |
| 1(CHO)NAP |  |  |  | 0.50 | 0.80 | 0.80 | 0.60 | 0.40 |  | 0.40 |  | 1.00 |  |  |  |  |  |  |  |  | -1.00 | 0.80 |
| 9-OFLN |  |  |  |  |  |  |  |  |  |  |  |  |  |  |  |  |  |  |  |  |  |  |
| 9,10-O2ANT |  |  |  |  |  |  |  |  |  |  |  |  |  |  |  |  |  |  |  |  |  |  |
| 1,4-O2ANT |  |  |  |  |  |  |  |  |  |  |  |  |  |  |  |  |  |  |  |  |  |  |
| 9,10-O2PHE |  |  |  |  |  |  |  |  |  |  |  |  |  |  |  |  |  |  |  |  |  |  |
| BaOFLN |  |  |  |  |  |  |  |  |  |  |  |  |  |  |  |  |  |  |  |  |  |  |
| BbOFLN |  |  |  |  |  |  |  |  |  |  |  |  |  |  |  |  |  |  |  |  |  |  |
| BAN |  |  |  | -0.50 | 0.50 | 0.50 | -0.50 | 0.50 |  | 1.00 |  |  |  |  |  |  |  |  | 1.00 |  | 0.50 | 0.50 |
| 7,12-O2BAA |  |  |  |  |  |  |  |  |  |  |  |  |  |  |  |  |  |  |  |  |  |  |
| 5,12-O2NAC |  |  |  | 0.46 | 0.19 | 0.07 | 0.31 | 0.43 | **0.82** | 0.52 |  | -1.00 |  |  |  |  |  |  | 0.50 |  | **1.00** | 0.48 |
| **Σ11OPAHs** |  |  |  | 0.75 | 0.33 | 0.50 | 0.25 | 0.42 | 0.57 | 0.20 |  | 0.80 |  |  |  |  |  |  | 0.50 |  | 0.48 | **1.00** |

Table S32: Statistics of the ratios of individual NOPAHs and parent PAHs in winter

|  | | | 1NNAP/NAP | 2NNAP/NAP | 9NANT/ANT | 3NPHE/PHE | 2NFLT/FLT | 1NPYR/PYR | 2NPYR/PYR | 7NBAA/BAA | 1,4O2NAP/NAP | 9,10-O2ANT/ANT | 1,4-O2ANT/ANT | 9,10-O2PHE/PHE |
| --- | --- | --- | --- | --- | --- | --- | --- | --- | --- | --- | --- | --- | --- | --- |
| R | | Min | 0.04 |  | 0.70 | 3.56E-03 | 1.54E-02 | 5.72E-04 | 1.42E-03 | 6.14E-03 | 0.87 | 10.32 | 0.08 | 0.24 |
|  |  | Max | 0.10 |  | 3.88 | 7.67E-03 | 3.20E-02 | 2.20E-03 | 4.29E-03 | 1.41E-02 | 2.40 | 36.88 | 0.14 | 1.58 |
|  |  | Average | 0.06 |  | 1.78 | 4.73E-03 | 2.24E-02 | 1.13E-03 | 1.98E-03 | 9.27E-03 | 1.63 | 19.97 | 0.11 | 0.90 |
|  |  | SD | 0.03 |  | 1.20 | 1.97E-03 | 7.00E-03 | 5.24E-04 | 9.61E-04 | 2.83E-03 | 0.71 | 11.07 | 0.02 | 0.47 |
| Brno | UB | Min |  |  | 0.30 |  | 1.88E-02 | 1.63E-03 | 3.49E-03 | 1.17E-02 | 0.73 | 6.02 |  |  |
|  |  | Max |  |  | 2.28 |  | 1.17E-01 | 1.83E-02 | 3.33E-02 | 3.57E-02 | 6.79 | 44.29 |  |  |
|  |  | Average |  |  | 0.81 |  | 4.82E-02 | 7.03E-03 | 1.26E-02 | 2.08E-02 | 3.06 | 18.44 |  |  |
|  |  | SD |  |  | 0.60 |  | 2.41E-02 | 3.96E-03 | 6.91E-03 | 6.84E-03 | 1.81 | 13.35 |  |  |
|  | T | Min | 0.02 | 0.00 | 0.33 | 8.44E-03 | 7.75E-03 | 3.82E-03 | 8.34E-04 | 4.30E-03 | 0.45 | 5.55 | 0.03 | 0.31 |
|  |  | Max | 0.26 | 0.12 | 31.09 | 8.10E-02 | 1.37E-01 | 4.25E-02 | 4.55E-02 | 1.44E-01 | 29.75 | 62.43 | 0.12 | 1.52 |
|  |  | Average | 0.10 | 0.04 | 5.80 | 2.46E-02 | 4.63E-02 | 1.35E-02 | 1.29E-02 | 3.50E-02 | 8.29 | 20.25 | 0.08 | 0.83 |
|  |  | SD | 0.08 | 0.03 | 7.41 | 1.99E-02 | 3.28E-02 | 9.01E-03 | 1.19E-02 | 3.27E-02 | 7.92 | 17.46 | 0.03 | 0.41 |
| Ljubljana | UB | Min |  |  | 0.02 | 1.22E-03 | 5.78E-04 | 1.59E-04 | 2.47E-04 | 7.57E-04 |  | 0.32 |  |  |
|  |  | Max |  |  | 0.33 | 2.37E-03 | 8.01E-03 | 7.84E-04 | 2.58E-03 | 7.89E-03 |  | 1.73 |  |  |
|  |  | Average |  |  | 0.11 | 1.83E-03 | 2.47E-03 | 3.88E-04 | 1.50E-03 | 3.82E-03 |  | 0.79 |  |  |
|  |  | SD |  |  | 0.09 | 4.39E-04 | 1.71E-03 | 1.74E-04 | 6.23E-04 | 2.10E-03 |  | 0.45 |  |  |
|  | T | Min |  |  | 0.02 | 1.47E-03 | 1.58E-04 | 5.80E-05 | 1.01E-04 | 8.41E-04 | 0.02 | 0.09 |  |  |
|  |  | Max |  |  | 0.20 | 1.58E-03 | 3.81E-03 | 1.11E-03 | 2.56E-03 | 5.55E-03 | 0.02 | 1.37 |  |  |
|  |  | Average |  |  | 0.09 | 1.53E-03 | 1.91E-03 | 4.85E-04 | 1.13E-03 | 2.51E-03 |  | 0.69 |  |  |
|  |  | SD |  |  | 0.06 | 7.66E-05 | 1.21E-03 | 2.63E-04 | 6.47E-04 | 1.46E-03 |  | 0.34 |  |  |

Table S33: Statistics of the ratios of individual NOPAHs and parent PAHs in summer

|  | | | 1NNAP/NAP | 2NNAP/NAP | 9NANT/ANT | 3NPHE/PHE | 2NFLT/FLT | 1NPYR/PYR | 2NPYR/PYR | 7NBAA/BAA | 1,4O2NAP/NAP | 9,10-O2ANT/ANT | 1,4-O2ANT/ANT | 9,10-O2PHE/PHE |
| --- | --- | --- | --- | --- | --- | --- | --- | --- | --- | --- | --- | --- | --- | --- |
| R | | Min |  |  |  |  |  |  |  |  |  |  |  |  |
|  |  | Max |  |  |  |  |  |  |  |  |  |  |  |  |
|  |  | Average |  |  |  |  |  |  |  |  |  |  |  |  |
|  |  | SD |  |  |  |  |  |  |  |  |  |  |  |  |
| Brno | UB | Min |  |  |  |  |  |  |  |  |  |  |  |  |
|  |  | Max |  |  |  |  |  |  |  |  |  |  |  |  |
|  |  | Average |  |  |  |  |  |  |  |  |  |  |  |  |
|  |  | SD |  |  |  |  |  |  |  |  |  |  |  |  |
|  | T | Min |  |  |  | 6.29E-02 | 2.90E-03 | 1.28E-02 | 1.11E-03 | 9.60E-03 |  | 0.06 |  | 1.26 |
|  |  | Max |  |  |  | 2.34E-01 | 7.55E-02 | 6.31E-02 | 9.89E-03 | 2.70E-01 |  | 0.17 |  | 3.62 |
|  |  | Average |  |  |  | 1.56E-01 | 1.76E-02 | 3.51E-02 | 4.50E-03 | 9.79E-02 |  | 0.11 |  | 2.25 |
|  |  | SD |  |  |  | 4.67E-02 | 1.47E-02 | 1.21E-02 | 2.29E-03 | 6.54E-02 |  | 0.03 |  | 0.73 |
| Ljubljana | UB | Min |  |  |  |  |  | 9.88E-04 |  |  | 0.84 |  |  | 1.49 |
|  |  | Max |  |  |  |  |  | 3.79E-02 |  |  | 1.11 |  |  | 1.54 |
|  |  | Average |  |  |  |  |  | 2.13E-02 |  |  | 1.01 |  |  | 1.52 |
|  |  | SD |  |  |  |  |  | 1.31E-02 |  |  | 0.14 |  |  | 0.04 |
|  | T | Min |  |  | 0.02 | 5.88E-02 | 1.17E-02 | 3.55E-03 | 5.29E-03 | 1.42E-01 |  |  |  |  |
|  |  | Max |  |  | 0.12 | 5.88E-02 | 1.28E-01 | 1.02E-01 | 3.27E-02 | 4.94E-01 |  |  |  |  |
|  |  | Average |  |  | 0.08 | 5.88E-02 | 8.42E-02 | 4.31E-02 | 1.86E-02 | 3.36E-01 |  |  |  |  |
|  |  | SD |  |  | 0.04 |  | 3.36E-02 | 2.16E-02 | 7.45E-03 | 1.46E-01 |  |  |  |  |

Table S34: Statistics of the cancer risks from particulate NOPAHs

|  | | | Min | Max | Average | Median | SD | % of samples exceeding the acceptable lifetime carcinogenic risk |
| --- | --- | --- | --- | --- | --- | --- | --- | --- |
| Winter | Rural | | 6.19E-08 | 1.72E-06 | 7.69E-07 | 7.67E-07 | 5.08E-07 | 38 |
|  | Brno | Urban background | 2.01E-08 | 2E-05 | 2.28E-06 | 2.76E-07 | 4.75E-06 | 37 |
|  |  | Traffic | 1.65E-07 | 1.29E-05 | 2.94E-06 | 8.45E-07 | 3.82E-06 | 47 |
|  | Ljubljana | Urban background | 5.58E-08 | 2.47E-06 | 7.92E-07 | 3.15E-07 | 8.01E-07 | 33 |
|  |  | Traffic | 2.68E-08 | 2.26E-06 | 6.64E-07 | 3.41E-07 | 6.76E-07 | 27 |
| Summer | Rural | | 3.37E-10 | 2.39E-09 | 8.21E-10 | 5.59E-10 | 6.64E-10 | 0 |
|  | Brno | Urban background | 0 | 5.48E-09 | 1.83E-10 | 0 | 1E-09 | 0 |
|  |  | Traffic | 9.28E-09 | 7.53E-08 | 3.51E-08 | 3.57E-08 | 1.74E-08 | 0 |
|  | Ljubljana | Urban background | 0 | 1.43E-08 | 2.89E-09 | 0 | 4.64E-09 | 0 |
|  |  | Traffic | 4.81E-11 | 3.93E-07 | 7.78E-08 | 6.67E-08 | 7.26E-08 | 0 |

Table S35: Statistics of the cancer risks from particulate NOPAHs and PAHs

|  | | | Min | Max | Average | Median | SD | % of samples exceeding the acceptable lifetime carcinogenic risk |
| --- | --- | --- | --- | --- | --- | --- | --- | --- |
| Winter | Rural | | 3.09E-05 | 6.41E-04 | 3.37E-04 | 3.76E-04 | 2.24E-04 | 100 |
|  | Brno | Urban background | 3.46E-05 | 2.83E-03 | 5.29E-04 | 2.88E-04 | 6.18E-04 | 100 |
|  |  | Traffic | 5.91E-05 | 1.94E-03 | 5.71E-04 | 4.76E-04 | 4.74E-04 | 100 |
|  | Ljubljana | Urban background | 4.12E-04 | 1.58E-02 | 5.03E-03 | 2.71E-03 | 4.66E-03 | 100 |
|  |  | Traffic | 3.63E-04 | 1.95E-02 | 4.69E-03 | 2.61E-03 | 5.00E-03 | 100 |
| Summer | Rural | | 2.39E-06 | 2.01E-05 | 6.34E-06 | 4.31E-06 | 5.8E-06 | 100 |
|  | Brno | Urban background | 0 | 1.02E-04 | 3.63E-05 | 3.34E-05 | 2.87E-05 | 77 |
|  |  | Traffic | 2.84E-05 | 1.12E-04 | 5.93E-05 | 5.83E-05 | 2.04E-05 | 100 |
|  | Ljubljana | Urban background | 1.51E-07 | 8.65E-04 | 1.22E-04 | 7.92E-05 | 1.96E-04 | 77 |
|  |  | Traffic | 3.1E-08 | 1.04E-03 | 1.32E-04 | 7.82E-05 | 1.96E-04 | 88 |

Table S36: Statistics of NPAH levels in the gaseous phase. Compounds that were never found in the gaseous phase are not included

|  |  |  | 1-NNAP | 2-NNAP | 5-NACE | 9-NANT | 3-NPHE | 2-NFLT | 3-NFLT | 1-NPYR | 2-NPYR | 7-NBAA | **Σ9NPAHs** |
| --- | --- | --- | --- | --- | --- | --- | --- | --- | --- | --- | --- | --- | --- |
| **Winter** | **R** | **DF** | 100 | 100 | 75 | 100 | 75 | 38 | 0 | 50 | 0 | 0 | 100 |
|  |  | **Min** | 3.85 | 3.07 | 0.00 | 2.59 | 0.00 | 0.00 |  | 0.00 |  |  | 16.82 |
|  |  | **Max** | 51.51 | 30.12 | 0.67 | 21.15 | 4.39 | 1.39 |  | 0.26 |  |  | 101.28 |
|  |  | **Average** | 18.30 | 12.70 | 0.38 | 12.28 | 1.89 | 0.29 |  | 0.07 |  |  | 45.52 |
|  |  | **SD** | 14.71 | 8.16 | 0.25 | 6.31 | 1.50 | 0.49 |  | 0.09 |  |  | 25.91 |
|  | **UB** | **DF** | 100 | 100 | 0 | 100 | 0 | 0 | 0 | 14 | 0 | 0 | 100 |
|  |  | **Min** | 49.67 | 20.87 |  | 15.85 |  |  |  | 0.00 |  |  | 102.04 |
|  |  | **Max** | 88.14 | 46.44 |  | 31.49 |  |  |  | 0.78 |  |  | 151.92 |
|  |  | **Average** | 67.76 | 33.53 |  | 23.49 |  |  |  | 0.11 |  |  | 124.89 |
|  |  | **SD** | 12.57 | 8.22 |  | 5.44 |  |  |  | 0.29 |  |  | 18.93 |
|  | **T** | **DF** | 100 | 100 | 100 | 100 | 100 | 71 | 0 | 29 | 0 | 0 | 100 |
|  |  | **Min** | 50.56 | 12.69 | 0.92 | 26.66 | 2.48 | 0.00 |  | 0.00 |  |  | 93.56 |
|  |  | **Max** | 246.87 | 75.36 | 5.35 | 58.28 | 7.77 | 0.73 |  | 0.16 |  |  | 364.42 |
|  |  | **Average** | 152.61 | 44.73 | 3.47 | 43.77 | 3.92 | 0.44 |  | 0.04 |  |  | 245.51 |
|  |  | **SD** | 66.31 | 20.45 | 1.47 | 11.03 | 1.78 | 0.31 |  | 0.07 |  |  | 89.01 |
| **Summer** | **R** | **DF** | 100 | 100 | 100 | 0 | 100 | 100 | 0 | 75 | 25 | 0 | 100 |
|  |  | **Min** | 3.54 | 1.05 | 2.06 |  | 0.36 | 0.32 |  | 0.00 | 0.00 |  | 7.29 |
|  |  | **Max** | 10.57 | 3.13 | 9.17 |  | 1.25 | 0.93 |  | 0.09 | 0.06 |  | 15.03 |
|  |  | **Average** | 7.17 | 2.39 | 4.00 |  | 0.65 | 0.58 |  | 0.04 | 0.01 |  | 10.85 |
|  |  | **SD** | 2.69 | 0.73 | 2.32 |  | 0.27 | 0.24 |  | 0.03 | 0.02 |  | 3.34 |
|  | **UB** | **DF** | 100 | 100 | 100 | 0 | 0 | 100 | 0 | 100 | 0 | 0 | 100 |
|  |  | **Min** | 47.04 | 6.18 | 6.79 |  | 0.00 | 1.64 | 0.00 | 0.51 |  |  | 59.83 |
|  |  | **Max** | 78.02 | 16.20 | 42.21 |  | 0.00 | 3.72 | 0.00 | 1.58 |  |  | 92.87 |
|  |  | **Average** | 61.68 | 11.45 | 22.88 |  | 0.00 | 2.57 | 0.00 | 1.03 |  |  | 76.73 |
|  |  | **SD** | 11.84 | 3.00 | 12.53 |  | 0.00 | 0.80 | 0.00 | 0.37 |  |  | 13.97 |
|  | **T** | **DF** | 100 | 100 | 100 | 0 | 100 | 100 | 0 | 63 | 0 | 0 | 100 |
|  |  | **Min** | 27.18 | 5.94 | 8.26 |  | 1.40 | 0.37 |  | 0.00 |  |  | 39.13 |
|  |  | **Max** | 41.14 | 11.51 | 46.53 |  | 8.03 | 0.79 |  | 0.35 |  |  | 57.44 |
|  |  | **Average** | 33.90 | 8.24 | 26.06 |  | 4.91 | 0.55 |  | 0.17 |  |  | 47.76 |
|  |  | **SD** | 5.62 | 1.55 | 13.73 |  | 2.54 | 0.15 |  | 0.15 |  |  | 7.16 |

Table S37: Statistics of OPAH levels in the gaseous phase

|  |  |  | 1,4-O_2_NAP | 1(CHO)NAP | 9-OFLN | 9,10-O_2_ANT | 1,4-O_2_ANT | 9,10-O_2_PHE | BaOFLN | BbOFLN | BAN | 7,12-O_2_BAA | 5,12-O_2_NAC | **Σ11OPAHs** |
| --- | --- | --- | --- | --- | --- | --- | --- | --- | --- | --- | --- | --- | --- | --- |
| **Winter** | **R** | **DF** | 100 | 100 | 100 | 100 | 100 | 75 | 88 | 25 | 13 | 38 | 13 | 100 |
|  |  | **Min** | 153.57 | 141.66 | 186.76 | 63.50 | 0.92 | 0.00 | 0.00 | 0.00 | 0.00 | 0.00 | 0.00 | 1163.10 |
|  |  | **Max** | 3531.06 | 2065.04 | 791.46 | 856.68 | 3.13 | 1231.88 | 20.40 | 5.40 | 9.94 | 4.39 | 0.22 | 7259.47 |
|  |  | **Average** | 1617.03 | 826.12 | 498.39 | 348.52 | 1.85 | 590.84 | 10.60 | 1.08 | 1.24 | 0.86 | 0.03 | 3896.55 |
|  |  | **SD** | 1242.57 | 637.06 | 170.03 | 313.05 | 0.68 | 441.95 | 6.65 | 2.08 | 3.52 | 1.54 | 0.08 | 2015.86 |
|  | **UB** | **DF** | 100 | 100 | 100 | 100 | 43 | 86 | 43 | 0 | 0 | 0 | 0 | 100 |
|  |  | **Min** | 1467.97 | 1057.89 | 1645.40 | 1538.52 | 0.00 | 0.00 | 0.00 |  |  |  |  | 5849.54 |
|  |  | **Max** | 6933.71 | 2264.83 | 2795.92 | 2377.50 | 36.69 | 3802.63 | 63.38 |  |  |  |  | 15818.14 |
|  |  | **Average** | 4022.81 | 1531.09 | 2328.68 | 1868.05 | 9.56 | 2427.45 | 26.11 |  |  |  |  | 12213.76 |
|  |  | **SD** | 2203.35 | 410.16 | 452.25 | 307.44 | 13.92 | 1341.34 | 32.62 |  |  |  |  | 3760.56 |
|  | **T** | **DF** | 100 | 100 | 100 | 100 | 71 | 71 | 100 | 14 | 0 | 43 | 57 | 100 |
|  |  | **Min** | 1010.20 | 557.27 | 660.84 | 813.00 | 0.00 | 0.00 | 3.87 | 0.00 |  | 0.00 | 0.00 | 4687.61 |
|  |  | **Max** | 4329.96 | 3181.28 | 999.45 | 1768.49 | 7.39 | 3915.87 | 27.26 | 3.60 |  | 1.40 | 0.46 | 12145.43 |
|  |  | **Average** | 2458.73 | 2011.85 | 874.18 | 1272.39 | 4.00 | 1383.90 | 14.94 | 0.51 |  | 0.57 | 0.17 | 8021.25 |
|  |  | **SD** | 1332.93 | 998.29 | 112.92 | 334.85 | 3.26 | 1677.76 | 8.88 | 1.36 |  | 0.71 | 0.18 | 2553.19 |
| **Summer** | **R** | **DF** | 100 | 100 | 100 | 100 | 0 | 0 | 100 | 75 | 38 | 100 | 88 | 100 |
|  |  | **Min** | 56.38 | 12.45 | 28.48 | 31.03 |  |  | 4.17 | 0.00 | 0.00 | 1.10 | 0.00 | 168.55 |
|  |  | **Max** | 121.14 | 48.92 | 52.77 | 77.41 |  |  | 8.61 | 6.35 | 4.03 | 3.05 | 0.12 | 275.45 |
|  |  | **Average** | 84.28 | 24.86 | 36.69 | 59.44 |  |  | 6.73 | 3.53 | 1.35 | 1.79 | 0.08 | 218.75 |
|  |  | **SD** | 20.42 | 11.45 | 7.44 | 16.83 |  |  | 1.79 | 2.41 | 1.88 | 0.64 | 0.04 | 39.17 |
|  | **UB** | **DF** | 100 | 100 | 100 | 88 | 0 | 0 | 63 | 50 | 25 | 75 | 88 | 100 |
|  |  | **Min** | 295.34 | 108.17 | 334.06 | 0.00 |  |  | 0.00 | 0.00 | 0.00 | 0.00 | 0.00 | 1116.38 |
|  |  | **Max** | 2143.12 | 271.61 | 401.03 | 482.31 |  |  | 47.27 | 45.11 | 53.03 | 22.01 | 0.91 | 3233.04 |
|  |  | **Average** | 756.62 | 201.10 | 375.47 | 334.18 |  |  | 27.19 | 22.01 | 12.51 | 11.82 | 0.65 | 1741.56 |
|  |  | **SD** | 618.53 | 52.96 | 25.01 | 145.35 |  |  | 22.68 | 23.54 | 23.22 | 8.30 | 0.29 | 733.66 |
|  | **T** | **DF** | 88 | 100 | 100 | 100 | 0 | 0 | 75 | 50 | 0 | 100 | 88 | 100 |
|  |  | **Min** | 0.00 | 52.44 | 43.45 | 51.73 |  |  | 0.00 | 0.00 |  | 1.06 | 0.00 | 191.22 |
|  |  | **Max** | 471.53 | 103.63 | 70.09 | 90.63 |  |  | 14.87 | 3.31 |  | 2.27 | 0.22 | 735.59 |
|  |  | **Average** | 123.26 | 84.02 | 56.70 | 70.11 |  |  | 6.55 | 1.47 |  | 1.82 | 0.13 | 344.06 |
|  |  | **SD** | 148.96 | 14.95 | 9.22 | 12.42 |  |  | 5.42 | 1.58 |  | 0.42 | 0.06 | 172.11 |

Table S38: Statistics of NPAHs particulate mass fractions

|  | | | 1-NNAP | 2-NNAP | 5-NACE | 2-NFLN | 9-NANT | 3-NPHE | 2-NFLT | 3-NFLT | 1-NPYR | 2-NPYR | 7-NBAA | 6-NCHR | 1,3-N_2_PYR | Σ9NPAHs |
| --- | --- | --- | --- | --- | --- | --- | --- | --- | --- | --- | --- | --- | --- | --- | --- | --- |
| Winter | R | N | 8 | 8 | 6 | 0 | 8 | 6 | 8 | 7 | 8 | 8 | 7 | 2 | 0 | 8 |
|  |  | Min | 0.00 | 0.00 | 0.00 |  | 0.09 | 0.00 | 0.89 | 1.00 | 0.00 | 1.00 | 1.00 | 1.00 |  | 0.14 |
|  |  | Max | 0.01 | 0.00 | 0.00 |  | 0.93 | 0.64 | 1.00 | 1.00 | 1.00 | 1.00 | 1.00 | 1.00 |  | 0.81 |
|  |  | Average | 0.00 | 0.00 | 0.00 |  | 0.59 | 0.27 | 0.98 | 1.00 | 0.83 | 1.00 | 1.00 | 1.00 |  | 0.52 |
|  |  | SD | 0.00 | 0.00 | 0.00 |  | 0.36 | 0.26 | 0.04 | 0.00 | 0.34 | 0.00 | 0.00 | 0.00 |  | 0.24 |
|  | UB | N | 7 | 7 | 0 | 0 | 7 | 0 | 7 | 3 | 7 | 7 | 3 | 0 | 0 | 7 |
|  |  | Min | 0.00 | 0.00 |  |  | 0.22 |  | 1.00 | 1.00 | 0.88 | 1.00 | 1.00 |  |  | 0.32 |
|  |  | Max | 0.01 | 0.00 |  |  | 0.96 |  | 1.00 | 1.00 | 1.00 | 1.00 | 1.00 |  |  | 0.91 |
|  |  | Average | 0.00 | 0.00 |  |  | 0.55 |  | 1.00 | 1.00 | 0.98 | 1.00 | 1.00 |  |  | 0.55 |
|  |  | SD | 0.00 | 0.00 |  |  | 0.33 |  | 0.00 | 0.00 | 0.05 | 0.00 | 0.00 |  |  | 0.26 |
|  | T | N | 7 | 7 | 7 | 1 | 7 | 7 | 7 | 7 | 7 | 7 | 7 | 0 | 3 | 7 |
|  |  | Min | 0.01 | 0.01 | 0.00 |  | 0.68 | 0.51 | 0.98 | 1.00 | 1.00 | 1.00 | 1.00 |  | 1.00 | 0.48 |
|  |  | Max | 0.02 | 0.04 | 0.00 |  | 0.98 | 0.94 | 1.00 | 1.00 | 1.00 | 1.00 | 1.00 |  | 1.00 | 0.90 |
|  |  | Average | 0.01 | 0.02 | 0.00 | 1.00 | 0.87 | 0.74 | 1.00 | 1.00 | 1.00 | 1.00 | 1.00 |  | 1.00 | 0.74 |
|  |  | SD | 0.01 | 0.01 | 0.00 |  | 0.12 | 0.19 | 0.01 | 0.00 | 0.00 | 0.00 | 0.00 |  | 0.00 | 0.17 |
| Summer | R | N | 8 | 8 | 8 | 0 | 0 | 8 | 8 | 0 | 6 | 2 | 0 | 0 | 0 | 8 |
|  |  | Min | 0.00 | 0.00 | 0.00 |  |  | 0.00 | 0.00 |  | 0.00 | 0.00 |  |  |  | 0.00 |
|  |  | Max | 0.00 | 0.00 | 0.00 |  |  | 0.00 | 0.00 |  | 0.00 | 0.00 |  |  |  | 0.00 |
|  |  | Average | 0.00 | 0.00 | 0.00 |  |  | 0.00 | 0.00 |  | 0.00 | 0.00 |  |  |  | 0.00 |
|  |  | SD | 0.00 | 0.00 | 0.00 |  |  | 0.00 | 0.00 |  | 0.00 | 0.00 |  |  |  | 0.00 |
|  | UB | N | 8 | 8 | 8 | 0 | 0 | 0 | 8 | 0 | 8 | 0 | 0 | 0 | 0 | 8 |
|  |  | Min | 0.00 | 0.00 | 0.00 |  |  |  | 0.00 |  | 0.00 |  |  |  |  | 0.00 |
|  |  | Max | 0.00 | 0.00 | 0.00 |  |  |  | 0.00 |  | 0.00 |  |  |  |  | 0.00 |
|  |  | Average | 0.00 | 0.00 | 0.00 |  |  |  | 0.00 |  | 0.00 |  |  |  |  | 0.00 |
|  |  | SD | 0.00 | 0.00 | 0.00 |  |  |  | 0.00 |  | 0.00 |  |  |  |  | 0.00 |
|  | T | N | 8 | 8 | 8 | 0 | 0 | 8 | 8 | 8 | 8 | 8 | 8 | 0 | 0 | 8 |
|  |  | Min | 0.00 | 0.00 | 0.00 |  |  | 0.06 | 0.41 | 1.00 | 0.79 | 1.00 | 1.00 |  |  | 0.05 |
|  |  | Max | 0.00 | 0.03 | 0.00 |  |  | 0.32 | 0.86 | 1.00 | 1.00 | 1.00 | 1.00 |  |  | 0.18 |
|  |  | Average | 0.00 | 0.01 | 0.00 |  |  | 0.15 | 0.65 | 1.00 | 0.90 | 1.00 | 1.00 |  |  | 0.09 |
|  |  | SD | 0.00 | 0.01 | 0.00 |  |  | 0.10 | 0.13 | 0.00 | 0.09 | 0.00 | 0.00 |  |  | 0.04 |

Table S39: Statistics of OPAHs particulate mass fractions

|  |  |  | 1,4-O_2_NAP | 1(CHO)NAP | 9-OFLN | 9,10-O_2_ANT | 1,4-O_2_ANT | 9,10-O_2_PHE | BaOFLN | BbOFLN | BAN | 7,12-O_2_BAA | 5,12-O_2_NAC | **Σ11OPAHs** |
| --- | --- | --- | --- | --- | --- | --- | --- | --- | --- | --- | --- | --- | --- | --- |
| Winter | R | N | 8 | 8 | 8 | 8 | 8 | 8 | 8 | 8 | 8 | 8 | 8 | 8 |
|  |  | Min | 0.00 | 0.00 | 0.00 | 0.12 | 0.00 | 0.00 | 0.32 | 0.87 | 0.89 | 0.81 | 0.99 | 0.06 |
|  |  | Max | 0.01 | 0.01 | 0.18 | 0.89 | 0.84 | 1.00 | 1.00 | 1.00 | 1.00 | 1.00 | 1.00 | 0.30 |
|  |  | Average | 0.00 | 0.00 | 0.03 | 0.49 | 0.42 | 0.39 | 0.75 | 0.98 | 0.99 | 0.97 | 1.00 | 0.17 |
|  |  | SD | 0.00 | 0.00 | 0.07 | 0.27 | 0.31 | 0.40 | 0.24 | 0.05 | 0.04 | 0.06 | 0.00 | 0.07 |
|  | UB | N | 7 | 7 | 7 | 7 | 3 | 6 | 7 | 7 | 7 | 7 | 7 | 7 |
|  |  | Min | 0.00 | 0.00 | 0.00 | 0.00 | 0.00 | 0.00 | 0.51 | 1.00 | 1.00 | 1.00 | 1.00 | 0.06 |
|  |  | Max | 0.07 | 0.09 | 0.27 | 0.77 | 0.00 | 0.00 | 1.00 | 1.00 | 1.00 | 1.00 | 1.00 | 0.67 |
|  |  | Average | 0.02 | 0.02 | 0.04 | 0.24 | 0.00 | 0.00 | 0.83 | 1.00 | 1.00 | 1.00 | 1.00 | 0.23 |
|  |  | SD | 0.03 | 0.03 | 0.10 | 0.30 | 0.00 | 0.00 | 0.22 | 0.00 | 0.00 | 0.00 | 0.00 | 0.24 |
|  | T | N | 7 | 7 | 7 | 7 | 5 | 5 | 7 | 7 | 7 | 7 | 7 | 7 |
|  |  | Min | 0.00 | 0.00 | 0.00 | 0.17 | 0.00 | 0.00 | 0.81 | 1.00 | 1.00 | 0.99 | 0.99 | 0.13 |
|  |  | Max | 0.11 | 0.06 | 0.44 | 0.81 | 0.63 | 0.63 | 1.00 | 1.00 | 1.00 | 1.00 | 1.00 | 0.66 |
|  |  | Average | 0.04 | 0.02 | 0.15 | 0.44 | 0.35 | 0.24 | 0.94 | 1.00 | 1.00 | 1.00 | 1.00 | 0.36 |
|  |  | SD | 0.04 | 0.02 | 0.20 | 0.27 | 0.24 | 0.25 | 0.07 | 0.00 | 0.00 | 0.01 | 0.00 | 0.22 |
| Summer | R | N | 8 | 8 | 8 | 8 | 0 | 0 | 8 | 8 | 8 | 8 | 8 | 8 |
|  |  | Min | 0.00 | 0.00 | 0.00 | 0.00 |  |  | 0.00 | 0.00 | 0.25 | 0.00 | 0.39 | 0.01 |
|  |  | Max | 0.11 | 0.10 | 0.00 | 0.00 |  |  | 0.16 | 1.00 | 1.00 | 0.61 | 1.00 | 0.06 |
|  |  | Average | 0.05 | 0.04 | 0.00 | 0.00 |  |  | 0.06 | 0.39 | 0.76 | 0.08 | 0.64 | 0.04 |
|  |  | SD | 0.03 | 0.04 | 0.00 | 0.00 |  |  | 0.05 | 0.40 | 0.34 | 0.21 | 0.19 | 0.02 |
|  | UB | N | 8 | 8 | 8 | 7 | 0 | 0 | 6 | 4 | 2 | 6 | 7 | 8 |
|  |  | Min | 0.00 | 0.00 | 0.00 | 0.00 |  |  | 0.00 | 0.00 | 0.00 | 0.00 | 0.00 | 0.00 |
|  |  | Max | 0.00 | 0.00 | 0.00 | 0.00 |  |  | 1.00 | 0.00 | 0.00 | 0.00 | 0.00 | 0.00 |
|  |  | Average | 0.00 | 0.00 | 0.00 | 0.00 |  |  | 0.17 | 0.00 | 0.00 | 0.00 | 0.00 | 0.00 |
|  |  | SD | 0.00 | 0.00 | 0.00 | 0.00 |  |  | 0.41 | 0.00 | 0.00 | 0.00 | 0.00 | 0.00 |
|  | T | N | 8 | 8 | 8 | 8 | 0 | 7 | 8 | 8 | 8 | 8 | 7 | 8 |
|  |  | Min | 0.00 | 0.00 | 0.00 | 0.00 |  | 1.00 | 0.25 | 0.50 | 1.00 | 0.54 | 0.00 | 0.06 |
|  |  | Max | 1.00 | 0.03 | 0.00 | 0.17 |  | 1.00 | 1.00 | 1.00 | 1.00 | 0.80 | 0.00 | 0.23 |
|  |  | Average | 0.17 | 0.01 | 0.00 | 0.08 |  | 1.00 | 0.57 | 0.79 | 1.00 | 0.67 | 0.00 | 0.12 |
|  |  | SD | 0.34 | 0.01 | 0.00 | 0.06 |  | 0.00 | 0.30 | 0.23 | 0.00 | 0.10 | 0.00 | 0.06 |

Table 40: Gas (PUFs) and particle (QFFs) concentrations (in pg m^-3^) of NPAHs measured in individual samples at the rural site

|  |  | **Winter** | | | | | | | | **Summer** | | | | | | | |
| --- | --- | --- | --- | --- | --- | --- | --- | --- | --- | --- | --- | --- | --- | --- | --- | --- | --- |
|  |  | **2/13/17** | **2/14/17** | **2/15/17** | **2/16/17** | **2/17/17** | **2/18/17** | **2/19/17** | **2/20/17** | **7/6/17** | **7/7/17** | **7/8/17** | **7/9/17** | **7/10/17** | **7/11/17** | **7/12/17** | **7/13/17** |
| 1-NNAP | **PUFs** | 0.01 | 0.02 | 0.05 | 0.02 | 0.01 | 0.02 | 0.01 | 3.85E-03 | 0.01 | 0.01 | 0.01 | 0.01 | 4.63E-03 | 0.01 | 0.01 | 3.54E-03 |
|  | **QFFs** | <LOQ | <LOQ | 2.09E-04 | <LOQ | 1.01E-04 | 1.79E-04 | 9.81E-05 | <LOQ | <LOQ | <LOQ | <LOQ | <LOQ | <LOQ | <LOQ | <LOQ | <LOQ |
| 2-NNAP | **PUFs** | 0.01 | 0.02 | 0.03 | 0.01 | 0.01 | 0.01 | 0.01 | 3.07E-03 | 2.59E-03 | 2.79E-03 | 3.01E-03 | 3.13E-03 | 2.01E-03 | 1.69E-03 | 1.05E-03 | 2.83E-03 |
|  | **QFFs** | <LOQ | <LOQ | <LOQ | <LOQ | <LOQ | <LOQ | <LOQ | <LOQ | <LOQ | <LOQ | <LOQ | <LOQ | <LOQ | <LOQ | <LOQ | <LOQ |
| **5-NACE** | **PUFs** | <LOQ | 4.24E-04 | 6.75E-04 | 4.98E-04 | 5.36E-04 | 5.33E-04 | 3.87E-04 | <LOQ | 3.89E-03 | 4.29E-03 | 4.20E-03 | 0.01 | 4.16E-03 | 2.08E-03 | 2.06E-03 | 2.17E-03 |
|  | **QFFs** | <LOQ | <LOQ | <LOQ | <LOQ | <LOQ | <LOQ | <LOQ | <LOQ | <LOQ | <LOQ | <LOQ | <LOQ | <LOQ | <LOQ | <LOQ | <LOQ |
| 2-NFLN | **PUFs** | <LOQ | <LOQ | <LOQ | <LOQ | <LOQ | <LOQ | <LOQ | <LOQ | <LOQ | <LOQ | <LOQ | <LOQ | <LOQ | <LOQ | <LOQ | <LOQ |
|  | **QFFs** | <LOQ | <LOQ | <LOQ | <LOQ | <LOQ | <LOQ | <LOQ | <LOQ | <LOQ | <LOQ | <LOQ | <LOQ | <LOQ | <LOQ | <LOQ | <LOQ |
| 9-NANT | **PUFs** | 2.59E-03 | 0.01 | 0.02 | 0.01 | 0.02 | 0.02 | 0.01 | 0.01 | <LOQ | <LOQ | <LOQ | <LOQ | <LOQ | <LOQ | <LOQ | <LOQ |
|  | **QFFs** | 0.04 | 0.06 | 0.14 | 0.06 | 0.01 | 0.03 | 3.68E-03 | 9.38E-04 | <LOQ | <LOQ | <LOQ | <LOQ | <LOQ | <LOQ | <LOQ | <LOQ |
| 3-NPHE | **PUFs** | <LOQ | 9.33E-04 | 2.86E-03 | 2.37E-03 | 2.32E-03 | 4.39E-03 | 2.25E-03 | <LOQ | 6.76E-04 | 6.49E-04 | 6.79E-04 | 1.25E-03 | 6.65E-04 | 4.27E-04 | 3.59E-04 | 5.27E-04 |
|  | **QFFs** | <LOQ | 1.66E-03 | 2.60E-03 | 1.18E-03 | <LOQ | 8.67E-04 | <LOQ | <LOQ | <LOQ | <LOQ | <LOQ | <LOQ | <LOQ | <LOQ | <LOQ | <LOQ |
| 2-NFLT | **PUFs** | <LOQ | <LOQ | <LOQ | 5.17E-04 | 1.39E-03 | 4.25E-04 | <LOQ | <LOQ | 7.48E-04 | 8.78E-04 | 5.71E-04 | 9.27E-04 | 5.12E-04 | 3.22E-04 | 3.61E-04 | 3.33E-04 |
|  | **QFFs** | 0.05 | 0.03 | 0.05 | 0.04 | 0.01 | 0.01 | 4.68E-03 | 1.54E-03 | <LOQ | <LOQ | <LOQ | <LOQ | <LOQ | <LOQ | <LOQ | <LOQ |
| 3-NFLT | **PUFs** | <LOQ | <LOQ | <LOQ | <LOQ | <LOQ | <LOQ | <LOQ | <LOQ | <LOQ | <LOQ | <LOQ | <LOQ | <LOQ | <LOQ | <LOQ | <LOQ |
|  | **QFFs** | 3.84E-03 | 1.99E-03 | 0.01 | 4.85E-03 | 1.49E-03 | 2.04E-03 | 6.15E-04 | <LOQ | <LOQ | <LOQ | <LOQ | <LOQ | <LOQ | <LOQ | <LOQ | <LOQ |
| 1-NPYR | **PUFs** | <LOQ | <LOQ | 2.64E-04 | 1.03E-04 | <LOQ | <LOQ | 8.91E-05 | 6.86E-05 | 3.01E-05 | 8.79E-05 | 5.95E-05 | <LOQ | 2.34E-05 | <LOQ | 8.00E-05 | 5.52E-05 |
|  | **QFFs** | 4.67E-03 | 2.39E-03 | 2.82E-03 | 1.42E-03 | 3.58E-04 | 5.14E-04 | 3.07E-04 | <LOQ | <LOQ | <LOQ | <LOQ | <LOQ | <LOQ | <LOQ | <LOQ | <LOQ |
| 2-NPYR | **PUFs** | <LOQ | <LOQ | <LOQ | <LOQ | <LOQ | <LOQ | <LOQ | <LOQ | <LOQ | 5.55E-05 | <LOQ | 3.14E-05 | <LOQ | <LOQ | <LOQ | <LOQ |
|  | **QFFs** | 3.36E-03 | 2.62E-03 | 3.80E-03 | 1.98E-03 | 8.43E-04 | 1.55E-03 | 6.95E-04 | 2.85E-04 | <LOQ | <LOQ | <LOQ | <LOQ | <LOQ | <LOQ | <LOQ | <LOQ |
| 7-NBAA | **PUFs** | <LOQ | <LOQ | <LOQ | <LOQ | <LOQ | <LOQ | <LOQ | <LOQ | <LOQ | <LOQ | <LOQ | <LOQ | <LOQ | <LOQ | <LOQ | <LOQ |
|  | **QFFs** | 0.01 | 0.01 | 0.02 | 0.01 | 3.40E-03 | 0.01 | 1.71E-03 | <LOQ | <LOQ | <LOQ | <LOQ | <LOQ | <LOQ | <LOQ | <LOQ | <LOQ |
| 6-NCHR | **PUFs** | <LOQ | <LOQ | <LOQ | <LOQ | <LOQ | <LOQ | <LOQ | <LOQ | <LOQ | <LOQ | <LOQ | <LOQ | <LOQ | <LOQ | <LOQ | <LOQ |
|  | **QFFs** | 6.47E-04 | <LOQ | <LOQ | 3.84E-04 | <LOQ | <LOQ | <LOQ | <LOQ | <LOQ | <LOQ | <LOQ | <LOQ | <LOQ | <LOQ | <LOQ | <LOQ |
| 1,3-N2PYR | **PUFs** | <LOQ | <LOQ | <LOQ | <LOQ | <LOQ | <LOQ | <LOQ | <LOQ | <LOQ | <LOQ | <LOQ | <LOQ | <LOQ | <LOQ | <LOQ | <LOQ |
|  | **QFFs** | <LOQ | <LOQ | <LOQ | <LOQ | <LOQ | <LOQ | <LOQ | <LOQ | <LOQ | <LOQ | <LOQ | <LOQ | <LOQ | <LOQ | <LOQ | <LOQ |
| Σ9NPAHs | **PUFs** | 0.02 | 0.05 | 0.10 | 0.05 | 0.05 | 0.05 | 0.03 | 0.02 | 0.01 | 0.02 | 0.01 | 0.01 | 0.01 | 0.01 | 0.01 | 0.01 |
|  | **QFFs** | 0.10 | 0.10 | 0.23 | 0.11 | 0.02 | 0.05 | 0.01 | 2.77E-03 | <LOQ | <LOQ | <LOQ | <LOQ | <LOQ | <LOQ | <LOQ | <LOQ |

Table 41: Gas (PUFs) and particle (QFFs) concentrations (in pg m^-3^) of OPAHs measured in individual samples at the rural site

|  |  | **Winter** | | | | | | | | **Summer** | | | | | | | |
| --- | --- | --- | --- | --- | --- | --- | --- | --- | --- | --- | --- | --- | --- | --- | --- | --- | --- |
|  |  | **2/13/17** | **2/14/17** | **2/15/17** | **2/16/17** | **2/17/17** | **2/18/17** | **2/19/17** | **2/20/17** | **7/6/17** | **7/7/17** | **7/8/17** | **7/9/17** | **7/10/17** | **7/11/17** | **7/12/17** | **7/13/17** |
| 1,4-O2NAP | **PUFs** | 2.12 | 3.21 | 3.53 | 1.55 | 0.48 | 0.94 | 0.94 | 0.15 | 0.08 | 0.11 | 0.08 | 0.07 | 0.06 | 0.07 | 0.08 | 0.12 |
|  | **QFFs** | 0.01 | 0.01 | 0.01 | 4.94E-03 | <LOQ | <LOQ | <LOQ | <LOQ | 0.01 | 4.49E-03 | 4.18E-03 | 3.22E-03 | 3.33E-03 | 4.70E-03 | <LOQ | 3.72E-03 |
| 1(CHO)NAP | **PUFs** | 0.55 | 1.47 | 2.07 | 0.82 | 0.37 | 0.72 | 0.48 | 0.14 | 0.02 | 0.02 | 0.03 | 0.02 | 0.01 | 0.03 | 0.01 | 0.05 |
|  | **QFFs** | 0.01 | 0.01 | 0.01 | <LOQ | <LOQ | <LOQ | <LOQ | <LOQ | 2.62E-03 | 1.27E-03 | 1.24E-03 | <LOQ | 1.23E-03 | 1.21E-03 | <LOQ | <LOQ |
| 9-OFLN | **PUFs** | 0.42 | 0.58 | 0.79 | 0.46 | 0.50 | 0.56 | 0.48 | 0.19 | 0.03 | 0.04 | 0.03 | 0.04 | 0.03 | 0.03 | 0.05 | 0.04 |
|  | **QFFs** | 0.09 | <LOQ | 0.06 | <LOQ | <LOQ | <LOQ | <LOQ | <LOQ | <LOQ | <LOQ | <LOQ | <LOQ | <LOQ | <LOQ | <LOQ | <LOQ |
| 9,10-O2ANT | **PUFs** | 0.06 | 0.08 | 0.86 | 0.81 | 0.37 | 0.21 | 0.18 | 0.22 | 0.08 | 0.07 | 0.07 | 0.07 | 0.06 | 0.03 | 0.04 | 0.06 |
|  | **QFFs** | 0.51 | 0.40 | 0.42 | 0.26 | 0.25 | 0.35 | 0.19 | 0.03 | <LOQ | <LOQ | <LOQ | <LOQ | <LOQ | <LOQ | <LOQ | <LOQ |
| 1,4-O2ANT | **PUFs** | 9.21E-04 | 1.83E-03 | 3.13E-03 | 1.93E-03 | 2.16E-03 | 2.06E-03 | 1.64E-03 | 1.12E-03 | <LOQ | <LOQ | <LOQ | <LOQ | <LOQ | <LOQ | <LOQ | <LOQ |
|  | **QFFs** | 4.77E-03 | 4.11E-03 | 4.34E-03 | 2.22E-03 | 6.24E-04 | 1.83E-03 | <LOQ | <LOQ | <LOQ | <LOQ | <LOQ | <LOQ | <LOQ | <LOQ | <LOQ | <LOQ |
| 9,10-O2PHE | **PUFs** | 0.60 | 0.65 | <LOQ | 1.05 | 1.23 | 0.75 | <LOQ | 0.45 | <LOQ | <LOQ | <LOQ | <LOQ | <LOQ | <LOQ | <LOQ | <LOQ |
|  | **QFFs** | 0.28 | 0.35 | 0.58 | 0.20 | 0.09 | 0.18 | 0.05 | <LOQ | <LOQ | <LOQ | <LOQ | <LOQ | <LOQ | <LOQ | <LOQ | <LOQ |
| BaOFLN | **PUFs** | <LOQ | 3.28E-03 | 0.01 | 0.02 | 0.02 | 0.01 | 0.01 | 0.01 | 0.01 | 0.01 | 0.01 | 0.01 | 0.01 | 4.17E-03 | 4.31E-03 | 0.01 |
|  | **QFFs** | 0.16 | 0.10 | 0.12 | 0.06 | 0.02 | 0.04 | 0.02 | 0.01 | <LOQ | <LOQ | 7.57E-04 | 6.19E-04 | 4.67E-04 | 3.40E-04 | <LOQ | 1.05E-03 |
| BbOFLN | **PUFs** | <LOQ | <LOQ | 3.24E-03 | <LOQ | 0.01 | <LOQ | <LOQ | <LOQ | 3.29E-03 | 0.01 | 4.95E-03 | 0.01 | 4.95E-03 | <LOQ | <LOQ | 3.24E-03 |
|  | **QFFs** | 0.19 | 0.14 | 0.19 | 0.10 | 0.04 | 0.07 | 0.03 | 0.01 | 6.76E-04 | <LOQ | 1.71E-03 | 7.87E-04 | 7.97E-04 | 9.19E-04 | 6.23E-04 | 2.43E-03 |
| BAN | **PUFs** | <LOQ | <LOQ | <LOQ | <LOQ | 0.01 | <LOQ | <LOQ | <LOQ | <LOQ | 4.03E-03 | 3.25E-03 | 3.55E-03 | <LOQ | <LOQ | <LOQ | <LOQ |
|  | **QFFs** | 0.24 | 0.21 | 0.24 | 0.19 | 0.08 | 0.17 | 0.08 | 0.02 | 1.51E-03 | 1.33E-03 | 2.77E-03 | 1.78E-03 | 1.50E-03 | 2.42E-03 | 9.94E-04 | 0.01 |
| 7,12-O2BAA | **PUFs** | <LOQ | <LOQ | 1.04E-03 | <LOQ | 4.39E-03 | 1.41E-03 | <LOQ | <LOQ | 1.46E-03 | 2.39E-03 | 1.50E-03 | 3.05E-03 | 1.86E-03 | 1.10E-03 | 1.50E-03 | 1.44E-03 |
|  | **QFFs** | 0.08 | 0.05 | 0.07 | 0.04 | 0.02 | 0.03 | 0.02 | 0.01 | <LOQ | <LOQ | <LOQ | <LOQ | <LOQ | <LOQ | <LOQ | 2.21E-03 |
| 5,12-O2NAC | **PUFs** | <LOQ | 2.23E-04 | <LOQ | <LOQ | <LOQ | <LOQ | <LOQ | <LOQ | 7.54E-05 | 9.97E-05 | 6.51E-05 | <LOQ | 9.07E-05 | 1.02E-04 | 1.09E-04 | 1.17E-04 |
|  | **QFFs** | 0.03 | 0.02 | 0.02 | 0.01 | 4.82E-03 | 0.01 | 4.49E-03 | 1.46E-03 | 7.17E-05 | 1.17E-04 | 2.00E-04 | 1.54E-04 | 1.79E-04 | 1.49E-04 | 6.96E-05 | 2.96E-04 |
| **Σ11OPAHs** | **PUFs** | 3.76 | 6.00 | 7.26 | 4.71 | 2.99 | 3.20 | 2.09 | 1.16 | 0.23 | 0.26 | 0.23 | 0.22 | 0.17 | 0.17 | 0.19 | 0.28 |
|  | **QFFs** | 1.61 | 1.29 | 1.72 | 0.88 | 0.50 | 0.86 | 0.39 | 0.07 | 0.01 | 0.01 | 0.01 | 0.01 | 0.01 | 0.01 | 1.69E-03 | 0.02 |

Table 42: Gas (PUFs) and particle (QFFs) concentrations (in pg m^-3^) of NPAHs measured in individual samples at the urban background site

|  |  | **Winter** | | | | | | | **Summer** | | | | | | | |
| --- | --- | --- | --- | --- | --- | --- | --- | --- | --- | --- | --- | --- | --- | --- | --- | --- |
|  |  | **2/13/17** | **2/14/17** | **2/15/17** | **2/16/17** | **2/17/17** | **2/18/17** | **2/19/17** | **7/6/17** | **7/7/17** | **7/8/17** | **7/9/17** | **7/10/17** | **7/11/17** | **7/12/17** | **7/13/17** |
| 1-NNAP | **PUFs** | 0.06 | 0.07 | 0.09 | 0.07 | 0.07 | 0.05 | 0.07 | 0.08 | 0.07 | 0.07 | 0.07 | 0.06 | 0.05 | 0.05 | 0.05 |
|  | **QFFs** | <LOQ | <LOQ | <LOQ | <LOQ | <LOQ | <LOQ | 6.52E-04 | <LOQ | <LOQ | <LOQ | <LOQ | <LOQ | <LOQ | <LOQ | <LOQ |
| 2-NNAP | **PUFs** | 0.03 | 0.03 | 0.04 | 0.05 | 0.03 | 0.02 | 0.03 | 0.01 | 0.02 | 0.01 | 0.01 | 0.01 | 0.01 | 0.01 | 0.01 |
|  | **QFFs** | <LOQ | <LOQ | <LOQ | <LOQ | <LOQ | <LOQ | <LOQ | <LOQ | <LOQ | <LOQ | <LOQ | <LOQ | <LOQ | <LOQ | <LOQ |
| **5-NACE** | **PUFs** | <LOQ | <LOQ | <LOQ | <LOQ | <LOQ | <LOQ | <LOQ | 0.03 | 0.02 | 0.02 | 0.04 | 0.03 | 0.02 | 0.01 | 0.01 |
|  | **QFFs** | <LOQ | <LOQ | <LOQ | <LOQ | <LOQ | <LOQ | <LOQ | <LOQ | <LOQ | <LOQ | <LOQ | <LOQ | <LOQ | <LOQ | <LOQ |
| 2-NFLN | **PUFs** | <LOQ | <LOQ | <LOQ | <LOQ | <LOQ | <LOQ | <LOQ | <LOQ | <LOQ | <LOQ | <LOQ | <LOQ | <LOQ | <LOQ | <LOQ |
|  | **QFFs** | <LOQ | <LOQ | <LOQ | <LOQ | <LOQ | <LOQ | <LOQ | <LOQ | <LOQ | <LOQ | <LOQ | <LOQ | <LOQ | <LOQ | <LOQ |
| 9-NANT | **PUFs** | 0.02 | 0.02 | 0.02 | 0.03 | 0.02 | 0.03 | 0.03 | <LOQ | <LOQ | <LOQ | <LOQ | <LOQ | <LOQ | <LOQ | <LOQ |
|  | **QFFs** | 0.40 | 0.10 | 0.16 | 0.01 | 0.01 | 0.01 | 0.02 | <LOQ | <LOQ | <LOQ | <LOQ | <LOQ | <LOQ | <LOQ | <LOQ |
| 3-NPHE | **PUFs** | <LOQ | <LOQ | <LOQ | <LOQ | <LOQ | <LOQ | <LOQ | <LOQ | <LOQ | <LOQ | <LOQ | <LOQ | <LOQ | <LOQ | <LOQ |
|  | **QFFs** | <LOQ | <LOQ | <LOQ | <LOQ | <LOQ | <LOQ | <LOQ | <LOQ | <LOQ | <LOQ | <LOQ | <LOQ | <LOQ | <LOQ | <LOQ |
| 2-NFLT | **PUFs** | <LOQ | <LOQ | <LOQ | <LOQ | <LOQ | <LOQ | <LOQ | 2.05E-03 | 3.72E-03 | 2.71E-03 | 3.29E-03 | 1.89E-03 | 1.64E-03 | 3.34E-03 | 1.94E-03 |
|  | **QFFs** | 0.37 | 0.16 | 0.23 | 0.06 | 0.03 | 0.02 | 0.03 | <LOQ | <LOQ | <LOQ | <LOQ | <LOQ | <LOQ | <LOQ | <LOQ |
| 3-NFLT | **PUFs** | <LOQ | <LOQ | <LOQ | <LOQ | <LOQ | <LOQ | <LOQ | <LOQ | <LOQ | <LOQ | <LOQ | <LOQ | <LOQ | <LOQ | <LOQ |
|  | **QFFs** | 0.07 | 0.03 | 0.03 | <LOQ | <LOQ | <LOQ | <LOQ | <LOQ | <LOQ | <LOQ | <LOQ | <LOQ | <LOQ | <LOQ | <LOQ |
| 1-NPYR | **PUFs** | <LOQ | <LOQ | <LOQ | 7.79E-04 | <LOQ | <LOQ | <LOQ | 6.76E-04 | 1.40E-03 | 1.58E-03 | 9.12E-04 | 5.13E-04 | 9.84E-04 | 8.76E-04 | 1.31E-03 |
|  | **QFFs** | 0.08 | 0.04 | 0.03 | 0.01 | 0.01 | 4.09E-03 | 4.78E-03 | <LOQ | <LOQ | <LOQ | <LOQ | <LOQ | <LOQ | <LOQ | <LOQ |
| 2-NPYR | **PUFs** | <LOQ | <LOQ | <LOQ | <LOQ | <LOQ | <LOQ | <LOQ | <LOQ | <LOQ | <LOQ | <LOQ | <LOQ | <LOQ | <LOQ | <LOQ |
|  | **QFFs** | 0.10 | 0.05 | 0.05 | 0.01 | 0.02 | 0.01 | 0.01 | <LOQ | <LOQ | <LOQ | <LOQ | <LOQ | <LOQ | <LOQ | <LOQ |
| 7-NBAA | **PUFs** | <LOQ | <LOQ | <LOQ | <LOQ | <LOQ | <LOQ | <LOQ | <LOQ | <LOQ | <LOQ | <LOQ | <LOQ | <LOQ | <LOQ | <LOQ |
|  | **QFFs** | 0.08 | 0.06 | 0.06 | <LOQ | <LOQ | <LOQ | <LOQ | <LOQ | <LOQ | <LOQ | <LOQ | <LOQ | <LOQ | <LOQ | <LOQ |
| 6-NCHR | **PUFs** | <LOQ | <LOQ | <LOQ | <LOQ | <LOQ | <LOQ | <LOQ | <LOQ | <LOQ | <LOQ | <LOQ | <LOQ | <LOQ | <LOQ | <LOQ |
|  | **QFFs** | <LOQ | <LOQ | <LOQ | <LOQ | <LOQ | <LOQ | <LOQ | <LOQ | <LOQ | <LOQ | <LOQ | <LOQ | <LOQ | <LOQ | <LOQ |
| 1,3-N2PYR | **PUFs** | <LOQ | <LOQ | <LOQ | <LOQ | <LOQ | <LOQ | <LOQ | <LOQ | <LOQ | <LOQ | <LOQ | <LOQ | <LOQ | <LOQ | <LOQ |
|  | **QFFs** | <LOQ | <LOQ | <LOQ | <LOQ | <LOQ | <LOQ | <LOQ | <LOQ | <LOQ | <LOQ | <LOQ | <LOQ | <LOQ | <LOQ | <LOQ |
| Σ9NPAHs | **PUFs** | 0.10 | 0.12 | 0.15 | 0.14 | 0.12 | 0.10 | 0.13 | 0.09 | 0.09 | 0.09 | 0.09 | 0.07 | 0.06 | 0.06 | 0.06 |
|  | **QFFs** | 1.10 | 0.45 | 0.56 | 0.08 | 0.06 | 0.05 | 0.07 | <LOQ | <LOQ | <LOQ | <LOQ | <LOQ | <LOQ | <LOQ | <LOQ |

Table 43: Gas (PUFs) and particle (QFFs) concentrations (in pg m^-3^) of OPAHs measured in individual samples at the urban background site

|  |  | **Winter** | | | | | | | **Summer** | | | | | | | |
| --- | --- | --- | --- | --- | --- | --- | --- | --- | --- | --- | --- | --- | --- | --- | --- | --- |
|  |  | **2/13/17** | **2/14/17** | **2/15/17** | **2/16/17** | **2/17/17** | **2/18/17** | **2/19/17** | **7/6/17** | **7/7/17** | **7/8/17** | **7/9/17** | **7/10/17** | **7/11/17** | **7/12/17** | **7/13/17** |
| 1,4-O2NAP | **PUFs** | 6.93 | 6.36 | 4.93 | 4.22 | 1.59 | 1.47 | 2.66 | 2.14 | 0.55 | 0.95 | 0.95 | 0.30 | 0.30 | 0.46 | 0.40 |
|  | **QFFs** | 0.55 | 0.30 | 0.21 | <LOQ | <LOQ | <LOQ | <LOQ | <LOQ | <LOQ | <LOQ | <LOQ | <LOQ | <LOQ | <LOQ | <LOQ |
| 1(CHO)NAP | **PUFs** | 1.37 | 1.87 | 2.26 | 1.49 | 1.46 | 1.06 | 1.21 | 0.22 | 0.17 | 0.27 | 0.26 | 0.11 | 0.18 | 0.18 | 0.21 |
|  | **QFFs** | 0.14 | 0.05 | 0.05 | <LOQ | <LOQ | <LOQ | <LOQ | <LOQ | <LOQ | <LOQ | <LOQ | <LOQ | <LOQ | <LOQ | <LOQ |
| 9-OFLN | **PUFs** | 2.80 | 2.78 | 2.56 | 2.51 | 2.14 | 1.65 | 1.87 | 0.36 | 0.39 | 0.39 | 0.40 | 0.33 | 0.39 | 0.35 | 0.39 |
|  | **QFFs** | 1.05 | <LOQ | <LOQ | <LOQ | <LOQ | <LOQ | <LOQ | <LOQ | <LOQ | <LOQ | <LOQ | <LOQ | <LOQ | <LOQ | <LOQ |
| 9,10-O2ANT | **PUFs** | 2.38 | 1.86 | 2.00 | 2.08 | 1.54 | 1.60 | 1.62 | 0.37 | 0.41 | 0.40 | 0.48 | 0.37 | 0.30 | <LOQ | 0.35 |
|  | **QFFs** | 7.82 | 1.69 | 1.24 | 0.15 | <LOQ | <LOQ | 0.15 | <LOQ | <LOQ | <LOQ | <LOQ | <LOQ | <LOQ | <LOQ | <LOQ |
| 1,4-O2ANT | **PUFs** | 0.02 | 0.04 | <LOQ | <LOQ | <LOQ | 0.02 | <LOQ | <LOQ | <LOQ | <LOQ | <LOQ | <LOQ | <LOQ | <LOQ | <LOQ |
|  | **QFFs** | <LOQ | <LOQ | <LOQ | <LOQ | <LOQ | <LOQ | <LOQ | <LOQ | <LOQ | <LOQ | <LOQ | <LOQ | <LOQ | <LOQ | <LOQ |
| 9,10-O2PHE | **PUFs** | 1.24 | 2.91 | 3.80 | 3.40 | 2.60 | <LOQ | 3.05 | <LOQ | <LOQ | <LOQ | <LOQ | <LOQ | <LOQ | <LOQ | <LOQ |
|  | **QFFs** | <LOQ | <LOQ | <LOQ | <LOQ | <LOQ | <LOQ | <LOQ | <LOQ | <LOQ | <LOQ | <LOQ | <LOQ | <LOQ | <LOQ | <LOQ |
| BaOFLN | **PUFs** | <LOQ | <LOQ | <LOQ | 0.06 | <LOQ | 0.06 | 0.06 | 0.05 | 0.04 | 0.04 | 0.05 | <LOQ | <LOQ | 0.04 | <LOQ |
|  | **QFFs** | 4.02 | 1.11 | 0.87 | 0.11 | 0.08 | 0.07 | 0.10 | <LOQ | <LOQ | <LOQ | <LOQ | <LOQ | 0.01 | <LOQ | <LOQ |
| BbOFLN | **PUFs** | <LOQ | <LOQ | <LOQ | <LOQ | <LOQ | <LOQ | <LOQ | 0.04 | 0.04 | <LOQ | 0.05 | <LOQ | <LOQ | 0.04 | <LOQ |
|  | **QFFs** | 6.85 | 2.05 | 1.50 | 0.14 | 0.13 | 0.10 | 0.15 | <LOQ | <LOQ | <LOQ | <LOQ | <LOQ | <LOQ | <LOQ | <LOQ |
| BAN | **PUFs** | <LOQ | <LOQ | <LOQ | <LOQ | <LOQ | <LOQ | <LOQ | 0.05 | <LOQ | <LOQ | <LOQ | <LOQ | <LOQ | 0.05 | <LOQ |
|  | **QFFs** | 7.54 | 3.50 | 2.89 | 0.32 | 0.30 | 0.27 | 0.34 | <LOQ | <LOQ | <LOQ | <LOQ | <LOQ | <LOQ | <LOQ | <LOQ |
| 7,12-O2BAA | **PUFs** | <LOQ | <LOQ | <LOQ | <LOQ | <LOQ | <LOQ | <LOQ | <LOQ | 0.02 | 0.01 | 0.01 | 0.01 | <LOQ | 0.02 | 0.01 |
|  | **QFFs** | 1.36 | 0.52 | 0.43 | 0.06 | 0.05 | 0.05 | 0.06 | <LOQ | <LOQ | <LOQ | <LOQ | <LOQ | <LOQ | <LOQ | <LOQ |
| 5,12-O2NAC | **PUFs** | <LOQ | <LOQ | <LOQ | <LOQ | <LOQ | <LOQ | <LOQ | 5.07E-04 | 7.07E-04 | <LOQ | 9.06E-04 | 7.97E-04 | 7.79E-04 | 8.70E-04 | 6.52E-04 |
|  | **QFFs** | 0.99 | 0.27 | 0.17 | 0.02 | 0.02 | 0.02 | 0.02 | <LOQ | <LOQ | <LOQ | <LOQ | <LOQ | <LOQ | <LOQ | <LOQ |
| **Σ11OPAHs** | **PUFs** | 14.73 | 15.82 | 15.56 | 13.76 | 9.32 | 5.85 | 10.46 | 3.23 | 1.63 | 2.07 | 2.20 | 1.12 | 1.18 | 1.15 | 1.36 |
|  | **QFFs** | 30.33 | 9.48 | 7.35 | 0.81 | 0.57 | 0.50 | 0.82 | <LOQ | <LOQ | <LOQ | <LOQ | <LOQ | 0.01 | <LOQ | <LOQ |

Table 44: Gas (PUFs) and particle (QFFs) concentrations (in pg m^-3^) of NPAHs measured in individual samples at the traffic site

|  |  | **Winter** | | | | | | | **Summer** | | | | | | | |
| --- | --- | --- | --- | --- | --- | --- | --- | --- | --- | --- | --- | --- | --- | --- | --- | --- |
|  |  | **2/13/17** | **2/14/17** | **2/15/17** | **2/16/17** | **2/17/17** | **2/18/17** | **2/19/17** | **7/6/17** | **7/7/17** | **7/8/17** | **7/9/17** | **7/10/17** | **7/11/17** | **7/12/17** | **7/13/17** |
| 1-NNAP | **PUFs** | 0.25 | 0.18 | 0.21 | 0.14 | 0.13 | 0.11 | 0.05 | 0.04 | 0.03 | 0.03 | 0.04 | 0.03 | 0.03 | 0.04 | 0.03 |
|  | **QFFs** | 0.01 | 3.14E-03 | 4.47E-03 | 7.80E-04 | 1.17E-03 | 6.05E-04 | 9.23E-04 | <LOQ | <LOQ | <LOQ | <LOQ | <LOQ | <LOQ | <LOQ | <LOQ |
| 2-NNAP | **PUFs** | 0.08 | 0.05 | 0.06 | 0.05 | 0.04 | 0.03 | 0.01 | 0.01 | 0.01 | 0.01 | 0.01 | 0.01 | 0.01 | 0.01 | 0.01 |
|  | **QFFs** | 3.13E-03 | 1.65E-03 | 1.29E-03 | 4.49E-04 | 4.19E-04 | 2.95E-04 | 3.50E-04 | <LOQ | <LOQ | 1.38E-04 | 1.48E-04 | 2.00E-04 | 2.61E-04 | <LOQ | 1.63E-04 |
| **5-NACE** | **PUFs** | 4.30E-03 | 3.91E-03 | 0.01 | 4.35E-03 | 3.05E-03 | 2.43E-03 | 9.22E-04 | 0.03 | 0.03 | 0.03 | 0.05 | 0.04 | 0.02 | 0.01 | 0.01 |
|  | **QFFs** | <LOQ | <LOQ | <LOQ | <LOQ | <LOQ | <LOQ | <LOQ | <LOQ | <LOQ | <LOQ | <LOQ | <LOQ | <LOQ | <LOQ | <LOQ |
| 2-NFLN | **PUFs** | <LOQ | <LOQ | <LOQ | <LOQ | <LOQ | <LOQ | <LOQ | <LOQ | <LOQ | <LOQ | <LOQ | <LOQ | <LOQ | <LOQ | <LOQ |
|  | **QFFs** | <LOQ | <LOQ | <LOQ | <LOQ | <LOQ | <LOQ | 4.13E-04 | <LOQ | <LOQ | <LOQ | <LOQ | <LOQ | <LOQ | <LOQ | <LOQ |
| 9-NANT | **PUFs** | 0.04 | 0.04 | 0.05 | 0.06 | 0.04 | 0.06 | 0.03 | <LOQ | <LOQ | <LOQ | <LOQ | <LOQ | <LOQ | <LOQ | <LOQ |
|  | **QFFs** | 0.82 | 1.65 | 1.71 | 0.17 | 0.08 | 0.26 | 0.30 | <LOQ | <LOQ | <LOQ | <LOQ | <LOQ | <LOQ | <LOQ | <LOQ |
| 3-NPHE | **PUFs** | 2.76E-03 | 2.48E-03 | 3.56E-03 | 3.73E-03 | 4.02E-03 | 0.01 | 3.14E-03 | 4.96E-03 | 0.01 | 0.01 | 0.01 | 0.01 | 2.18E-03 | 2.76E-03 | 1.40E-03 |
|  | **QFFs** | 0.04 | 0.04 | 0.05 | 0.01 | 4.22E-03 | 0.01 | 0.01 | 3.16E-04 | 6.83E-04 | 3.96E-04 | 5.51E-04 | 1.44E-03 | 1.02E-03 | 5.24E-04 | 5.77E-04 |
| 2-NFLT | **PUFs** | 5.53E-04 | <LOQ | 7.33E-04 | 7.09E-04 | <LOQ | 5.65E-04 | 5.04E-04 | 5.76E-04 | 7.92E-04 | 6.75E-04 | 6.37E-04 | 4.99E-04 | 3.67E-04 | 4.05E-04 | 4.52E-04 |
|  | **QFFs** | 0.84 | 0.37 | 0.50 | 0.10 | 0.05 | 0.04 | 0.06 | 1.10E-03 | 1.96E-03 | 8.93E-04 | 1.60E-03 | 3.10E-03 | 6.37E-04 | 6.48E-04 | 3.18E-04 |
| 3-NFLT | **PUFs** | <LOQ | <LOQ | <LOQ | <LOQ | <LOQ | <LOQ | <LOQ | <LOQ | <LOQ | <LOQ | <LOQ | <LOQ | <LOQ | <LOQ | <LOQ |
|  | **QFFs** | 0.13 | 0.06 | 0.08 | 0.01 | 0.01 | 4.35E-03 | 0.01 | 5.41E-04 | 3.08E-04 | 3.71E-04 | 4.84E-04 | 8.18E-04 | 2.54E-04 | 2.20E-04 | 1.49E-04 |
| 1-NPYR | **PUFs** | <LOQ | 1.29E-04 | 1.58E-04 | <LOQ | <LOQ | <LOQ | <LOQ | 2.45E-04 | 3.24E-04 | 2.57E-04 | <LOQ | <LOQ | <LOQ | 1.75E-04 | 3.50E-04 |
|  | **QFFs** | 0.19 | 0.09 | 0.08 | 0.02 | 0.02 | 0.01 | 0.01 | 8.99E-04 | 1.34E-03 | 1.23E-03 | 9.27E-04 | 2.50E-03 | 1.90E-03 | 1.54E-03 | 2.21E-03 |
| 2-NPYR | **PUFs** | <LOQ | <LOQ | <LOQ | <LOQ | <LOQ | <LOQ | <LOQ | <LOQ | <LOQ | <LOQ | <LOQ | <LOQ | <LOQ | <LOQ | <LOQ |
|  | **QFFs** | 0.19 | 0.12 | 0.14 | 0.02 | 0.02 | 0.01 | 0.02 | 1.40E-04 | 1.38E-04 | 2.29E-04 | 1.02E-04 | 2.86E-04 | 1.43E-04 | 1.24E-04 | 6.81E-05 |
| 7-NBAA | **PUFs** | <LOQ | <LOQ | <LOQ | <LOQ | <LOQ | <LOQ | <LOQ | <LOQ | <LOQ | <LOQ | <LOQ | <LOQ | <LOQ | <LOQ | <LOQ |
|  | **QFFs** | 0.20 | 0.27 | 0.44 | 0.02 | 0.01 | 0.03 | 0.03 | 1.73E-04 | 2.00E-04 | 1.04E-03 | 2.58E-04 | 3.95E-04 | 2.87E-04 | 1.11E-04 | 2.50E-04 |
| 6-NCHR | **PUFs** | <LOQ | <LOQ | <LOQ | <LOQ | <LOQ | <LOQ | <LOQ | <LOQ | <LOQ | <LOQ | <LOQ | <LOQ | <LOQ | <LOQ | <LOQ |
|  | **QFFs** | <LOQ | <LOQ | <LOQ | <LOQ | <LOQ | <LOQ | <LOQ | <LOQ | <LOQ | <LOQ | <LOQ | <LOQ | <LOQ | <LOQ | <LOQ |
| 1,3-N2PYR | **PUFs** | <LOQ | <LOQ | <LOQ | <LOQ | <LOQ | <LOQ | <LOQ | <LOQ | <LOQ | <LOQ | <LOQ | <LOQ | <LOQ | <LOQ | <LOQ |
|  | **QFFs** | <LOQ | <LOQ | <LOQ | 5.76E-05 | 9.44E-05 | <LOQ | 7.28E-05 | <LOQ | <LOQ | <LOQ | <LOQ | <LOQ | <LOQ | <LOQ | <LOQ |
| Σ9NPAHs | **PUFs** | 0.36 | 0.27 | 0.32 | 0.26 | 0.21 | 0.20 | 0.09 | 0.06 | 0.05 | 0.05 | 0.06 | 0.04 | 0.04 | 0.05 | 0.04 |
|  | **QFFs** | 2.42 | 2.59 | 3.01 | 0.34 | 0.19 | 0.36 | 0.44 | 3.17E-03 | 4.63E-03 | 4.29E-03 | 4.07E-03 | 0.01 | 4.50E-03 | 3.16E-03 | 3.73E-03 |

Table 45: Gas (PUFs) and particle (QFFs) concentrations (in pg m^-3^) of OPAHs measured in individual samples at the traffic site

|  |  | **Winter** | | | | | | | **Summer** | | | | | | | |
| --- | --- | --- | --- | --- | --- | --- | --- | --- | --- | --- | --- | --- | --- | --- | --- | --- |
|  |  | **2/13/17** | **2/14/17** | **2/15/17** | **2/16/17** | **2/17/17** | **2/18/17** | **2/19/17** | **7/6/17** | **7/7/17** | **7/8/17** | **7/9/17** | **7/10/17** | **7/11/17** | **7/12/17** | **7/13/17** |
| 1,4-O2NAP | **PUFs** | 4.33 | 3.63 | 3.00 | 2.86 | 1.10 | 1.28 | 1.01 | 0.47 | 0.03 | 0.14 | 0.10 | 0.03 | <LOQ | 0.11 | 0.10 |
|  | **QFFs** | 0.53 | 0.21 | 0.22 | 0.01 | 0.01 | 0.01 | 0.01 | 3.82E-03 | 4.77E-03 | 3.94E-03 | 3.68E-03 | 0.01 | 3.89E-03 | <LOQ | 3.64E-03 |
| 1(CHO)NAP | **PUFs** | 3.01 | 2.69 | 3.18 | 2.05 | 1.36 | 1.23 | 0.56 | 0.09 | 0.08 | 0.09 | 0.08 | 0.05 | 0.09 | 0.08 | 0.10 |
|  | **QFFs** | 0.20 | 0.10 | 0.14 | 3.45E-03 | 3.36E-03 | 4.41E-03 | 4.36E-03 | <LOQ | <LOQ | <LOQ | <LOQ | 1.51E-03 | 1.51E-03 | <LOQ | 1.25E-03 |
| 9-OFLN | **PUFs** | 1.00 | 0.93 | 0.94 | 0.92 | 0.87 | 0.80 | 0.66 | 0.07 | 0.07 | 0.06 | 0.06 | 0.05 | 0.05 | 0.04 | 0.05 |
|  | **QFFs** | 0.79 | 0.46 | 0.39 | <LOQ | <LOQ | <LOQ | <LOQ | <LOQ | <LOQ | <LOQ | <LOQ | <LOQ | <LOQ | <LOQ | <LOQ |
| 9,10-O2ANT | **PUFs** | 0.92 | 1.41 | 1.53 | 1.77 | 1.25 | 1.21 | 0.81 | 0.09 | 0.07 | 0.08 | 0.08 | 0.05 | 0.06 | 0.06 | 0.07 |
|  | **QFFs** | 3.96 | 2.89 | 3.38 | 0.37 | 0.30 | 0.36 | 0.38 | 0.01 | 0.01 | <LOQ | 0.01 | 0.01 | 0.01 | <LOQ | 0.01 |
| 1,4-O2ANT | **PUFs** | <LOQ | <LOQ | 0.01 | 2.39E-03 | 0.01 | 0.01 | 4.22E-03 | <LOQ | <LOQ | <LOQ | <LOQ | <LOQ | <LOQ | <LOQ | <LOQ |
|  | **QFFs** | <LOQ | <LOQ | <LOQ | 4.16E-03 | 3.91E-03 | 2.76E-03 | 3.84E-03 | <LOQ | <LOQ | <LOQ | <LOQ | <LOQ | <LOQ | <LOQ | <LOQ |
| 9,10-O2PHE | **PUFs** | <LOQ | 3.48 | <LOQ | 0.23 | 0.44 | 3.92 | 1.62 | <LOQ | <LOQ | <LOQ | <LOQ | <LOQ | <LOQ | <LOQ | <LOQ |
|  | **QFFs** | <LOQ | <LOQ | <LOQ | 0.39 | 0.20 | 0.25 | 0.42 | 0.01 | 0.01 | <LOQ | 0.01 | 0.01 | 0.02 | 0.01 | 0.02 |
| BaOFLN | **PUFs** | 0.01 | 3.87E-03 | 0.01 | 0.03 | 0.01 | 0.03 | 0.02 | 0.01 | 0.01 | 0.01 | 0.01 | 0.01 | <LOQ | 3.09E-03 | <LOQ |
|  | **QFFs** | 3.17 | 2.14 | 2.23 | 0.22 | 0.17 | 0.12 | 0.19 | 0.01 | 0.01 | 4.89E-03 | 4.24E-03 | 0.01 | 0.01 | 0.01 | 0.01 |
| BbOFLN | **PUFs** | <LOQ | 3.60E-03 | <LOQ | <LOQ | <LOQ | <LOQ | <LOQ | 2.93E-03 | 2.74E-03 | 3.31E-03 | 2.79E-03 | <LOQ | <LOQ | <LOQ | <LOQ |
|  | **QFFs** | 3.28 | 2.70 | 0.25 | 0.24 | 0.24 | 0.19 | 0.24 | 0.01 | 3.26E-03 | 4.56E-03 | 2.79E-03 | 0.01 | 3.91E-03 | 4.14E-03 | 3.31E-03 |
| BAN | **PUFs** | <LOQ | <LOQ | <LOQ | <LOQ | <LOQ | <LOQ | <LOQ | <LOQ | <LOQ | <LOQ | <LOQ | <LOQ | <LOQ | <LOQ | <LOQ |
|  | **QFFs** | 3.19 | 2.46 | 3.33 | 0.25 | 0.26 | 0.23 | 0.25 | 0.01 | 0.01 | 0.01 | 4.19E-03 | 0.01 | 0.01 | 0.01 | 0.01 |
| 7,12-O2BAA | **PUFs** | <LOQ | 1.24E-03 | 1.40E-03 | <LOQ | <LOQ | <LOQ | 1.36E-03 | 2.27E-03 | 2.16E-03 | 1.77E-03 | 1.81E-03 | 1.06E-03 | 1.39E-03 | 1.90E-03 | 2.21E-03 |
|  | **QFFs** | 1.47 | 1.01 | 0.99 | 0.11 | 0.07 | 0.07 | 0.10 | 0.01 | 3.92E-03 | 0.01 | 2.70E-03 | 4.17E-03 | 2.56E-03 | 2.51E-03 | 2.61E-03 |
| 5,12-O2NAC | **PUFs** | 4.61E-04 | 2.86E-04 | <LOQ | 2.85E-04 | <LOQ | <LOQ | 1.70E-04 | 2.19E-04 | 1.33E-04 | 1.23E-04 | 1.22E-04 | <LOQ | 1.41E-04 | 1.25E-04 | 1.54E-04 |
|  | **QFFs** | 1.51 | 0.53 | 0.57 | 0.03 | 0.02 | 0.02 | 0.03 | <LOQ | <LOQ | <LOQ | <LOQ | <LOQ | <LOQ | <LOQ | <LOQ |
| **Σ11OPAHs** | **PUFs** | 9.26 | 12.15 | 8.68 | 7.86 | 5.06 | 8.46 | 4.69 | 0.74 | 0.26 | 0.39 | 0.34 | 0.19 | 0.21 | 0.30 | 0.33 |
|  | **QFFs** | 18.10 | 12.51 | 11.50 | 1.64 | 1.28 | 1.26 | 1.61 | 0.05 | 0.03 | 0.03 | 0.04 | 0.06 | 0.05 | 0.03 | 0.05 |

Table S46: Comparison of the detection frequencies (%) of the particulate phase (QFFs) and of the sum of gaseous and particulate phases (total) of NPAHs. Only the individual compounds for which differences were found are included

|  |  | Winter | | | Summer | | |
| --- | --- | --- | --- | --- | --- | --- | --- |
|  |  | R | UB | T | R | UB | T |
| 1-NNAP | QFFs | 50 | 14 | 100 | 0 | 0 | 0 |
|  | Total | 100 | 100 | 100 | 100 | 100 | 100 |
| 2-NNAP | QFFs | 0 | 0 | 100 | 0 | 0 | 63 |
|  | Total | 100 | 100 | 100 | 100 | 100 | 100 |
| 5-NACE | QFFs | 0 | 0 | 0 | 0 | 0 | 0 |
|  | Total | 75 | 0 | 100 | 100 | 100 | 100 |
| 3-NPHE | QFFs | 50 | 0 | 100 | 0 | 0 | 100 |
|  | Total | 75 | 0 | 100 | 100 | 0 | 100 |
| 2-NFLT | QFFs | 100 | 100 | 100 | 0 | 0 | 100 |
|  | Total | 100 | 100 | 100 | 100 | 100 | 100 |
| 1-NPYR | QFFs | 88 | 100 | 100 | 0 | 0 | 100 |
|  | Total | 100 | 100 | 100 | 75 | 100 | 100 |
| 2-NPYR | QFFs | 100 | 100 | 100 | 0 | 0 | 100 |
|  | Total | 100 | 100 | 100 | 25 | 0 | 100 |
| Σ9NPAHs | QFFs | 100 | 100 | 100 | 0 | 0 | 100 |
|  | Total | 100 | 100 | 100 | 100 | 100 | 100 |

Table S47: Comparison of the detection frequencies (%) of the particulate phase (QFFs) and of the sum of gaseous and particulate phases (total) of OPAHs.

|  |  | Winter | | | Summer | | |
| --- | --- | --- | --- | --- | --- | --- | --- |
|  |  | R | UB | T | R | UB | T |
| 1,4-O_2_NAP | QFFs | 50 | 43 | 100 | 88 | 0 | 88 |
|  | Total | 100 | 100 | 100 | 100 | 100 | 100 |
| 1(CHO)NAP | QFFs | 38 | 43 | 100 | 63 | 0 | 38 |
|  | Total | 100 | 100 | 100 | 100 | 100 | 100 |
| 9-OFLN | QFFs | 25 | 14 | 43 | 0 | 0 | 0 |
|  | Total | 100 | 100 | 100 | 100 | 100 | 100 |
| 9,10-O_2_ANT | QFFs | 100 | 57 | 100 | 0 | 0 | 75 |
|  | Total | 100 | 100 | 100 | 100 | 88 | 100 |
| 1,4-O_2_ANT | QFFs | 75 | 0 | 57 | 0 | 0 | 0 |
|  | Total | 100 | 43 | 71 | 0 | 0 | 0 |
| 9,10-O_2_PHE | QFFs | 88 | 0 | 57 | 0 | 0 | 88 |
|  | Total | 100 | 86 | 71 | 0 | 0 | 88 |
| BaOFLN | QFFs | 100 | 100 | 100 | 63 | 13 | 100 |
|  | Total | 100 | 100 | 100 | 100 | 75 | 100 |
| BbOFLN | QFFs | 100 | 100 | 100 | 88 | 0 | 100 |
|  | Total | 100 | 100 | 100 | 100 | 50 | 100 |
| BAN | QFFs | 100 | 100 | 100 | 100 | 0 | 100 |
|  | Total | 100 | 100 | 100 | 100 | 25 | 100 |
| 7,12-O_2_BAA | QFFs | 100 | 100 | 100 | 13 | 0 | 100 |
|  | Total | 100 | 100 | 100 | 100 | 75 | 100 |
| 5,12-O_2_NAC | QFFs | 100 | 100 | 100 | 100 | 0 | 0 |
|  | Total | 100 | 100 | 100 | 100 | 88 | 88 |
| **Σ11OPAHs** | QFFs | 100 | 100 | 100 | 100 | 13 | 100 |
|  | Total | 100 | 100 | 100 | 100 | 100 | 100 |

Table S48: Comparison of the winter-to summer ratios of the average concentrations when considering only the particulate (QFFs) or the total (Total) concentrations.

|  | R | | UB | | T | |
| --- | --- | --- | --- | --- | --- | --- |
|  | QFFs | Total | QFFs | Total | QFFs | Total |
| 1-NNAP |  | 2.56 |  | 1.10 |  | 4.57 |
| 2-NNAP |  | 5.32 |  | 2.93 | 5.95 | 5.49 |
| 5-NACE |  | 0.13 |  |  |  | 0.13 |
| 3-NPHE |  | 5.46 |  |  | 31.57 | 4.59 |
| 2-NFLT |  | 42.09 |  | 49.93 | 217.30 | 152.29 |
| 3-NFLT |  |  |  |  | 113.08 | 113.08 |
| 1-NPYR |  | 29.00 |  | 23.56 | 38.55 | 34.82 |
| 2-NPYR |  | 43.53 |  |  | 471.48 | 471.48 |
| 7-NBAA |  |  |  |  | 421.63 | 421.63 |
| Σ9NPAHs |  | 11.51 |  | 6.03 | 294.45 | 30.24 |
| 1,4-O_2_NAP | 1.75 | 18.33 |  | 5.52 | 33.81 | 20.48 |
| 1(CHO)NAP | 3.72 | 32.10 |  | 7.78 | 45.07 | 24.55 |
| 9-OFLN |  | 14.11 |  | 6.60 |  | 19.55 |
| 9,10-O_2_ANT |  | 10.95 |  | 8.97 | 198.31 | 38.43 |
| 1,4-O_2_ANT |  |  |  |  |  |  |
| 9,10-O_2_PHE |  |  |  |  | 26.77 | 185.25 |
| BaOFLN | 103.61 | 10.87 | 179.32 | 25.20 | 199.47 | 95.80 |
| BbOFLN | 85.64 | 21.73 |  | 35.43 | 245.25 | 181.13 |
| BAN | 63.34 | 40.95 |  | 43.25 | 211.97 | 211.97 |
| 7,12-O_2_BAA | 18.10 | 19.76 |  | 22.87 | 137.45 | 94.31 |
| 5,12-O_2_NAC | 82.24 | 53.76 |  | 288.64 |  | 2677.14 |
| Σ11OPAHs | 97.93 | 21.10 | 11244.63 | 11.10 | 164.57 | 38.54 |

Table S49: Traffic to urban background ratios of NPAHs and OPAHs based on total (gaseous and particulate) concentrations

|  | Winter | | | | | | Summer | | | | | |
| --- | --- | --- | --- | --- | --- | --- | --- | --- | --- | --- | --- | --- |
|  | N | Min | Max | Average | Median | SD | N | Min | Max | Average | Median | SD |
| **1-NNAP** | 7 | 0.71 | 4.57 | 2.36 | 2.14 | 1.17 | 8 | 0.44 | 0.77 | 0.56 | 0.55 | 0.11 |
| **2-NNAP** | 7 | 0.41 | 2.37 | 1.37 | 1.40 | 0.58 | 8 | 0.50 | 1.26 | 0.78 | 0.76 | 0.25 |
| **5-NACE** | 0 |  |  |  |  |  | 8 | 0.76 | 1.46 | 1.18 | 1.18 | 0.23 |
| **9-NANT** | 7 | 2.05 | 13.76 | 7.22 | 6.98 | 3.81 | 0 |  |  |  |  |  |
| **3-NPHE** | 0 |  |  |  |  |  | 0 |  |  |  |  |  |
| **2-NFLT** | 7 | 1.39 | 2.29 | 1.95 | 1.85 | 0.33 | 8 | 0.32 | 1.90 | 0.76 | 0.65 | 0.49 |
| **3-NFLT** | 3 | 1.99 | 2.44 | 2.17 | 2.09 | 0.24 | 0 |  |  |  |  |  |
| **1-NPYR** | 7 | 2.09 | 4.37 | 2.72 | 2.45 | 0.79 | 8 | 0.94 | 4.86 | 1.94 | 1.81 | 1.26 |
| **2-NPYR** | 7 | 0.79 | 2.72 | 1.66 | 1.47 | 0.65 | 0 |  |  |  |  |  |
| **7-NBAA** | 3 | 2.43 | 7.23 | 4.87 | 4.96 | 2.40 | 0 |  |  |  |  |  |
| **Σ9NPAHs** | 7 | 2.20 | 5.09 | 3.32 | 2.62 | 1.19 | 8 | 0.57 | 0.86 | 0.69 | 0.70 | 0.09 |
| **1,4-O_2_NAP** | 7 | 0.38 | 0.88 | 0.64 | 0.65 | 0.15 | 8 | 0.01 | 0.25 | 0.15 | 0.14 | 0.09 |
| **1(CHO)NAP** | 7 | 0.46 | 2.13 | 1.28 | 1.38 | 0.51 | 8 | 0.32 | 0.50 | 0.43 | 0.46 | 0.07 |
| **9-OFLN** | 7 | 0.35 | 0.52 | 0.44 | 0.47 | 0.07 | 8 | 0.12 | 0.20 | 0.15 | 0.14 | 0.02 |
| **9,10-O_2_ANT** | 7 | 0.48 | 1.52 | 0.98 | 0.98 | 0.34 | 7 | 0.17 | 0.26 | 0.21 | 0.20 | 0.04 |
| **1,4-O_2_ANT** | 1 |  |  | 0.67 | 0.67 |  | 0 |  |  |  |  |  |
| **9,10-O_2_PHE** | 4 | 0.18 | 1.19 | 0.57 | 0.46 | 0.47 | 0 |  |  |  |  |  |
| **BaOFLN** | 7 | 0.79 | 2.58 | 1.63 | 1.40 | 0.66 | 6 | 0.19 | 1.23 | 0.49 | 0.35 | 0.38 |
| **BbOFLN** | 7 | 0.16 | 1.85 | 1.28 | 1.58 | 0.68 | 4 | 0.10 | 0.20 | 0.14 | 0.13 | 0.04 |
| **BAN** | 7 | 0.42 | 1.16 | 0.79 | 0.78 | 0.22 | 2 | 0.10 | 0.17 | 0.13 | 0.13 | 0.05 |
| **7,12-O_2_BAA** | 7 | 1.08 | 2.32 | 1.71 | 1.77 | 0.39 | 6 | 0.20 | 0.64 | 0.38 | 0.35 | 0.15 |
| **5,12-O_2_NAC** | 7 | 1.04 | 3.37 | 1.74 | 1.50 | 0.78 | 6 | 0.13 | 0.43 | 0.22 | 0.18 | 0.11 |
| **Σ11OPAHs** | 7 | 0.56 | 1.53 | 0.83 | 0.65 | 0.34 | 8 | 0.17 | 0.29 | 0.23 | 0.22 | 0.04 |

Table S50: Urban background to rural ratios of NPAHs and OPAHs based on total (gaseous and particulate) concentrations

|  | Winter | | | | | | Summer | | | | | |
| --- | --- | --- | --- | --- | --- | --- | --- | --- | --- | --- | --- | --- |
|  | N | Min | Max | Average | Median | SD | N | Min | Max | Average | Median | SD |
| **1-NNAP** | 7 | 1.70 | 8.45 | 4.36 | 3.83 | 2.31 | 8 | 6.23 | 13.27 | 9.32 | 8.58 | 2.48 |
| **2-NNAP** | 7 | 1.34 | 4.86 | 2.79 | 2.96 | 1.18 | 8 | 3.37 | 6.14 | 5.01 | 5.25 | 1.10 |
| **5-NACE** | 0 |  |  |  |  |  | 8 | 3.14 | 8.98 | 5.79 | 5.23 | 2.15 |
| **9-NANT** | 7 | 0.51 | 11.11 | 2.78 | 1.11 | 3.75 | 0 |  |  |  |  |  |
| **3-NPHE** | 0 |  |  |  |  |  | 0 |  |  |  |  |  |
| **2-NFLT** | 7 | 1.45 | 7.29 | 4.37 | 4.89 | 2.49 | 8 | 2.74 | 9.25 | 4.89 | 4.49 | 2.01 |
| **3-NFLT** | 3 | 4.97 | 17.18 | 12.44 | 15.15 | 6.54 | 0 |  |  |  |  |  |
| **1-NPYR** | 7 | 4.20 | 17.31 | 11.89 | 12.07 | 5.03 | 6 | 10.95 | 26.59 | 20.28 | 22.22 | 5.75 |
| **2-NPYR** | 7 | 6.95 | 29.45 | 16.48 | 18.09 | 7.93 | 0 |  |  |  |  |  |
| **7-NBAA** | 3 | 3.51 | 13.20 | 7.67 | 6.30 | 4.99 | 0 |  |  |  |  |  |
| **Σ9NPAHs** | 7 | 1.40 | 9.42 | 3.67 | 2.66 | 2.82 | 8 | 5.80 | 9.31 | 7.35 | 7.33 | 1.13 |
| **1,4-O_2_NAP** | 7 | 1.45 | 3.51 | 2.49 | 2.71 | 0.81 | 8 | 3.18 | 23.77 | 8.77 | 5.28 | 6.99 |
| **1(CHO)NAP** | 7 | 1.12 | 3.98 | 2.13 | 1.81 | 1.02 | 8 | 4.37 | 13.22 | 8.56 | 8.21 | 2.81 |
| **9-OFLN** | 7 | 2.92 | 7.53 | 4.54 | 4.28 | 1.59 | 8 | 6.61 | 12.89 | 10.55 | 10.76 | 1.87 |
| **9,10-O_2_ANT** | 7 | 2.08 | 17.65 | 5.68 | 2.87 | 5.60 | 7 | 4.83 | 9.53 | 6.43 | 6.10 | 1.51 |
| **1,4-O_2_ANT** | 3 | 2.66 | 6.18 | 4.23 | 3.86 | 1.79 | 0 |  |  |  |  |  |
| **9,10-O_2_PHE** | 6 | 1.40 | 63.85 | 13.24 | 2.83 | 24.86 | 0 |  |  |  |  |  |
| **BaOFLN** | 7 | 1.92 | 24.62 | 7.68 | 4.90 | 8.14 | 6 | 1.12 | 10.37 | 5.33 | 5.15 | 3.01 |
| **BbOFLN** | 7 | 1.38 | 35.68 | 9.77 | 4.94 | 12.27 | 4 | 6.32 | 68.33 | 23.48 | 9.64 | 29.97 |
| **BAN** | 7 | 1.59 | 31.91 | 10.17 | 4.34 | 11.10 | 2 | 31.11 | 53.35 | 42.23 | 42.23 | 15.73 |
| **7,12-O_2_BAA** | 7 | 1.29 | 17.96 | 5.98 | 3.36 | 6.13 | 6 | 3.39 | 14.69 | 7.74 | 7.29 | 3.99 |
| **5,12-O_2_NAC** | 7 | 1.68 | 32.46 | 9.82 | 5.35 | 11.08 | 7 | 1.58 | 5.87 | 3.58 | 3.27 | 1.39 |
| **Σ11OPAHs** | 7 | 1.56 | 8.40 | 3.71 | 2.83 | 2.26 | 8 | 4.64 | 13.34 | 7.63 | 6.43 | 2.80 |

Figure S1: Detection frequencies of individual NPAHs in winter (a) and summer (b)

a)

b)

Figure S2: Detection frequencies of individual OPAHs in winter (a) and summer (b)

Figure S3: Temporal variations of the NPAHs composition profile. L, B, W, S, R, UB, T denote Brno, Ljubljana, winter, summer, rural, urban background and traffic, respectively. Due to low detection frequencies, some figures for some specific sites in summer are not shown

Figure S4: Temporal variations of the OPAHs composition profile. L, B, W, S, R, UB, T denote Ljubljana, Brno, winter, summer, rural, urban background and traffic, respectively. Due to low detection frequencies, some figures for some specific sites in summer are not shown

Figure S5: Caracterisation of the vehicle technologies among personal cars (both diesel and petrol) in Ljubljana and Brno


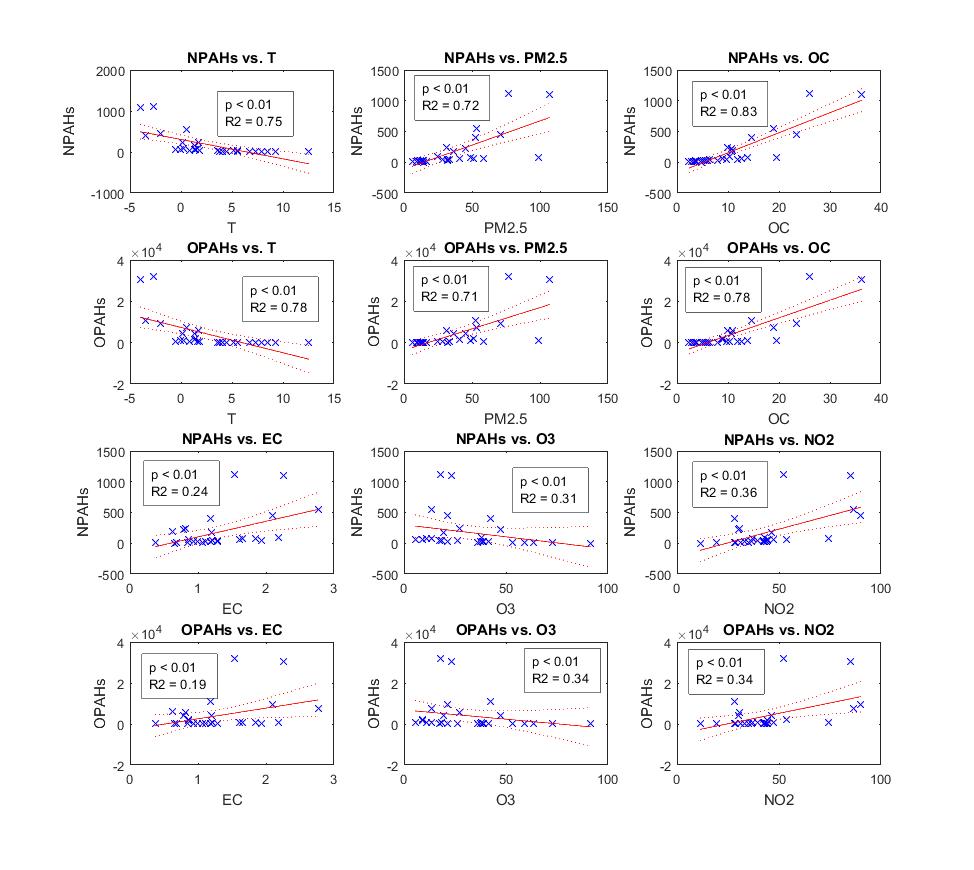


Figure S6: Relationships between Σ_9_NPAHs (in pg m^-3^) and Σ_11_OPAHs (in pg m^-3^) with temperature (T, in °C), PM_2.5_, OC, EC, O_3_ and NO_2_ (all in µg m^-3^) at the Brno UB site in winter


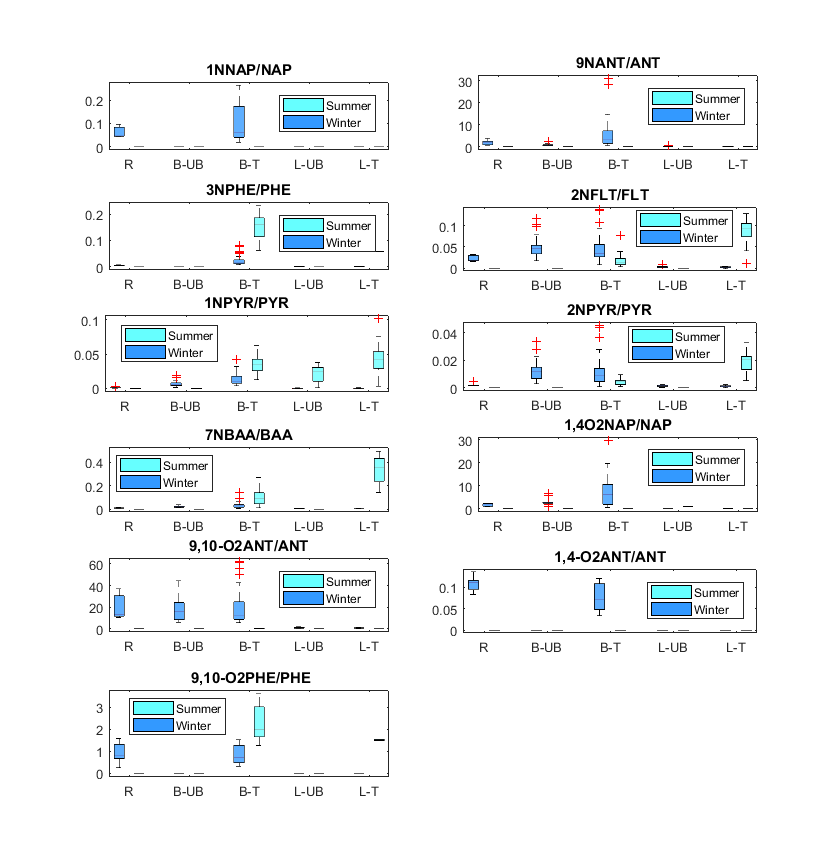


Figure S7: Ratios (unitless) between individual NOPAHs and their parent PAHs. B, L, R, UB and T denote Brno, Ljubljana, rural, urban background and traffic, respectively


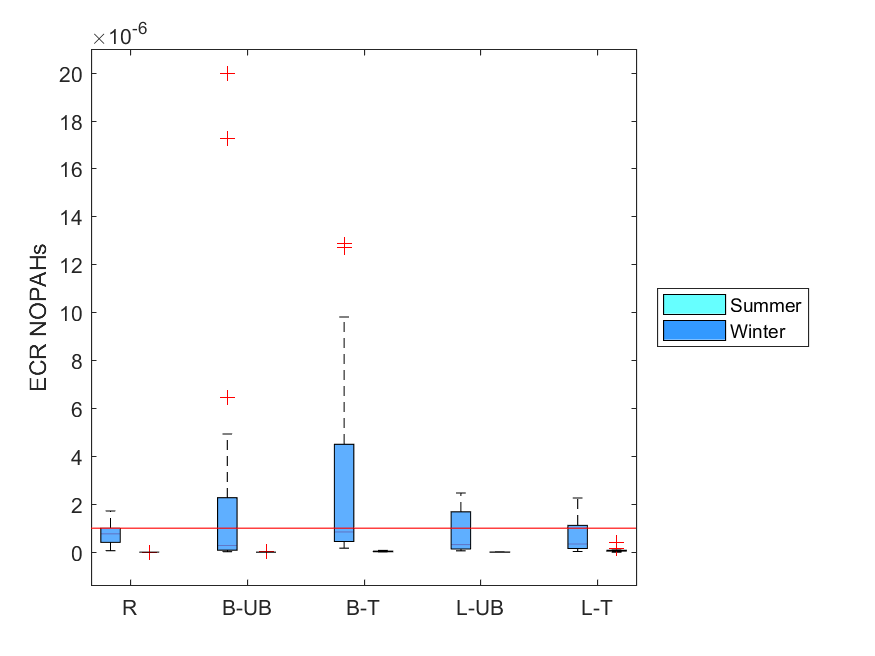


Figure S8: Boxplots of ECR related to particulate NOPAHs


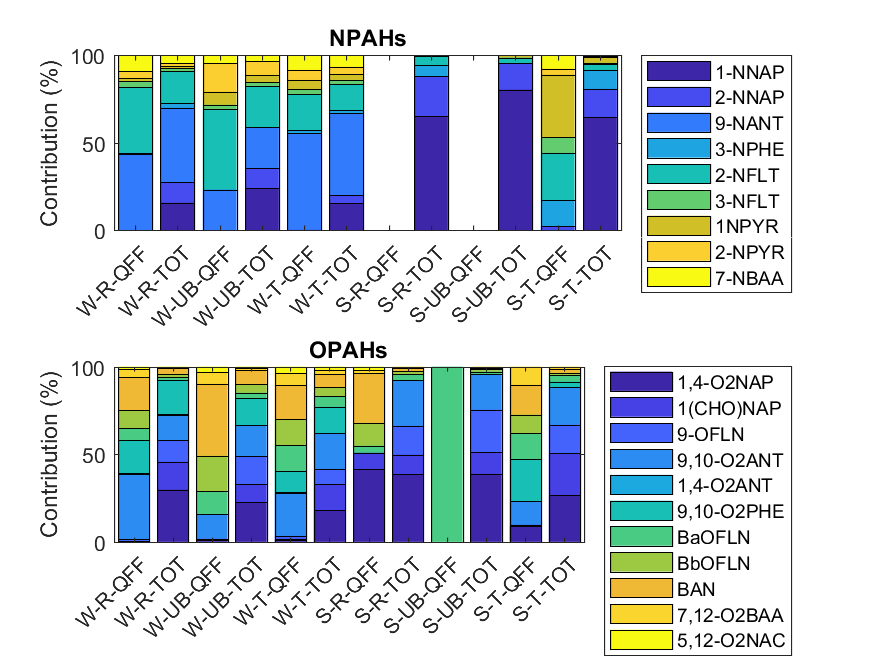


Figure S9: Comparison of the composition profile when considering only the particulate (QFF) or the total (TOT) concentrations

a)
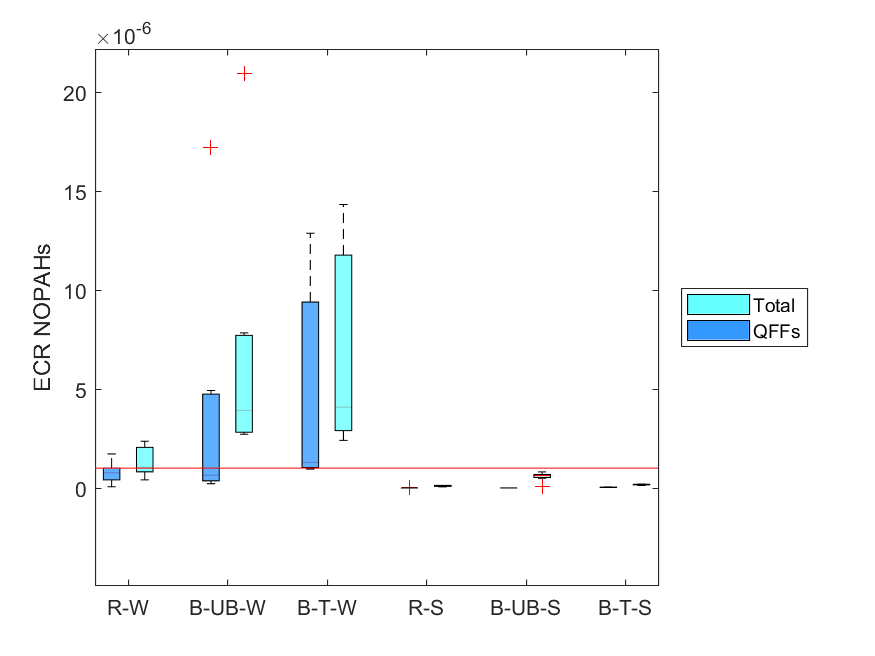


b)
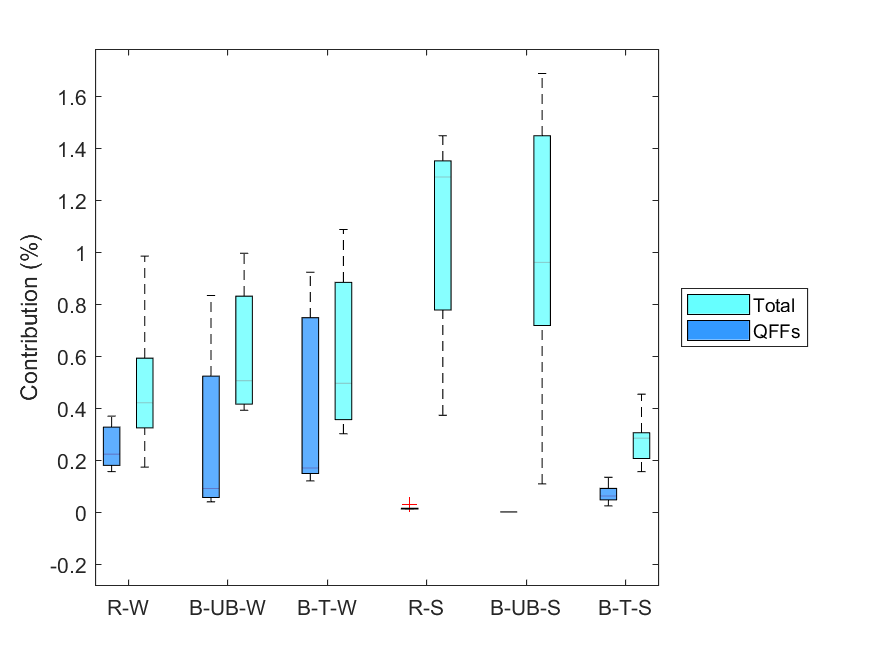


Figure S10: Comparison of the cancer risks due to NOPAHs when the particulate (QFFs) or total (Total) concentrations are considered (a) and contribution of NOPAHs to the cancer risks due to PACs (b). The horizontal red line indicates the acceptable lifetime carcinogenic risk

**References**

Durant, J.L., Busby, W.F., Lafleur, A.L., Penman, B.W., Crespi, C.L., 1996. Human cell mutagenicity of oxygenated, nitrated and unsubstituted polycyclic aromatic hydrocarbons associated with urban aerosols. Mutat. Res. - Genet. Toxicol. 371, 123–157. https://doi.org/10.1016/S0165-1218(96)90103-2

Nisbet, I.C.T., LaGoy, P.K., 1992. Toxic equivalency factors (TEFs) for polycyclic aromatic hydrocarbons (PAHs). Regul. Toxicol. Pharmacol. 16, 290–300. https://doi.org/10.1016/0273-2300(92)90009-X

OEHHA, 1994. Benzo(a)pyrene as a toxic air contaminant [WWW Document]. URL http://www.arb.ca.gov/toxics/id/summary/bap.pdf (accessed 1.25.21).

USEPA, 2010. Development of a Relative Potency Factor (Rpf) Approach for Polycyclic Aromatic Hydrocarbon (PAH) Mixtures (External Review Draft). Wahington. https://doi.org/DCEPA/635/R-08/012A
